# A unified solvatochromic–computational–machine–learning approach for solvent property prediction based on iodine color response

**DOI:** 10.1039/d6sc03325c

**Published:** 2026-08-24

**Authors:** Evgeniy G. Gordeev, Liana A. Arakelyan, Daria M. Arkhipova, Alexander A. Oganov, Kirill V. Vokhmintsev, Valentine P. Ananikov

**Affiliations:** a N. D. Zelinsky Institute of Organic Chemistry, Russian Academy of Sciences 119991 Leninsky Prospect, 47 Moscow Russia https://AnanikovLab.ru val@ioc.ac.ru

## Abstract

Solvent effects govern the majority of chemical transformations, yet experimentally accessible and universally applicable tools for predicting solvating ability remain limited. Here, we introduce iSolv, a simple but information-rich solvatochromic descriptor derived from the color of diluted iodine solutions in organic solvents. Using a combined experimental–computational approach, we demonstrate that iSolv directly reflects the nature and strength of intermolecular interactions, including dispersion interaction, halogen bonding, donor–acceptor interactions and hydrogen bonding. We establish clear correlations between iSolv and key electronic, physicochemical, and empirical solvent parameters, such as HOMO energy, donor number, log *P*, Kamlet–Taft parameters, and reaction rates in diverse organic transformations. Machine-learning modelling confirms that iSolv enables predictive assessment of solvent behavior using easily accessible input parameters. Unlike existing multi-parameter solvent scales, iSolv offers a low-cost, rapid, and visually interpretable method applicable across chemical research, education, and chemical technology. The practical and computational relevance of iSolv positions it as a versatile tool for solvent selection, reaction optimization, and broader chemical informatics. This work introduces a convenient indicator-based strategy for assessing solvating ability and lays the foundation for integrating visual color response into the digital chemistry framework.

## Abstract 2

A new quantitative descriptor (iSolv) of organic-solvent solvating ability is derived from the color of iodine solutions using a simple, rapid digital-photography procedure.

## Graphical abstract

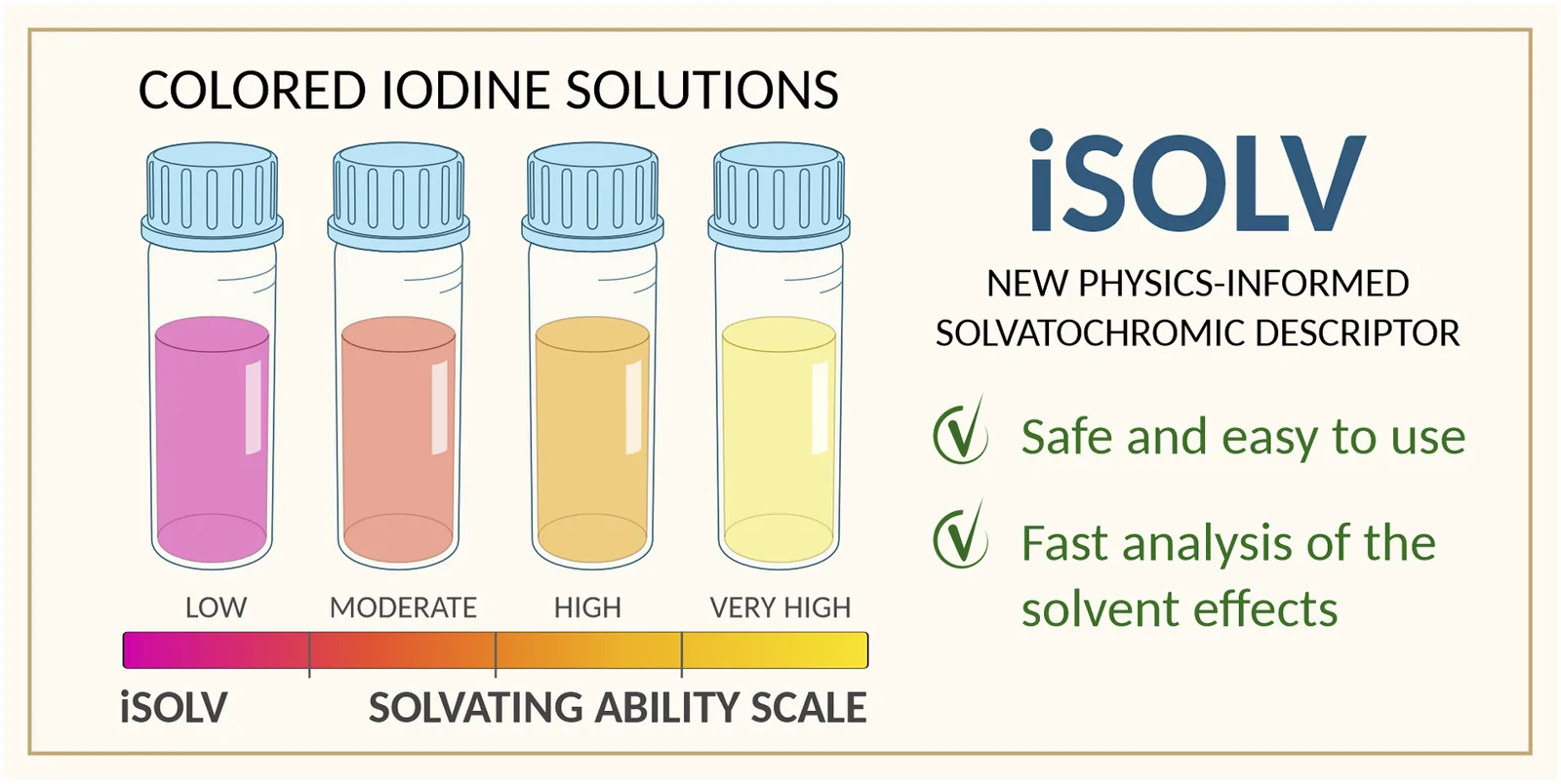

## 1 Introduction

Solvents play a decisive role in chemical science, as the overwhelming majority of reactions occur in liquid media. The solvent can profoundly affect reaction pathways, kinetic and thermodynamic parameters, selectivity, equilibrium position, and the formation of by-products. Key influence on reactivity was first recognized in the late 19th century,^1^ and since then, solvent choice has become a key technological factor in both efficiency and environmental safety of chemical processes.^2,3^

The immense variety of chemical solvents, including mixtures with emergent properties, makes predicting their behavior challenging, and today solvent effects are one of the central problems of theoretical research and molecular modeling.^4^ Numerous classifications have been proposed – based on structure, polarity, and acid–base characteristics – but none adequately captures the overall influence of the solvent medium on chemical transformations.^5^ The most fundamental characteristic defining solvent impact is its general solvating ability, a collective property governed by a complex interplay of intermolecular interactions at the molecular level.^6^ Importantly, macroscopic parameters such as the dielectric constant fail to accurately reflect microsolvation phenomena, which depend on subtle electronic and structural features of solvent and solute molecules.^7–19^

Early systematic studies, including N. A. Menshutkin's investigations of quaternary ammonium salt formation,^20,21^ demonstrated that reaction rates strongly depend on solvent polarity and structure, a finding further validated by molecular modeling.^22–24^

Solvent effects are particularly intricate in reactions such as the Diels–Alder cycloaddition, where stereoselectivity and regioselectivity of this process can be strongly modulated by solvent polarity, polarizability, and dispersion interactions.^25,26^ When water is used as the solvent, the cycloaddition can be significantly accelerated due to the hydrophobic effect, which can also play a key role in determining the stereoselectivity of the reaction.^27–30^ Specific interactions with solvent molecules can affect the rate and selectivity of pericyclic reactions.^31,32^

Solvent polarity and microsolvation conditions also determine mechanistic preferences, as illustrated by the S_N_1/S_N_2 competition.^6,33–35^ Moreover, the solvent environment critically affects transition metal-catalyzed coupling reactions, which underpin modern pharmaceutical and fine chemical synthesis.^36^

In this study, a methodology is introduced to address a longstanding challenge: direct, rapid, and experimentally accessible estimation of solvating ability. Traditionally, quantitative solvent descriptors have relied on multi-parameter models that include polarity, donor–acceptor, and hydrogen-bonding properties as parameters.^37,38^ While powerful, these models are often impractical for everyday laboratory use. A simpler, visual, and yet scientifically grounded approach would be of high value. Solvatochromism, the color change of a solute depending on the surrounding medium, offers such a pathway.^39^ Organic indicator substances with pronounced solvatochromism have been proposed, which enable the determination of the polarity of the solvent and microenvironment not only in organic synthesis but also in biochemical applications.^40–49^ Such solvatochromic agents can be used to study the properties of not only molecular organic solvents but also deep eutectic solvents and ionic liquids, which represent promising media for fine organic synthesis.^50–55^

In this work, we propose to use the solvatochromism of iodine to assess the solvating ability of solvents. The first studies on the solvatochromism of iodine appeared as early as the 19th century and continued actively into the first half of the 20th century.^56–60^ It was later shown that the solvatochromic effect of iodine reflects different types of solvent–solute interactions.^61–64^ Studies with the quantitative analysis of solvatochromic effects in iodine solutions are based on spectrophotometric data.^65–68^ In such studies, the absorption maxima in iodine solutions are correlated with the nature of the solvent. These correlations make it possible to divide solvents into groups, although within each group of solvents, the absorption maxima may be characterized by fairly similar wavelength values. Moreover, solvents with different properties belonging to different groups may exhibit identical wavelength values at the absorption maximum.^69^ To our knowledge, these observations have not previously been translated into a photograph-derived, continuous numerical descriptor that has been validated against molecular, empirical, and kinetic data.

Iodine solvatochromism itself is not new. The novelty of the present work lies in converting this established color response into a defined, photograph-derived numerical descriptor and validating it across computational, physicochemical, kinetic, and machine-learning analyses. Accordingly, in this work, we introduce iSolv – a new solvatochromic descriptor derived from the color of diluted iodine solutions (Fig. 1 and 2). We demonstrate that iSolv not only reflects intermolecular interaction characteristics, but also correlates with reaction rates and solvent-dependent behavior, enabling rapid and approximate prediction of solvating ability. The iSolv calculation relies on the Hue value of the digital HSB (Hue, Saturation, and Brightness) color scale, which can be very easily obtained from a digital photograph taken with any digital camera, such as a mobile phone camera. Within the solvent set examined, iSolv provides qualitative discrimination of solvent classes comparable to that obtained from literature visible-band absorption maxima, while remaining convenient for digital processing and automated image analysis.

**Fig. 1:**
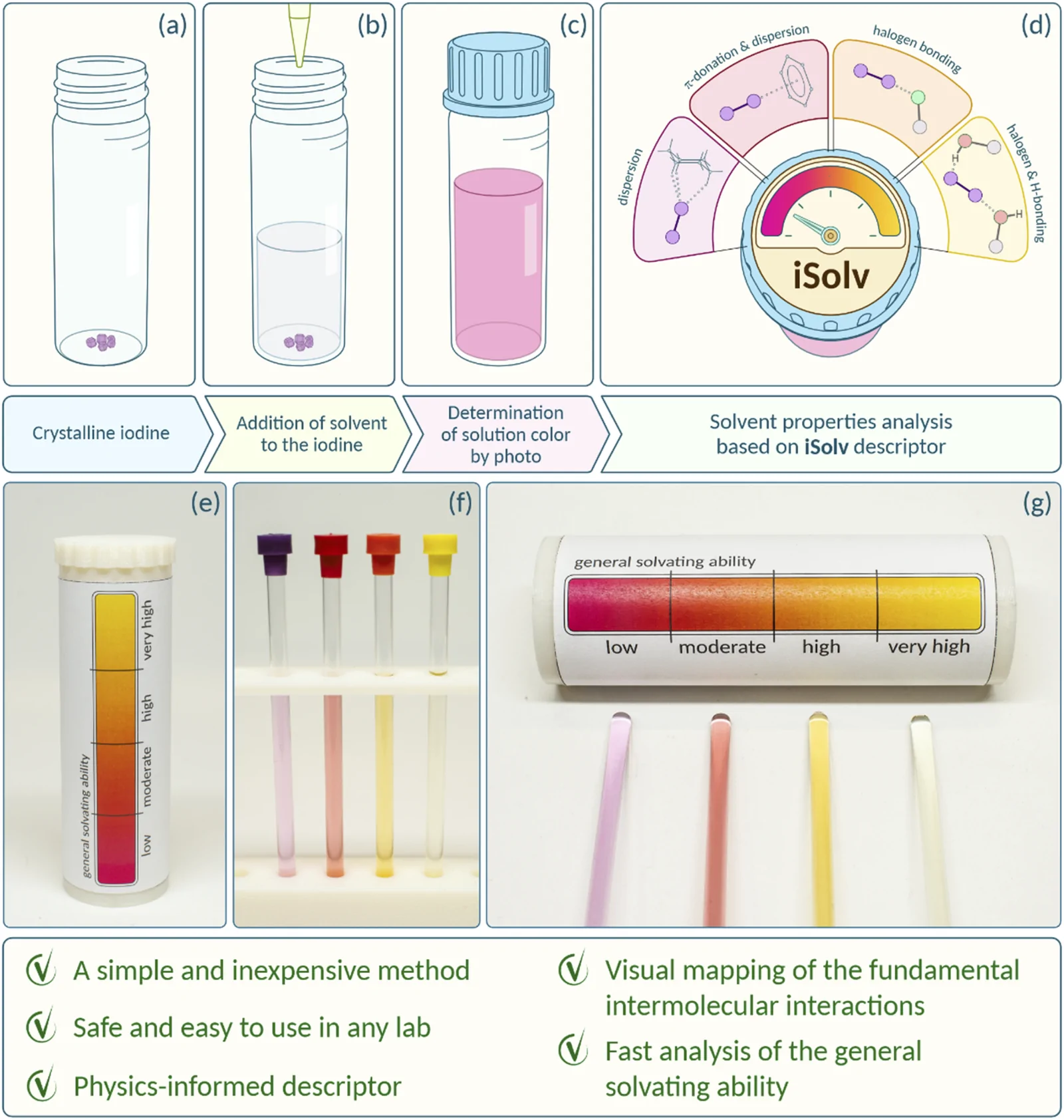
Schematic representation of the methodology for determining solvent properties using the new descriptor iSolv: (a) a glass vessel with a small amount of crystalline iodine; (b) addition of the studied solvent into the iodine-containing vessel; (c) colored iodine solution; (d) determination of the solvent's general solvating ability based on the color of the solution (descriptor iSolv) using a color scale; (e) a color scale for the practical determination of general solvating ability on the basis of the color of standard-concentration iodine solutions; (f) standard-concentration iodine solutions in ampoules prepared for use with the color scale; (g) determination of the solvating ability of various solvents by comparing the colors of iodine solutions with the corresponding regions of the color scale.

**Fig. 2:**
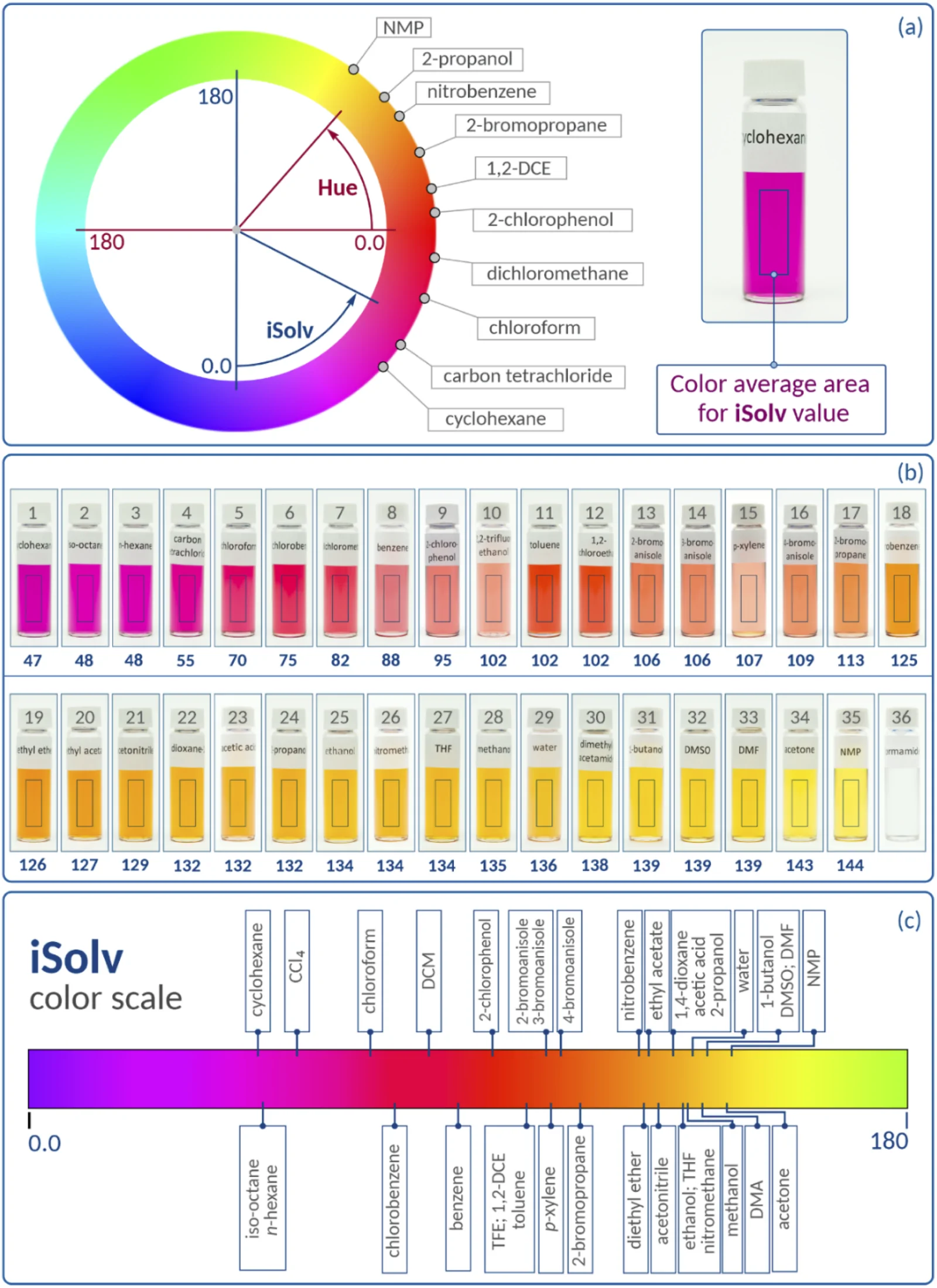
(a) HSB color wheel showing the Hue and iSolv values for colors of some solutions, and a magnified photograph of one of the solutions with the color averaging area; (b) iodine solutions in various organic solvents with color-averaging areas and iSolv values; formamide was excluded because no suitable color was obtained; (c) iSolv color scale with the positions of all studied solvents.

The procedure of the iSolv usage is simple and affordable. At first a small amount of crystalline iodine is placed into a transparent glass vessel, serving as an indicator substance (Fig. 1a). Upon addition of the solvent under investigation (Fig. 1b), iodine dissolves, forming a colored solution whose appearance reflects the specific interactions between iodine and solvent molecules (Fig. 1c and d). It is demonstrated that different solvents produce solutions with distinct colors, covering a broad spectrum that visually correlates with their solvating ability. Finally, it is shown (Fig. 1e–g) that these colors can be compared with a reference color-based iSolv scale, enabling rapid assignment of the solvent to a corresponding solvating ability category. This visually guided methodology forms the basis of the iSolv concept as a simple, experimentally accessible tool for characterizing solvent solvating ability. For quantitative analysis, the iSolv value can be easily obtained from the Hue value of an iodine solution, determined from a digital photograph of the solution using the HSB color scale.

This study systematically validates iSolv as a new solvent descriptor, explores its molecular-level origin (Section 2.1), experimental correlations (Section 2.2), machine-learning-based predictive capability (Section 2.3), its potential for practical chemical applications (Section 2.4) and ready-to-use software implementation (Conclusions and SI Section 5).

## 2 Results and discussion

### 2.1 Color of an iodine solution as a descriptor of the electronic characteristics of a solvent molecule

To study solvatochromism in iodine solutions, 36 diluted solutions of equal concentration were prepared in a chemically diverse set of molecular solvents (Fig. 1, 2a, b and Table S1). Each glass vial containing solution was photographed under controlled lighting conditions. A rectangular area was selected inside the photograph, within which the color of the solution was averaged. To obtain a numerical representation of the solution color, the HSB scale (Hue, Saturation, and Brightness) was utilized, where color is defined *via* a color wheel. The primary parameter characterizing the color is Hue, which is numerically equivalent to the angle on the color wheel (Fig. 2a). The Hue value can be obtained manually with the aid of a graphic editor. First, the color is averaged over a rectangular selection within the image, and then the Hue value is read using the color picker tool. The HSB color model is highly convenient for color analysis because it is oriented toward human color perception. In this model, the actual color – the main parameter considered in this work – is determined by a single parameter, Hue, and it is this parameter that correlates with the properties of solvents. The remaining parameters (Saturation and Brightness) are determined by the concentration of the solution. Thus, it is Hue that depends on the nature of intermolecular interactions between iodine and solvent molecules. Importantly, Hue, Saturation, and Brightness are independent parameters, which is crucial because small variations in concentration have only a minor effect on Hue. For instance, varying the amount of dissolved iodine by roughly 1.5-fold does not appreciably affect the Hue value and, hence, the iSolv value (Fig. S21).

The RGB model is less suitable for the purposes of this study, since in this model all three color channels are interdependent, and even a slight change in color leads to changes in all RGB channels (see Table S1 for RGB and HSB model comparison).

As shown in Fig. 2a, all the solution colors investigated in this project cluster in the right half of the color wheel. For analytical convenience, the reference point for measuring angles on the color wheel was shifted by 90° to prevent solution color values from crossing zero. The angle measured from this new reference point constitutes the iSolv value; hence iSolv = [(Hue + 90°) mod 360°]; for the Hue range observed in this study, the resulting iSolv values span 0–180°. All solution colors can be conveniently arranged not only on a color wheel but also on a linear scale (Fig. 2c). The use of digital photography to determine solution color enables rapid analysis through mobile photography and specialized software developed for this project (see the SI, Section 5). It is worth noting that photography does not require a specialized camera; images can be captured using a mobile phone camera. Tests performed with 10 different mobile phone cameras on 5 solutions demonstrated that the iSolv values are highly reproducible regardless of the camera used (Tables S11–S21).

According to the color of the solutions, the solvents were divided into four groups on the basis of their solvating ability (Fig. 1g). The first group consists of solvents with low solvating ability, which primarily participate in weak dispersion intermolecular interactions, such as those involving hydrocarbons, which form violet-colored iodine solutions. The second group (moderate solvating ability) comprises solvents whose molecules possess easily polarizable π-electrons and/or *n*-electrons of lone electron pairs, enabling them to engage in not only stronger dispersion interactions but also donor–acceptor interactions through the transfer of π/*n*-electrons to the acceptor molecule. The iodine solutions in the second group are characterized by red color. The third group of solvents (high solvating ability), which form orange-yellow solutions, includes compounds capable of forming halogen bonds owing to the presence of lone electron pairs, typically from oxygen atoms. The last group of solvents (very high solvating ability), producing light yellow solutions, encompasses solvents that form all of the aforementioned types of intermolecular interactions, and additionally, some of these solvents are capable of forming hydrogen bonds. The transition from violet to yellow in color of the solution corresponds to an increase in the general solvating ability of the solvents. It should be noted that the spectral characteristics of the iodine molecule in the visible region in various solvents, in particular the absorption maxima of the visible band, are also an effective tool for classifying solvents according to their properties.^69^ For the present solvent set, iSolv provides qualitative class discrimination comparable to that obtained from literature absorption maxima while retaining a direct image-based numerical readout (Fig. 2b).

In organic solvents, I_2_ molecules form intermolecular complexes characterized by the presence of so-called halogen bonds. Halogen bonds are formed due to the existence of regions of positive electrostatic potential in halogen molecules, referred to as σ-holes (Fig. 3a). The σ-hole acts as an electron acceptor, and thus, the I_2_ molecule forms directed halogen bonds with solvent molecules.^70^ The iodine molecule can also act as an electron donor because of the lone electron pairs of each iodine atom, meaning that it can also function as a hydrogen bond acceptor. Although in a complex with an iodine molecule, the solvent more frequently serves as an electron donor, the correlation between the HOMO energy and the LUMO–HOMO gap of an individual solvent molecule with the color of the solution is weak (Table 1 and Fig. S1). Nevertheless, most purple-colored solutions correspond to solvents with low HOMO energies, whereas yellow-colored solutions tend to have noticeably higher HOMO energies for the solvent molecules.

**Fig. 3:**
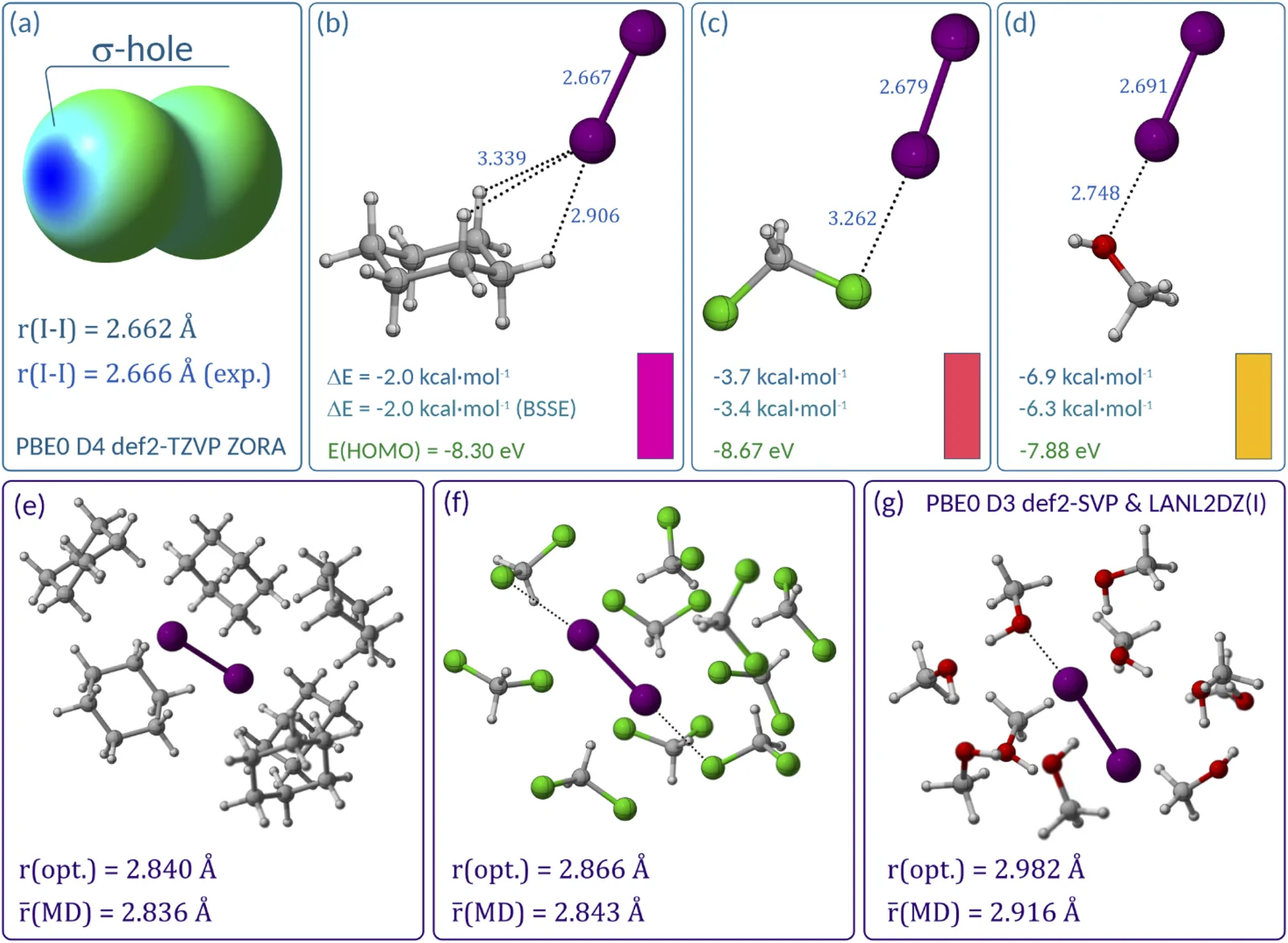
(a) Molecular electrostatic potential for an isolated I_2_ molecule; (b–d) optimized molecular structures of intermolecular complexes of the I_2_ molecule with cyclohexane, dichloromethane, and methanol molecules. Interatomic distances and the color of the corresponding solution are given for each complex; (e–g) optimized molecular structures of an iodine molecule solvated by 6 cyclohexane molecules, 10 dichloromethane molecules, and 10 methanol molecules. For each solvate complex, interatomic distances derived from full optimization of the complexes (*r*(opt.)) and those obtained by averaging data from MD simulations of these complexes are presented (*r̄*(MD)).

**Table 1:** Values of *E*_HOMO_ (eV) for isolated solvent molecules; difference in energies |LUMO–HOMO| (Δ*E*_GAP-1_, eV) for a complex of one solvent molecule with one I_2_ molecule; bonding energy of the I_2_ molecule with the first solvent molecule (Δ*E*_I2-Solv1_, kcal mol^−1^); bonding energy of the I_2_ molecule with the second solvent molecule (Δ*E*_I2-Solv2_, kcal mol^−1^); I–I interatomic distance in the complex of the I_2_ molecule with one solvent molecule (*r*_1_(I–I), Å); I–I interatomic distance in the complex of the I_2_ molecule with two solvent molecules (*r*_2_(I–I), Å); maximum positive values of ESP in the σ-hole region of the I_2_ molecule within the complex with one solvent molecule (*φ*_1_, a.u.). Calculations were performed using the PBE0 D4 def2TZVP ZORA method. For each solution, the iSolv value is listed, the hexadecimal value of the color is listed in the Hex column, and the cells of the column are colored accordingly to match the actual colors of the solutions

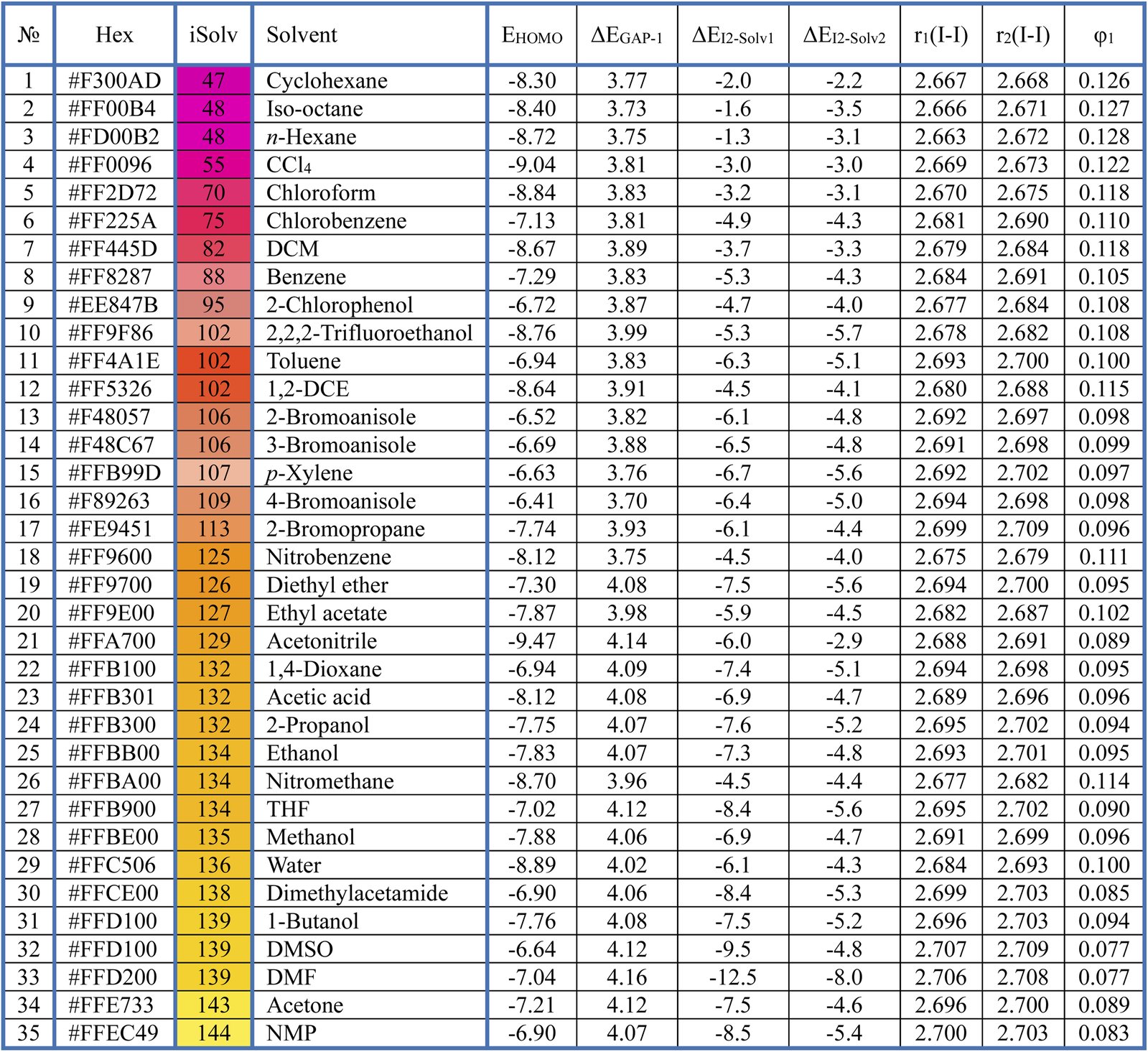

The simplest models that allow analysis of the nature of the interaction between the I_2_ molecule and the solvent involve a complex of one I_2_ molecule with either one or two solvent molecules. Despite the simplicity of this model, experimental evidence suggests that in the condensed phase, one of the atoms in the I_2_ molecule is solvated to a greater extent than the other, especially in benzene media.^71^ Therefore, to adequately reproduce the solvation of the I_2_ molecule, a small number of solvent molecules is sufficient. If the solvent molecule possesses multiple nucleophilic centers, as in the case of NMP, calculations of the bonding energy between the I_2_ molecule and each nucleophilic center were performed. For analysis and discussion, the complex with the maximum bonding energy between the I_2_ molecule and the respective nucleophilic center was utilized. In complexes involving the I_2_ molecule with two solvent molecules, coordination of the I_2_ molecule occurred with the most active nucleophilic centers.

Since the color of solutions containing iodine solvate complexes is caused by an electronic transition associated with charge transfer from the donor molecule to the iodine molecule,^72–74^ a correlation analysis was performed between the color and the magnitude of the LUMO–HOMO energy gap in the solvate complexes, as this parameter should reflect the probability of such a transition: the smaller the difference, the more probable the transition. Indeed, a noticeable correlation is observed between the color of the solution and the magnitude of the energy gap Δ*E*(LUMO–HOMO) in the complex of one I_2_ molecule with one solvent molecule: the transition from violet to yellow is accompanied by an increase in the Δ*E*(LUMO–HOMO) difference (Fig. S2).

The primary contributors to the strength of the halogen bond are electrostatic and dispersion interactions.^75^ As the electron-donating properties of the nucleophilic centers of the organic molecule increase, the bond strength between the I_2_ molecule and the organic molecule increases.^76^ For example, the bonding energy of the I_2_ molecule with a single solvent molecule increases in the series C_6_H_12_ < DCM < MeOH, amounting to −2.0, −3.7, and −6.9 kcal mol^−1^, respectively, without accounting for BSSE (Fig. 3b–d; accounting for BSSE does not significantly affect the bonding energy, hence, for the main set of solvents, the calculation of the I_2_-solvent bonding energy was performed without BSSE). These bonding energy values align well with the results obtained at a higher level of theory (ωB97X-2 def2-TZVP ZORA): −2.4, −3.2, and −6.4 kcal mol^−1^ for the same series of solvents. Overall, solutions with a violet color are characterized by a negligible bonding energy in the complex of one solvent molecule with the iodine molecule, whereas solutions with a yellow color exhibit significantly higher bonding energy (Fig. 4a).

**Fig. 4:**
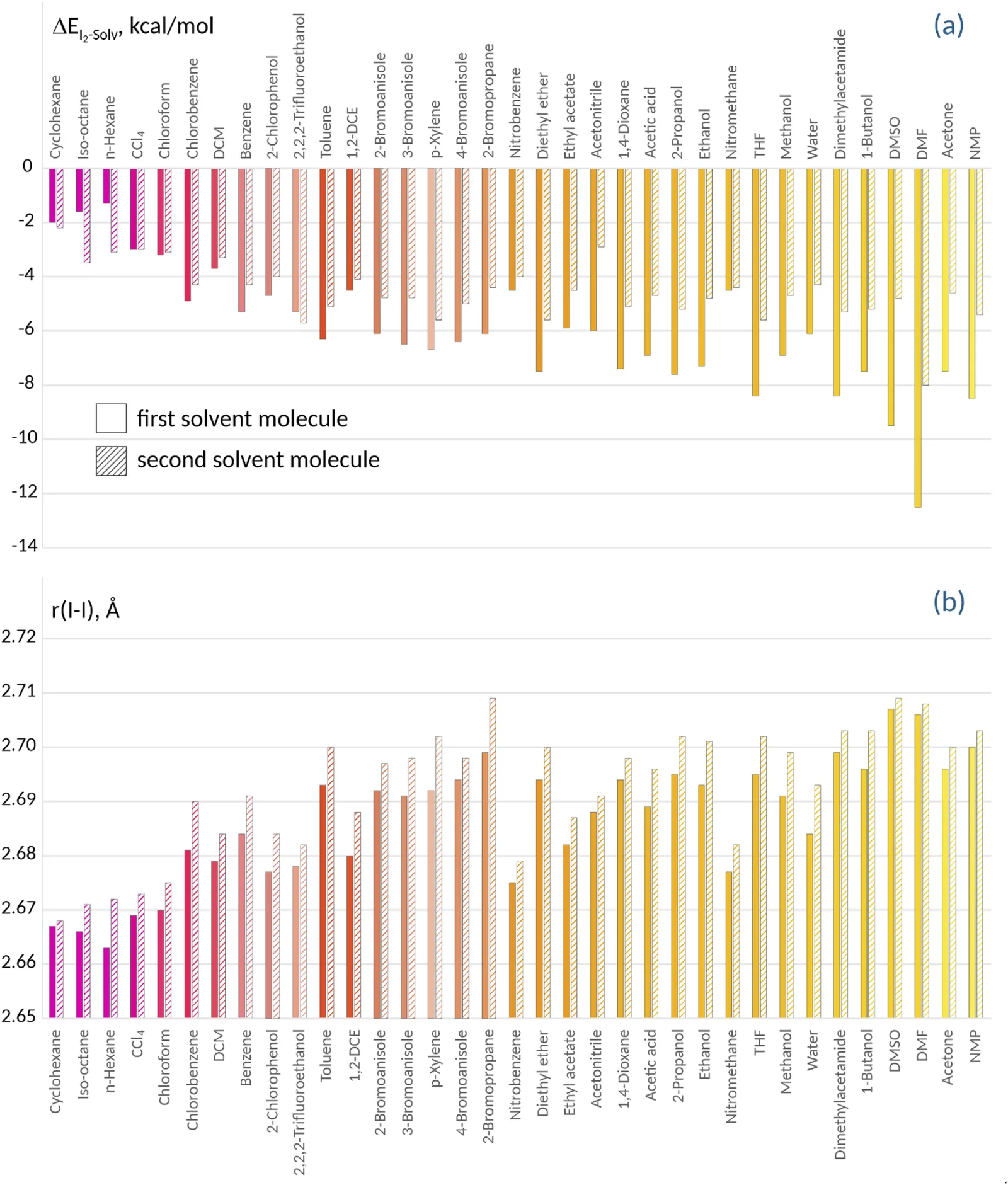
(a) Bonding energy of the I_2_ molecule with the first and second solvent molecules (Δ*E*_I2-Solv_, kcal mol^−1^); (b) I–I interatomic distance in the complex of the I_2_ molecule with one and two solvent molecules (*r*(I–I), Å); calculations were carried out using PBE0 D4 def2TZVP ZORA.

Notably, the formation of halogen bonds is accompanied by an increase in the I–I interatomic distance. For example, for cyclohexane, DCM, and methanol, the I–I interatomic distances calculated using the PBE0 D4 def2-TZVP ZORA method in a complex with one solvent molecule are 2.667, 2.679, and 2.691 Å, respectively, and 2.669, 2.675, and 2.687 Å using the ωB97X-2 def2-TZVP ZORA level of theory. The change in the I–I interatomic distance in the complex of the I_2_ molecule with one solvent molecule correlates with the color of the solution: for violet-colored solutions, the distance is minimal, whereas for yellow-colored solutions, the distance is maximal. Orange/red-colored solutions correspond to intermediate interatomic distances (Table 1 and Fig. 4b).

Coordination of the I_2_ molecule with one solvent molecule reduces the positive electrostatic potential in the σ-hole region of the I_2_ molecule on the opposite side from the coordination site (Fig. S3). The *φ* values are 0.129, 0.126, 0.118, and 0.096 a.u. for the isolated I_2_ molecule and for the I_2_ complexes with cyclohexane, dichloromethane, and methanol, respectively. This indicates that, after the coordination of the first solvent molecule, the probability of coordinating a second solvent molecule decreases, which agrees well with experimental data suggesting preferential solvation of only one of the two atoms in the I_2_ molecule.^71^ The decrease in the *φ* value of the iodine molecule following the coordination of the first solvent molecule shows a good correlation with the color of the solution across the entire range of solvents: for violet solutions, the decrease in *φ* is minimal, whereas for yellow solutions, the decrease is much greater (Fig. S4).

Complicating the microsolvation model involves creating a solvation shell consisting of several solvent molecules. In the case of cyclohexane, the model included 6 solvent molecules, whereas for DCM and MeOH, 10 solvent molecules were used. Simulating such microsolvation systems using DFT molecular dynamics with the PBE0/def2-SVP&LANL2DZ(I) method within a spherical potential (“nanoreactor”) allowed the calculation of the I–I interatomic distances averaged over 10^5^ points along the MD trajectory (100 ps with a step size of 1 fs). The average I–I distances also tended to increase when moving from cyclohexane to methanol (Fig. 3e–g).

Further development of the explicit solvation model for the I_2_ molecule involved simulating the condensed phase using periodic boundary conditions (PBC). Initially, calculations were performed for pure solvents (cyclohexane, dichloromethane, and methanol) to calculate the densities of these liquids. The accurate density values indicate a correct description of intermolecular interactions and allow further use of the chosen method for modeling of the I_2_ solutions. The cyclohexane model contained 30 solvent molecules in the periodic cell, whereas 40 solvent molecules were used for the DCM and MeOH models (Fig. S5). Following initial geometry optimization of the molecular systems for each solvent, molecular dynamics simulations were performed in the NPT-I ensemble, allowing cell size adjustments at a fixed temperature (298 K) and pressure (1 atm), using the PBE DZVP D3BJ method (see Computational Details in the SI). As a result, for each solvent, the volume of the cell stabilized after approximately 3 ps and began oscillating within a certain range. Therefore, to compute the density, the cell volume was averaged over the trajectory interval of 3.0–5.6 ps. The calculated densities of the pure liquids are in good agreement with the experimental data (Fig. S5b, d and f).

The NPT-I ensemble was also used for MD simulations of iodine solutions, where the model consisted of one iodine molecule and several dozen solvent molecules: 30 cyclohexane molecules, 40 dichloromethane molecules, and 40 methanol molecules. During the simulation, the volume of the periodic cell underwent equilibration, after which its magnitude began to fluctuate slightly around a certain value (Fig. 5). After the volume equilibration stage, MD simulations for each molecular system were continued to obtain a trajectory segment where the average I–I interatomic distance was calculated. The I–I distances for cyclohexane (2.765 Å) and dichloromethane (2.763 Å) media are close to each other, whereas for the methanol environment, this distance is significantly greater (2.823 Å), which is consistent with the results of MD simulations under microsolvation conditions in the “nanoreactor” (Fig. 3).

**Fig. 5:**
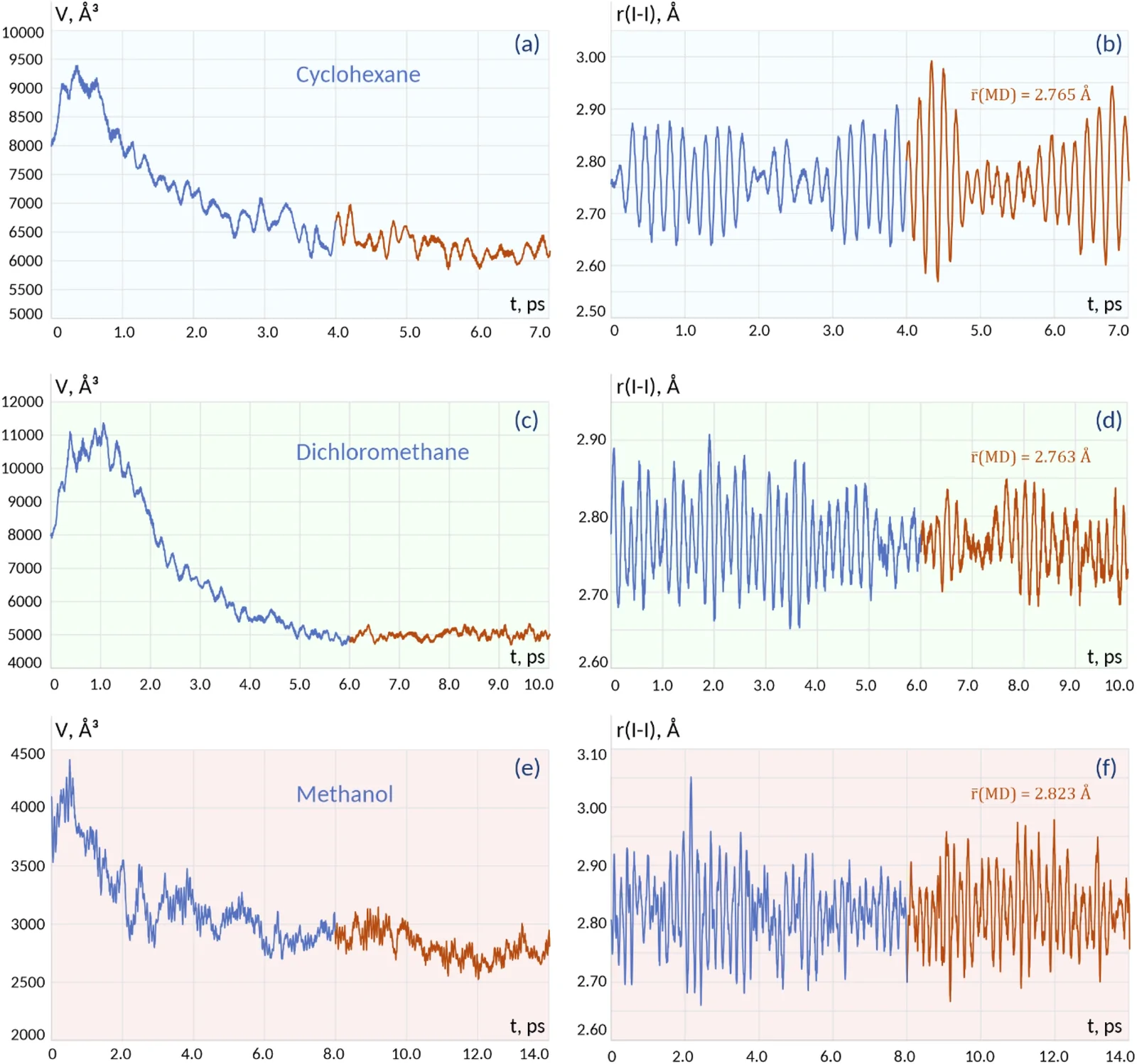
(a, c and e) Dependence of the periodic cell volume on time for a molecular system containing one I_2_ molecule and solvent molecules: 30 cyclohexane molecules, 40 dichloromethane molecules, and 40 methanol molecules, respectively; (b, d and f) dependence of the I–I interatomic distance on time for the corresponding I_2_ solutions, obtained from molecular system simulations in the NPT-I ensemble using the PBE DZVP D3BJ method. The segments of the trajectories for which the I–I interatomic distance was averaged are highlighted in orange.

Thus, the trends in the change in the I–I interatomic distance, obtained for solvate complexes with one or two solvent molecules, are reproduced in the condensed-phase model as well. That is, such a change in the I–I interatomic distance, which correlates reasonably well with the color of the solution, can indeed be attributed to the properties of the solvent medium.

### 2.2 Correlations between iodine solution color and the empirical parameters of solvent properties

The prospects of using the iSolv value as a new solvent property descriptor are confirmed by the numerous correlations found between the iSolv values and empirical solvent properties (Fig. 6 and 7).

**Fig. 6:**
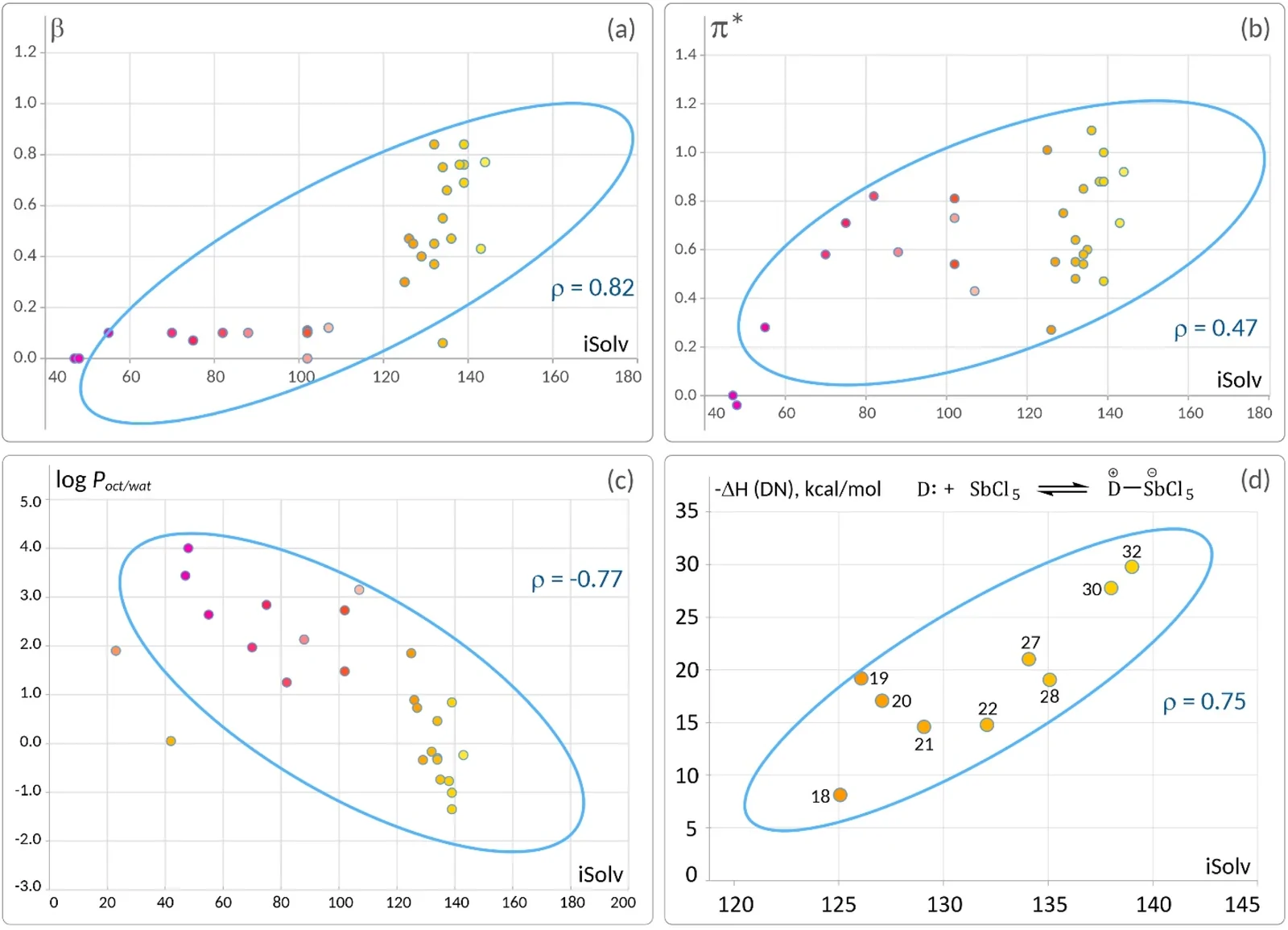
(a) Correlation between the hydrogen bond acceptance ability (*β*) of the organic solvents and the iSolv value of iodine solutions; (b) correlation between the polarity/polarizability parameter (π*) of the organic solvents and the iSolv value of iodine solutions (parameters *β* and π* are part of the Kamlet–Taft equation);^80^ (c) correlation between the organic solvent's partition coefficient log *P*_octanol/water_^78^ and the iSolv value of iodine solutions; (d) correlation between the solvent donor number (DN)^81^ and iSolv value of iodine solutions. The points are colored according to the solution colors. The Spearman correlation coefficient (*ρ*) and confidence ellipse (confidence level = 0.9) are provided.

**Fig. 7:**
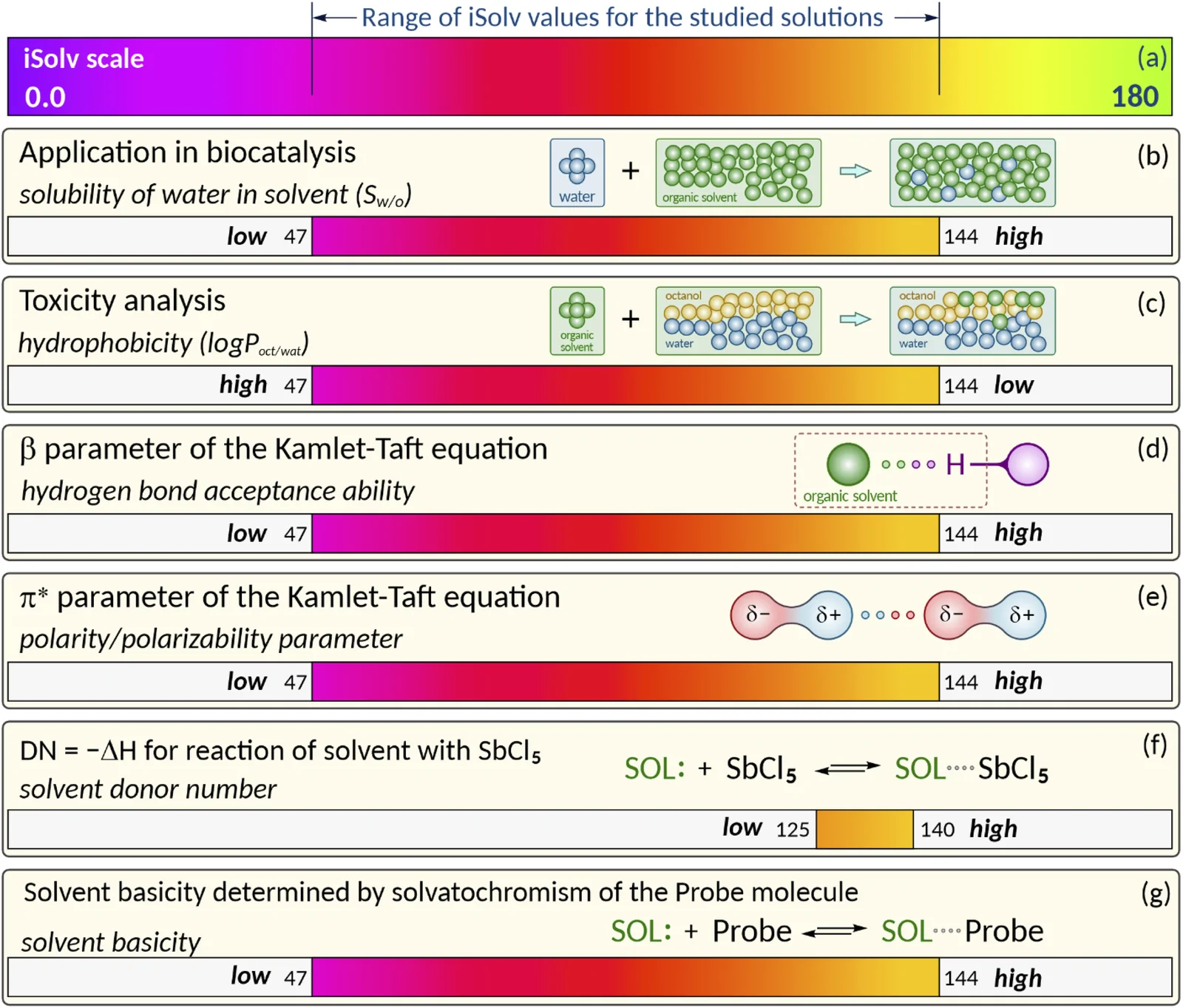
Line graph illustrating the applicability of the iSolv scale for certain properties of solvents (*S*_w/o_, log *P*, *β*, π*, DN, and solvent basicity): (a) the iSolv scale from 0 to 180° indicates the iSolv value range for the set of solvents studied in this work; (b) *S*_w/o_ descriptor (water solubility in an organic solvent), used in biocatalysis; (c) organic solvent partition coefficient log *P*_octanol/water_, used for toxicity analysis of the chemical compounds; (d) Kamlet–Taft hydrogen bond acceptance ability (*β*) of the organic solvents; (e) Kamlet–Taft polarity/polarizability parameter (π*) of the organic solvents; (f) DN: solvent donor number; (g) solvent basicity.

An important parameter used in biocatalysis is the water solubility in organic solvents. This parameter is relevant for biotechnological studies employing mixtures of water and organic solvents as media for enzymatic catalysis. For solvents with low iSolv values, poor water solubility is characteristic, and *vice versa* (Fig. 7b).^77^

One of the common characteristics of organic substances is the distribution coefficient log *P*, which characterizes the hydrophilic/hydrophobic properties of an organic substance, as this coefficient is determined by the ratio of equilibrium concentrations of the substance in the organic phase (octanol) and the inorganic phase (water). In this study, a pronounced correlation was found between the iSolv values of the iodine solutions and the log *P* values of the corresponding solvents (Fig. 6c and 7c). Iodine solutions of violet color are characterized by large positive log *P* values, *i.e.*, the corresponding solvents are hydrophobic substances, whereas yellow-colored solutions are grouped in the region of negative log *P* values, *i.e.*, the corresponding solvents are hydrophilic compounds, which is in good agreement with the experimental results. The Spearman correlation coefficient (*ρ*) for this correlation is −0.77, indicating a high degree of interrelationship between iSolv and log *P* (Fig. 6c).

To describe the influence of solvents on the course of chemical reactions, the Kamlet–Taft parameters *α*, *β*, and π* are often used, where *α* is the ability of the solvent molecule to act as a hydrogen bond donor, *i.e.*, to donate a proton; *β* is the solvent's hydrogen-bond-acceptor ability, *i.e.*, its ability to accept a hydrogen bond; and π* is a value that characterizes the polarity/polarizability of the solvent molecule and reflects the solvent's ability to stabilize charge.^79,80^ In this study, a pronounced correlation was found between the iSolv value and the *β* values (*ρ* = 0.82): violet solutions are characterized by small *β* values, and orange solutions have intermediate *β* values, while large *β* values are typical for yellow solutions (Fig. 6a and 7d). Since *β* reflects the ability of the solvent molecule to act as an electron pair donor, the high *β* values for yellow solutions are quite explainable: such solutions are formed in solvents containing oxygen and nitrogen atoms, which are active hydrogen bond acceptors. Moreover, the correlation between the π* value and the iSolv value is slightly less pronounced: *ρ* = 0.47 (Fig. 6b and 7e).

The clear correlation between the iSolv value and the solvent's donor number (DN) (*ρ* = 0.75 after excluding acetone from the set, Fig. 6d and 7f) also indicates good agreement between the iSolv value and the electron-donating properties of the solvent. The donor number is defined as the enthalpy (–Δ*H*) of the reaction between the studied molecule and SbCl_5_.^81^ The highest DN values correspond to yellow-colored solutions, whereas orange solutions are characterized by significantly lower DN values.

A similar characteristic of the electron-donating properties of a solvent is the enthalpy of the reaction between the solvent molecule and the BF_3_ molecule.^81^ In this case, the correlation is stronger than that for the reaction with SbCl_5_, and even without excluding acetone, *ρ* = 0.70, whereas excluding this solvent increases the coefficient to 0.79 (Fig. S6). In addition, iSolv correlates with the solvent basicity determined from the solvatochromic effect of a specially selected probe molecule (Fig. 7g).^37^

### 2.3 Predicting the color of iodine solutions *via* machine learning methods

In recent years, machine learning methods have gained increasing popularity in chemical research, accelerating the discovery of new compounds, investigating reaction mechanisms, and opening up new possibilities for optimizing chemical synthesis conditions and studying the condensed state of matter.^82–88^

In this work, a machine learning algorithm was developed that enables the approximate prediction of the color of iodine solutions for molecular solvents within the chemical domain represented by the training data. The predicted color can subsequently be used to classify the solvent into a specific solvating ability group, potentially expediting the optimization of organic synthesis conditions. For machine learning, the Hue value from the HSB color scale, which is commonly used in digital color representation, was selected. Converting the predicted Hue values to iSolv does not present difficulties.

To obtain an unbiased performance estimate, the dataset was split into a training set (35 solvents, Table 1) and an independent test set (10 solvents, Fig. S10, Tables S3 and S4) before any analysis. All feature normalisation parameters were computed on the training set only and then applied to the test set. Feature selection and hyperparameter tuning were performed exclusively on the training set using leave-one-out cross-validation; the test set was not involved in any of these steps.

Three machine learning models were trained: linear regression,^89^ random forest (“scikit-learn” library),^90^ and gradient boosting (CatBoost library)^91^ with a leave-one-out validation strategy (Fig. S8, S9 and Table S2).^92^ The dataset used consisted of the data presented in Tables 1 and S1. An optimal feature set search was conducted *via* a “greedy search” method for two datasets. The first dataset includes the Δ*E*_GAP-1_, Δ*E*_I2-Solv1_, *r*_1_(I–I) and *φ*_1_, (Feature set 1, see Table 1), whereas the second dataset, along with the calculated parameters, also includes the physical properties of the solvents – *ε*_r_, *µ*, and *n*^25^ (Feature set 2, Fig. 8 and Tables S1 and S2). Hue angles were transformed from the [0; 180] scale to the [–90; 90] scale to center the distribution. All the data were normalized using the “normalize” function from the “sklearn” package.^93^

**Fig. 8:**
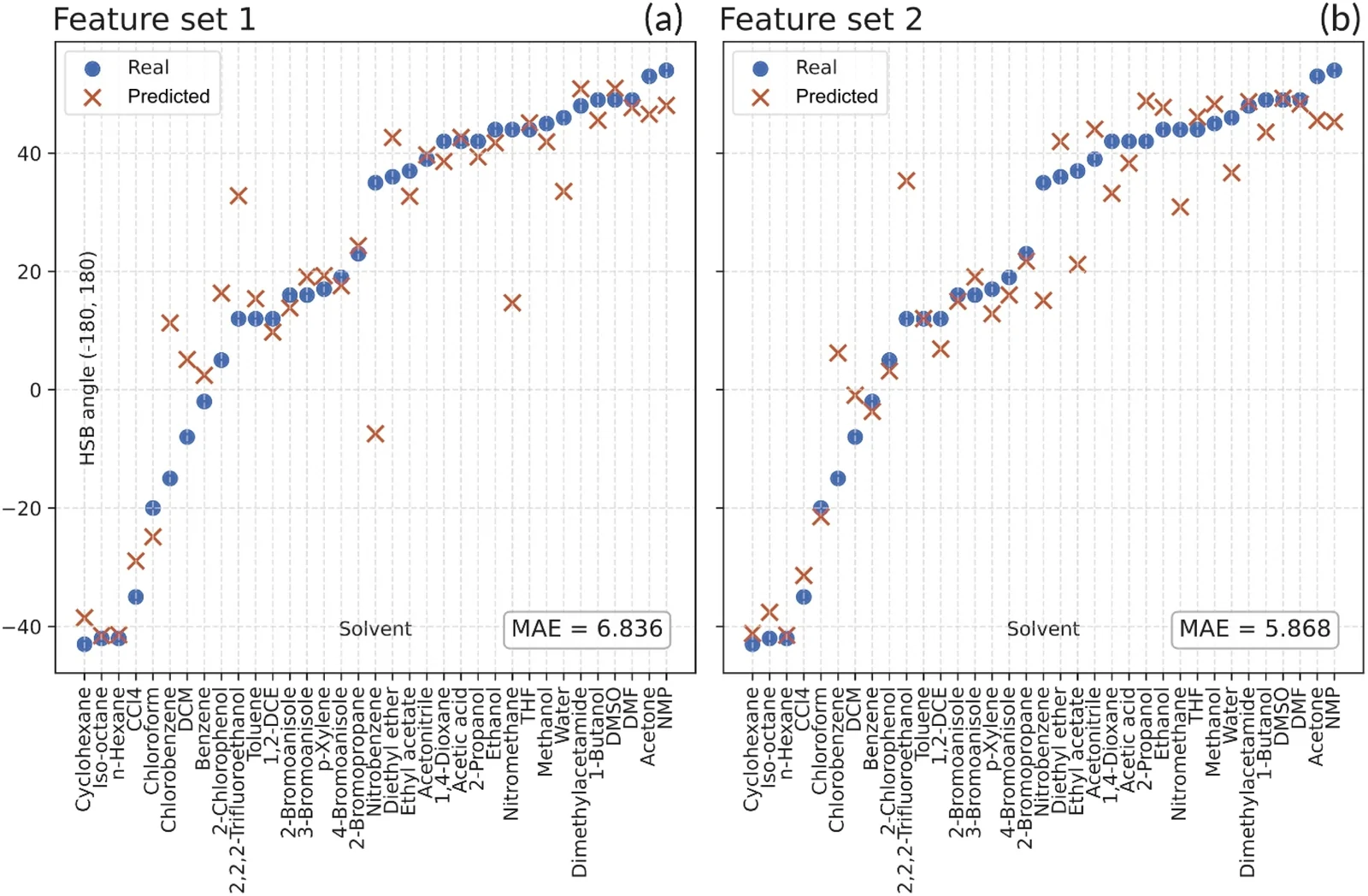
Comparison of the actual Hue values of iodine solutions for a series of solvents with the Hue values predicted by the “gradient boosting” algorithm using Feature set 1 (a) and the “gradient boosting” algorithm using Feature set 2 (b). For each algorithm, the minimum mean absolute error (MAE) in predicting the Hue angle is provided (Table S2).

For each algorithm, sets of parameters that lead to the smallest errors were identified (Optimal Dataset in Table S2). The final models were then retrained on the full training set and evaluated once on the test set (Table S6). The best results were achieved by the “gradient boosting” algorithm using Feature set 2 and the “random forest” algorithm using Feature set 2 (Fig. 8 and Table S2; see Fig. S8 and S9 for comparisons of algorithms with different feature sets).

On the independent 10-solvent test set, the best model (gradient boosting, Feature set 2) achieved an MAE of 8.8 iSolv units relative to manually determined values and 10.1 units relative to automatically determined values (Tables S5 and S6). This external test supports generalization beyond the training data.

### 2.4 Practical application of color analysis of iodine solutions for solvent effect analysis

A potential practical application of iSolv is the fast and approximate prediction of the solvation ability of a solvent. This usage of iSolv is justified by the numerous correlations found in this study between the color of the iodine solution and not only with the properties of solvents (see Section 2.2), but also with the parameters of the chemical reactions occurring in these solvents (Fig. 9). Specifically, solvents that result in a yellow color of the solution result in higher rates of the Menshutkin reaction (the process of interaction between Et_3_N and EtI), Wagner–Meerwein rearrangement, aromatic nucleophilic substitution, electrophilic substitution, [2 + 2] cycloaddition,^6^ and Heck reaction (Fig. 9b–f, h respectively and S7).^36^ On the other hand, some free radical addition processes proceed faster in solvents leading to violet-colored solutions, whereas in solvents from the yellow region, such processes occur more slowly (Fig. 9g).^6^ In this case, the dipole moment of the initial thiyl radical is greater than that of the activated complex. Consequently, the reactant is stabilized by solvation to a greater extent than the transition state of the reaction. That is, the transition from a nonpolar to a polar medium leads to stabilization of the reactant rather than the transition state. As a result, the reaction rate decreases in a polar medium.^94^

**Fig. 9:**
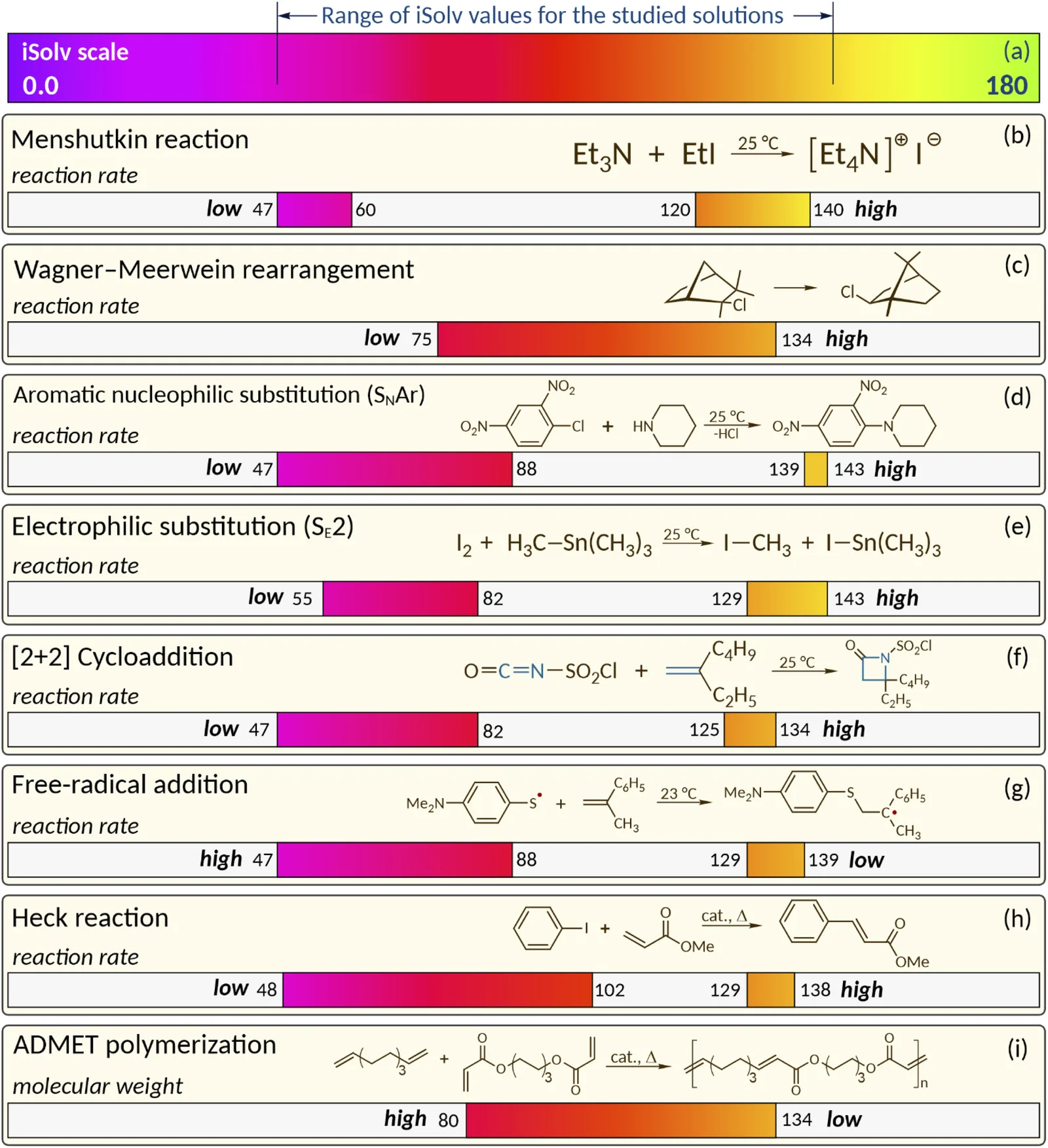
Line graph illustrating the applicability of the iSolv scale for chemical processes in various organic solvents: (a) the iSolv scale from 0 to 180° indicates the iSolv value range for the set of solvents studied in this work; (b–h) rates of different chemical reactions: the Menshutkin reaction (see Fig. S7), Wagner–Meerwein rearrangement, aromatic nucleophilic substitution, electrophilic substitution, [2 + 2] cycloaddition, free-radical addition and Heck reaction, respectively; (i) the molecular weight of the polymer obtained as a result of ADMET polymerization.

An interesting correlation has been found for the process of acyclic diene metathesis (ADMET) copolymerization of dienes and diacrylates: solvents with low iSolv values lead to high-molecular-weight polymers, whereas solvents with high iSolv values result in low molecular weight polymers (Fig. 9i).^95^

For practical purposes, we developed a colored iSolv scale consisting of four regions, each corresponding to the degree of solvating ability (Fig. 1, S11f and S12 for high-resolution image of the scale suitable for both on-screen comparison and printing). Although iSolv has numerical values, there are no clear boundaries between the levels of solvating ability. For rapid visual categorization, the provisional operational intervals shown in Fig. S12b are as follows: low, 0 ≤ iSolv < 70; moderate, 70 ≤ iSolv < 110; high, 110 ≤ iSolv < 130; and very high, 130 ≤ iSolv ≤ 180. These thresholds are heuristic color-scale boundaries rather than statistically optimized values. Quantitative iSolv values become necessary when a series of solvents is available and correlations between the properties of a chemical system and the properties of solvents are being sought. To use this scale, it is necessary to prepare an iodine solution in the solvent under investigation at a specified concentration: approximately 2.5 mg of crystalline iodine is dissolved in 10 ml of the solvent (∼10^−3^ mol L^−1^). After thorough mixing and formation of a uniform color, the color of the solution is compared with the colors on the color scale to find a match and assign it to a particular group. For comparison, any transparent vial with an optical path length of approximately 5–20 mm can be used, provided the specified concentration is maintained. The photographs in this article and the SI (Fig. 1f, g, S11b–f and S13c–e) depict solutions at this standard concentration prepared in standard NMR tubes (see the SI for detailed preparation notes). Additionally, a SI tutorial demonstrates the color comparison procedure using solutions in 2-mL clear vials (∼10^−3^ mol L^−1^) alongside the iSolv scale.

The application of iSolv may be hindered when iodine is insufficiently soluble, reacts with the medium, or does not produce a stable color. In the present study, the formamide sample did not yield a color suitable for quantitative assignment and was therefore excluded from the dataset (Fig. 2b). Solvent mixtures can also be ambiguous because the color response may reflect preferential or competing interactions with several components. More generally, iSolv is an empirical descriptor rather than a universal replacement for specific solvent scales, and its correlations with chemical outcomes can be weak depending on the process.

Since human perception of color is subjective, assigning a solvent to a particular group on the basis of the color of the iodine solution can lead to ambiguity. To assess the extent of the “human factor”, a group of 36 individuals was asked to categorize 50 solutions into four categories corresponding to different levels of solvating ability, using the set of solutions prepared for the color classification study (Fig. S19 and S20). The collected data indicate high reproducibility in human color perception. The greatest number of differences in solution distribution occurred in the “borderline” area between solvents with low solvating ability and those with moderate solvating ability.

To conveniently and quickly determine the solvent properties and calculate the iSolv value from a photograph without requiring ideal shooting conditions, a Python script with a user-friendly web interface was developed for this study (see Section 5 of the SI, Fig. S13–S18). This software calculates the iSolv value of the analyzed iodine solution in the studied solvent based on iSolv values from reference solutions with known iSolv values. Photos of the analyzed solution and reference solutions can be taken using a smartphone camera without any additional color correction. After uploading three photographs (two reference photographs and a photograph of the analyzed solution), the program calculates corrections using two methods, based on comparing the experimentally obtained iSolv value with the reference iSolv values for the reference solutions (Fig. 2). These corrections are then used to adjust the iSolv value of the analyzed solution. This software allows one to quickly and easily determine the iSolv value for any solution (see Tables S7–S10 for iSolv test calculations).

## 3 Conclusions

In this study, we demonstrated that the color of diluted iodine solutions can be used as a practical and informative descriptor of solvent solvating ability. By combining experimental color measurements with quantum chemical calculations, we established quantitative correlations between solution color and fundamental solvent properties, including bonding energy with iodine, electronic parameters and microsolvation characteristics. The resulting color-based metric, designated as iSolv, was introduced as a descriptor capable of capturing the collective solvation behavior of solvents.

The iSolv descriptor is rooted in fundamental molecular interactions: solvents forming stronger intermolecular complexes with iodine result in specific color shifts, which correlate with parameters of the intermolecular complexes, solvent donor number, log *P*, and even reaction rates across a variety of organic transformations. This unique interrelation between solution color and electronic interaction strength demonstrates that iSolv inherently reflects both the energetic and structural features of intermolecular solvation phenomena.

A major strength of the iSolv approach is its exceptional simplicity, affordability, and accessibility. It requires only minimal laboratory resources – iodine, a solvent sample, and simple visual or digital color comparison – making it suitable for rapid, preliminary, or educational assessments of solvent behavior. Its ability to provide a fast, visual estimation of solvating ability offers clear advantages over traditional multi-parameter solvent scales, which typically require extensive computational or spectroscopic data.

In this study, a fundamental observation – the color diversity of iodine solutions – was transformed into a quantitative and experimentally reliable solvent descriptor. This concept bridges fundamental physical chemistry with practical laboratory use, enabling broader adoption of solvatochromic principles for solvent selection, reaction optimization, and even machine-learning-based property prediction.

Despite its potential, iSolv should be regarded as an experimental descriptor rather than a universal replacement for specific solvent scales. Its application is limited when iodine is poorly soluble, reacts with the medium, or gives an unstable color, and interpretation of solvent mixtures may be non-unique. Correlations of iSolv with solvation ability are process-dependent and may be weak. The independent machine-learning test set is also small and does not establish reliability for media outside the represented molecular-solvent domain. Future work should expand the solvent database, refine standardized digital color acquisition, and evaluate predictive models across broader chemical domains.

Overall, this study establishes iodine-based solvatochromism not just as a visual observation, but as a scientifically grounded, quantitative, and readily applicable descriptor (iSolv) with the potential to enrich both the fundamental understanding and practical application of solvent effects in chemistry.

## Software

For testing purposes, the web service for calculating the iSolv descriptor is available at: https://isolv.ananikovlab.ai

Instructions about the software are provided in the SI. Use of the software is not mandatory; the iSolv descriptor can be calculated manually as described above.

## Author contributions

E. G. G.: manuscript preparation, quantum-chemical modeling, data analysis, software testing; L. A. A. and D. M. A.: experiment, software testing, data analysis, manuscript preparation; A. A. O.: development of the machine learning algorithm, manuscript preparation, data analysis; K. V. V.: discussion of machine learning algorithms, initial assessment of their applicability to the project; V. P. A.: idea development, conceptualization, project supervision, software development, manuscript preparation. All authors discussed and approved the final version of the manuscript.

## Conflicts of interest

The authors declare no competing financial interests.

## Supplementary Material

SC-017-D6SC03325C-s001

SC-017-D6SC03325C-s002

## Data Availability

The data for this study are described in the article and supplementary information (SI). Supplementary information: electronic parameters of solvent molecules, intermolecular complexes between the iodine molecule and solvent molecules, additional molecular dynamics data, correlation between the iSolv value and empirical parameters of solvent properties, machine learning results and user guide of the software for the iSolv prediction, methodology of practical usage of the iSolv descriptor, reproducibility test, XYZ coordinates of the optimized molecular structures (Table S22) and also a tutorial video on the reported procedure. See DOI: https://doi.org/10.1039/d6sc03325c.
