# Supplementary material for: A unified solvatochromic–computational–machine–learning approach for solvent property prediction based on iodine color response
Source: Chem Sci. 2026 Aug 24;17(39):18933–50. doi: 10.1039/d6sc03325c (PMC13502256; doi:10.1039/d6sc03325c)
Supplement: Supplementary file 1 [file SC-017-D6SC03325C-s001.pdf]

Supplementary Information (SI) for Chemical Science.
This journal is © The Royal Society of Chemistry 2026

Supporting Information

for the article

A unified solvatochromic–computational–machine–learning approach for solvent property prediction based on iodine color response

Evgeniy G. Gordeev, Liana A. Arakelyan, Daria M. Arkhipova, Alexander A. Oganov, Kirill V. Vokhmintsev, Valentine P. Ananikov*

N. D. Zelinsky Institute of Organic Chemistry, Russian Academy of Sciences, 119991, Leninsky prospect, 47, Moscow, Russia; http://AnanikovLab.ru

val@ioc.ac.ru

Content

1. Correlation between iSolv and electronic parameters of solvent molecules 2

2. Correlation between iSolv value and empirical parameters of solvent properties 7

3. Prediction of iodine solution color by machine learning methods 8

4. Practical use of the method for determining iSolv value and solvent solvating ability based on the iodine solution color 13

5. In-house developed software «iSolv Calculator» for Hue and iSolv values calculation: step-by-step user guide 14

6. Reproducibility of the color measurements 23

7. Computational Details 30

8. Materials and methods 30

9. XYZ coordinates and energies of the optimized molecular structures 32

10. REFERENCES 58

1. Correlation between iSolv and electronic parameters of solvent molecules

Table S1. The values of the relative permittivity (ε_r), dipole moment (μ, D) and refractive index (n^{25}) for different solvents.[1] The Hexadecimal, RGB, HSB, and iSolv values of the iodine solution are given for each solvent.

	Hex	RGB	HSB	iSolv	Solvent	ε_r	μ	n^{25}
1	#F300AD	243-0-173	317-100-95	47	Cyclohexane	2.0	0.3	1.424
2	#FF00B4	255-0-180	318-100-100	48	Iso-octane	2.2	0.0	1.389
3	#FD00B2	253-0-178	318-100-99	48	*n*-Hexane	1.9	0.0	1.372
4	#FF0096	255-0-150	325-100-100	55	CCl_4	2.2	0.0	1.459
5	#FF2D72	255-45-114	340-82-100	70	Chloroform	4.8	1.1	1.444
6	#FF225A	255-34-90	345-87-100	75	Chlorobenzene	5.6	1.3	1.523
7	#FF445D	255-68-93	352-73-100	82	DCM	9.1	1.8	1.421
8	#FF8287	255-130-135	358-49-100	88	Benzene	2.3	0.0	1.498
9	#EE847B	238-132-123	5-48-93	95	2-Chlorophenol	7.4 [2]	1.15 [3]	1.552 [2]
10	#FF9F86	255-159-134	12-47-100	102	2,2,2-Trifluoroethanol	27.0 [4]	2.5 [5]	1.291 [6]
11	#FF4A1E	255-74-30	12-88-100	102	Toluene	2.4	0.4	1.494
12	#FF5326	255-83-38	12-85-100	102	1,2-DCE	10.5	1.8	1.444
13	#F48057	244-128-87	16-64-96	106	2-Bromoanisole	8.96	2.64 [7]	1.573 [2]
14	#F48C67	244-140-103	16-58-96	106	3-Bromoanisole	7.0 [8]	3.10 [7]	1.564 [2]
15	#FFB99D	255-185-157	17-38-100	107	*p*-Xylene	2.3	0.0	1.496 [6]
16	#F89263	248-146-99	19-60-97	109	4-Bromoanisole	7.4	2.74 [7]	1.564 [2]
17	#FE9451	254-148-81	23-68-100	113	2-Bromopropane	9.5	2.2	1.429 [6]
18	#FF9600	255-150-0	35-100-100	125	Nitrobenzene	34.8	4.0	1.550
19	#FF9700	255-151-0	36-100-100	126	Diethyl ether	4.3	1.3	1.352
20	#FF9E00	255-158-0	37-100-100	127	Ethyl acetate	6.0	1.7	1.370
21	#FFA700	255-167-0	39-100-100	129	Acetonitrile	37.5	3.2	1.342
22	#FFB100	255-177-0	42-100-100	132	1,4-Dioxane	2.2	0.4	1.420
23	#FFB301	255-179-1	42-100-100	132	Acetic acid	6.2	1.7	1.370
24	#FFB300	255-179-0	42-100-100	132	2-Propanol	18.3	1.7	1.375
25	#FFBB00	255-187-0	44-100-100	134	Ethanol	22.4	1.7	1.359
26	#FFBA00	255-186-0	44-100-100	134	Nitromethane	37.3 [6]	3.46 [6]	1.380 [6]
27	#FFB900	255-185-0	44-100-100	134	THF	7.6	1.75	1.404
28	#FFBE00	255-190-0	45-100-100	135	Methanol	32.6	1.7	1.326
29	#FFC506	255-197-6	46-98-100	136	Water	79.7	1.9	1.332
30	#FFCE00	255-206-0	48-100-100	138	Dimethylacetamide	37.8	3.8	1.436
31	#FFD100	255-209-0	49-100-100	139	1-Butanol	18.2	1.66	1.397
32	#FFD100	255-209-0	49-100-100	139	DMSO	46.6	3.96	1.476
33	#FFD200	255-210-0	49-100-100	139	DMF	36.7	3.8	1.427
34	#FFE733	255-231-51	53-80-100	143	Acetone	20.6	2.9	1.357
35	#FFEC49	255-236-73	54-71-100	144	NMP	32.2 [9]	4.1 [10]	1.467 [11]

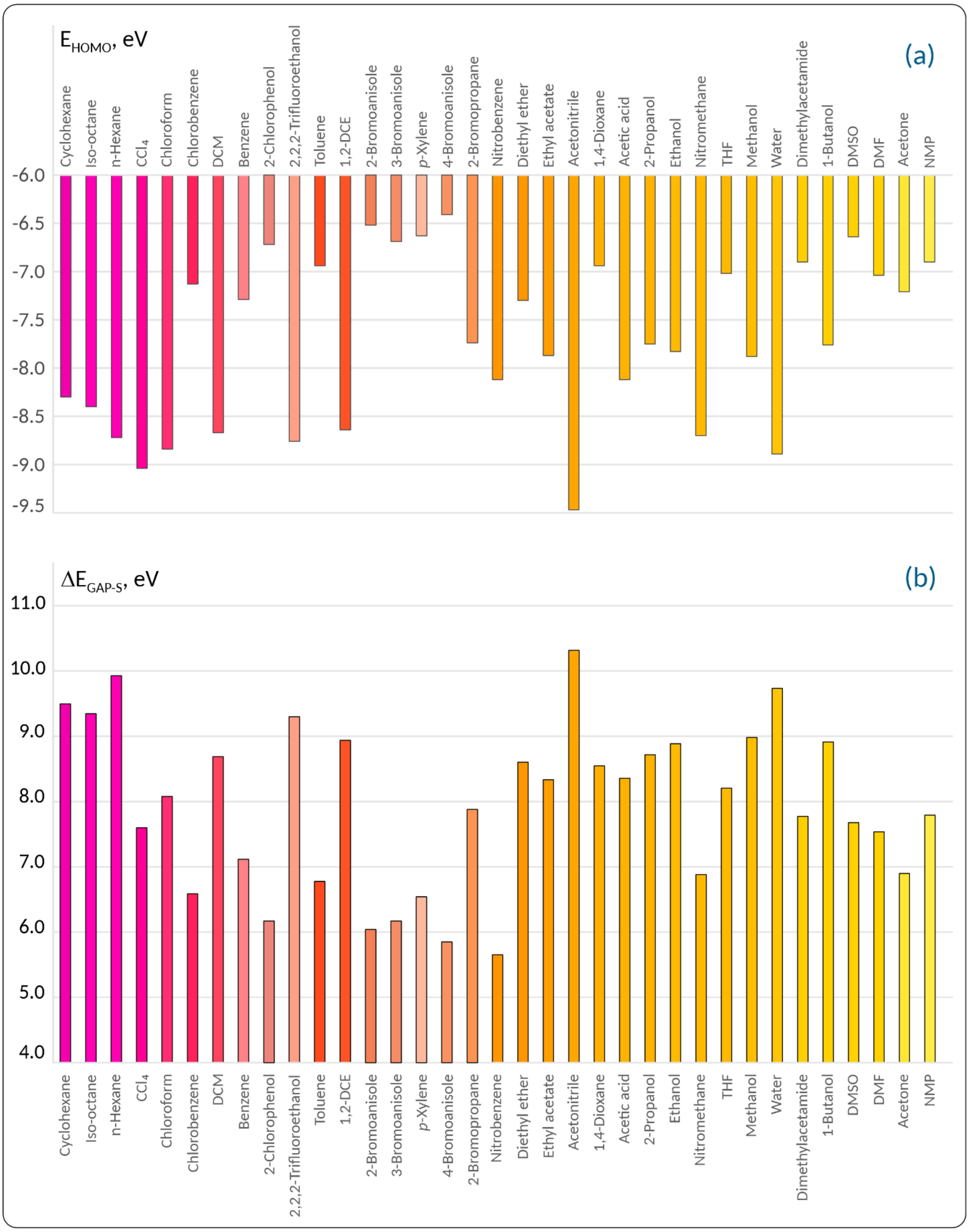

Figure S1. (a) Values of the HOMO energy (E_{HOMO}, eV) for isolated solvent molecules; (b) energy difference |LUMO-HOMO| ($\Delta E_{GAP\text{-}S}$, eV) for the one isolated solvent molecule; PBE0 D4 def2TZVP ZORA.

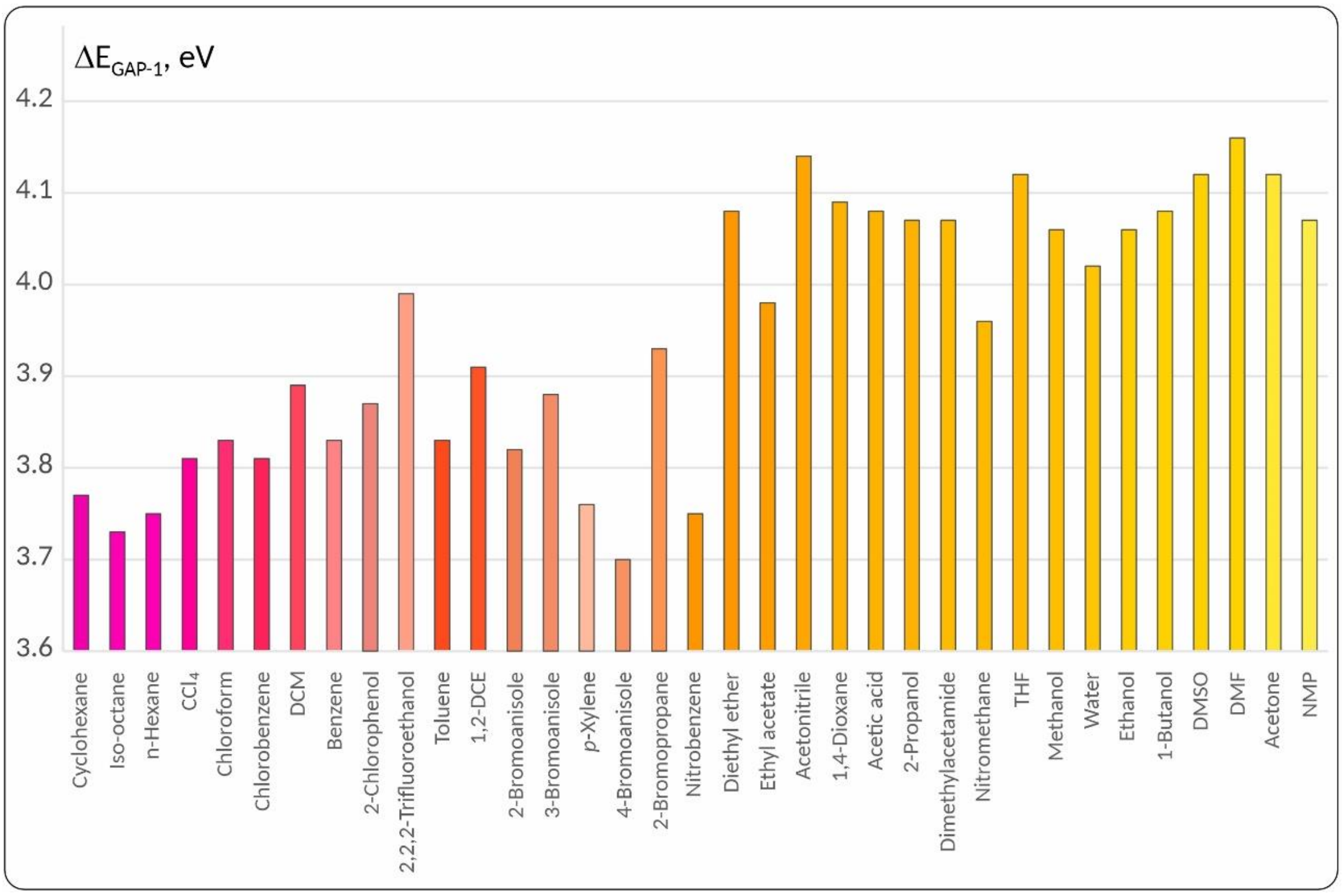

Figure S2. Energy difference |LUMO-HOMO| ($\Delta E_{GAP\text{-}1}$, eV) for the complex of one solvent molecule with one I_2 molecule; PBE0 D4 def2TZVP ZORA.

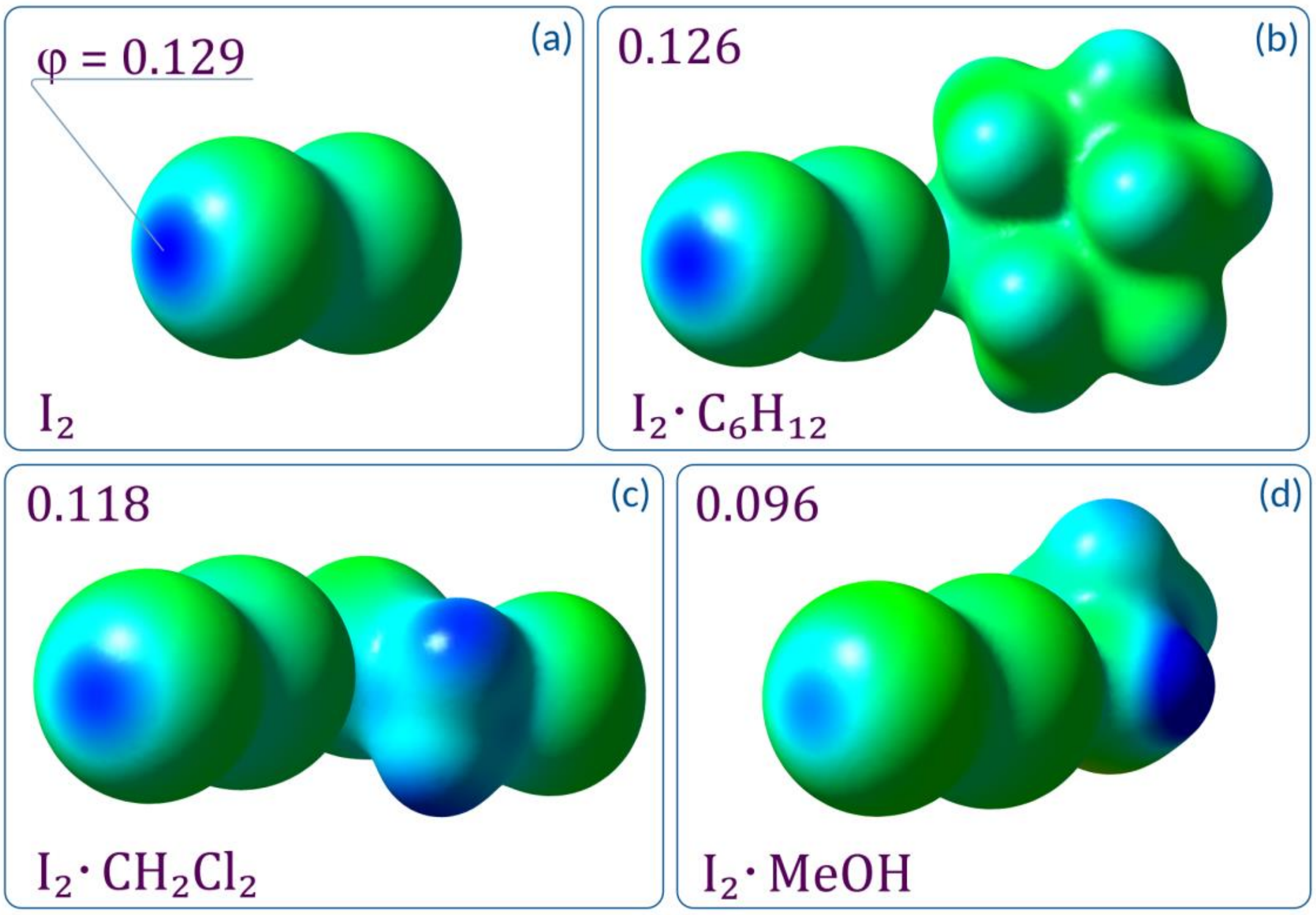

Figure S3. Molecular electrostatic potential (MEP) for isolated I_2 molecule (a) and intermolecular complexes of the I_2 molecule with one solvent molecule: (b) $I_2{\cdot}C_6H_{12}$; (c) $I_2{\cdot}CH_2Cl_2$; (d) $I_2{\cdot}$MeOH. The maximum positive values of the electrostatic potential (φ) in the σ-hole region are given.

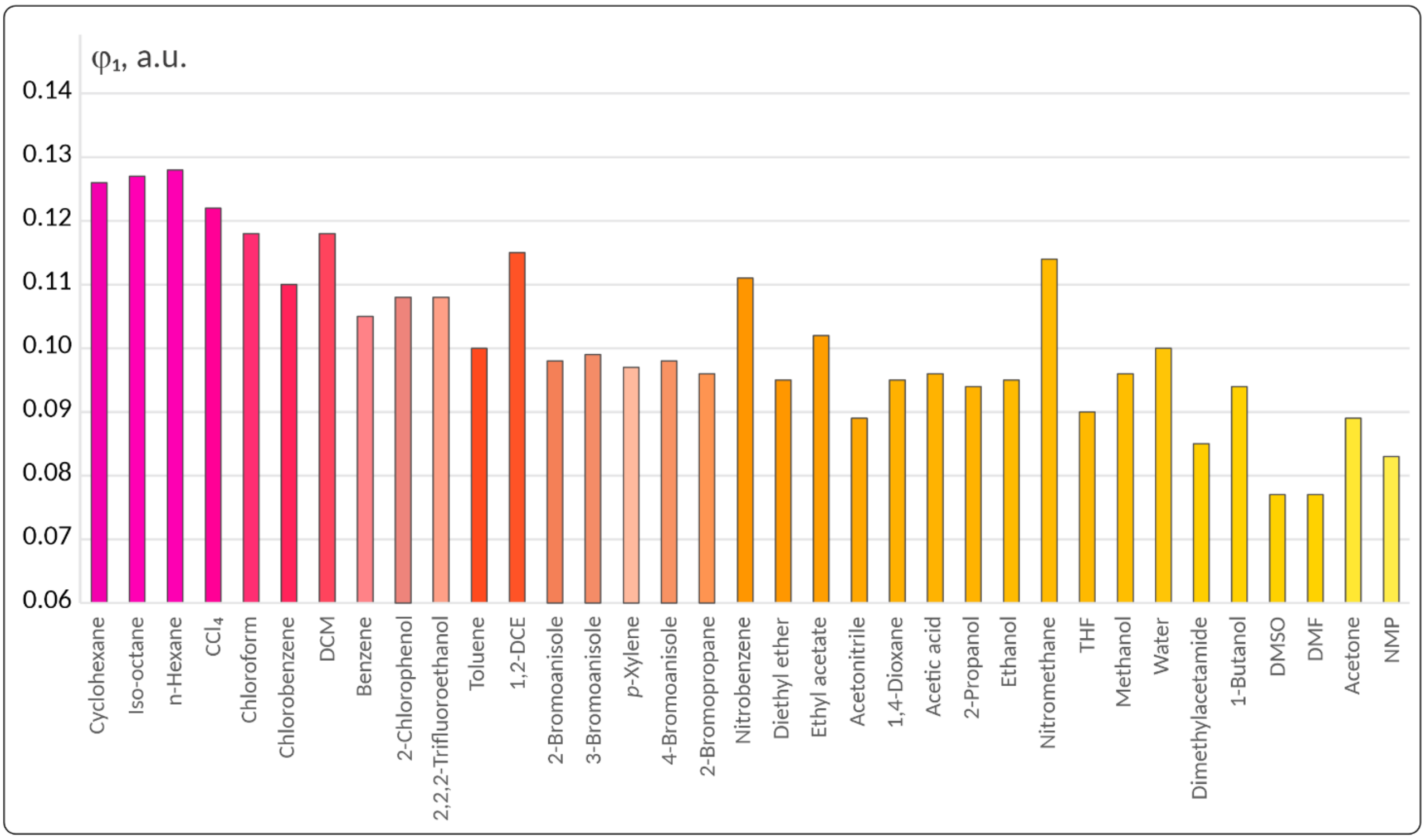

Figure S4. ESP values in the region of the solvent-free σ-hole of the I_2 molecule in complex with one solvent molecule; PBE0 D4 def2TZVP ZORA.

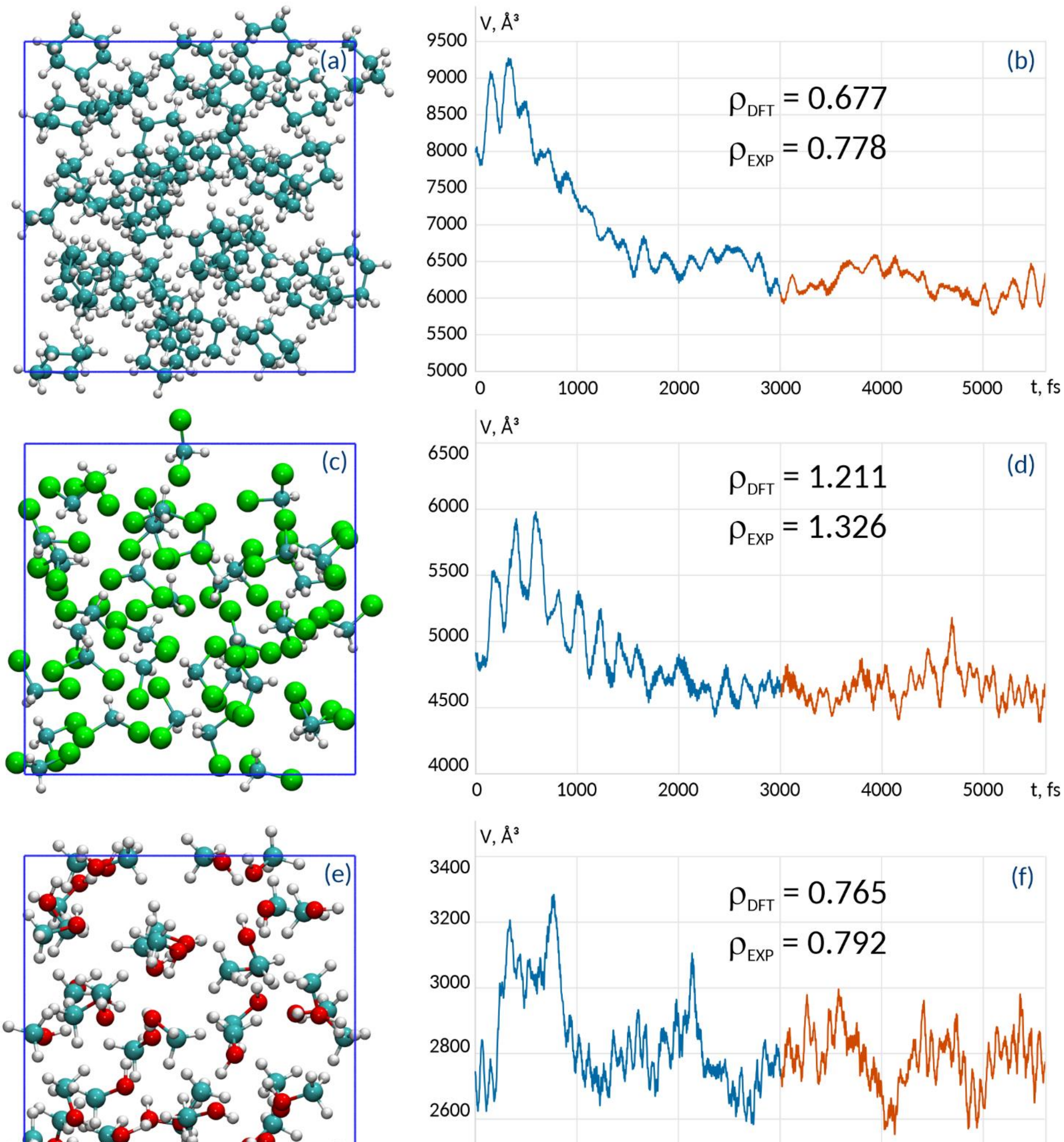

Figure S5. (a, c, e) Molecular models of pure solvents: 30 molecules of cyclohexane, 40 molecules of dichloromethane, and 40 molecules of methanol, respectively. A periodic cell is shown for each solvent; (b, d, f) periodic cell volume versus time dependences for cyclohexane, dichloromethane, and methanol, respectively, obtained from modeling the solvents in the NPT-I ensemble by the PBE DZVP D3BJ method. The region of the trajectory where the averaging of the cell volume was performed is highlighted in orange, and the DFT-calculated and experimental density values ($g \cdot cm^{-3}$) are given for each solvent.

2. Correlation between iSolv value and empirical parameters of solvent properties

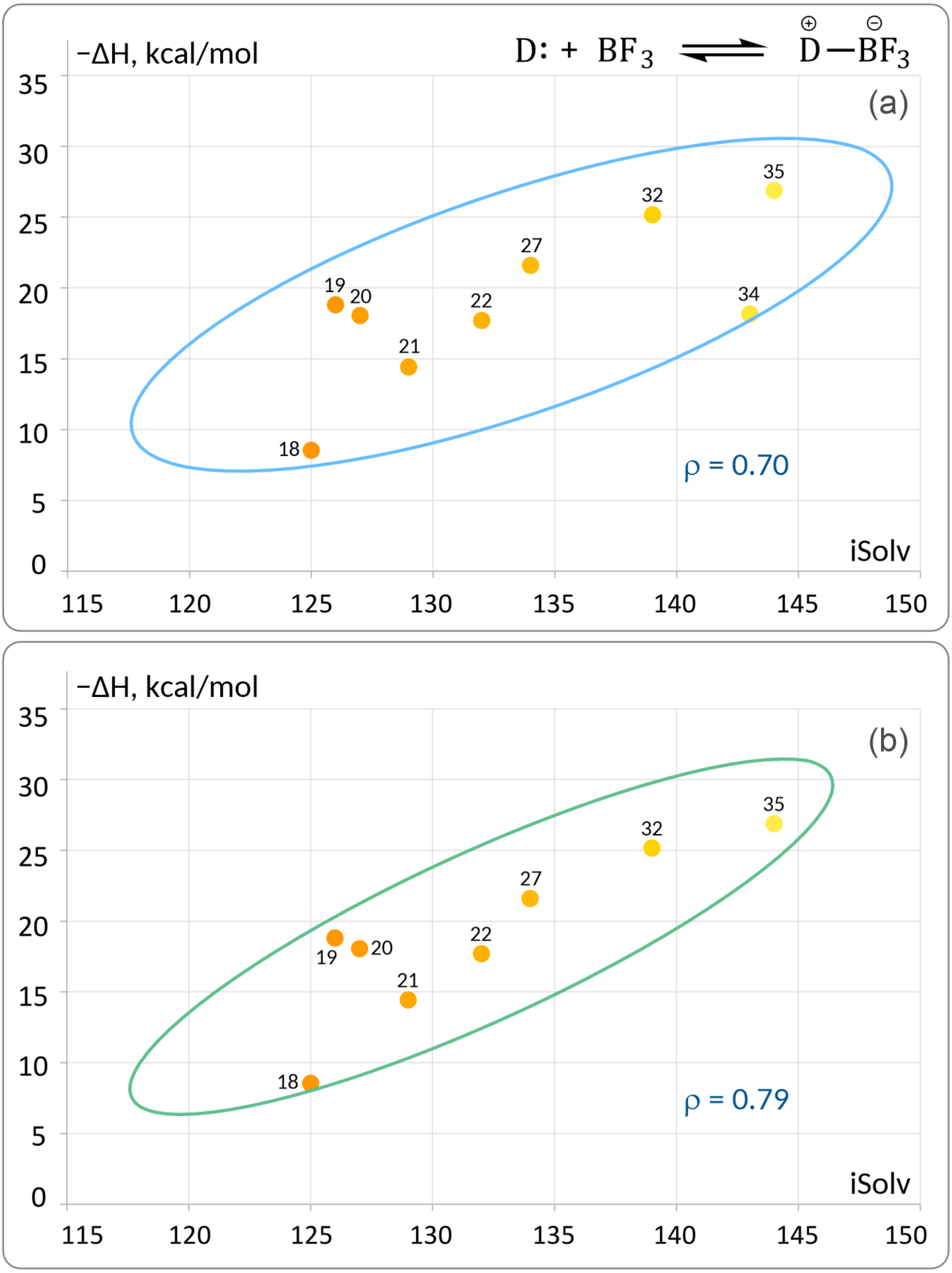

Figure S6. Correlation between BF_3 affinity of organic solvents[12] and iSolv value of iodine solutions: (a) the set of solvents includes acetone (34); (b) the set of solvents excludes acetone. The Spearman correlation coefficient (ρ) and the confidence ellipse (confidence level = 0.9) are provided.

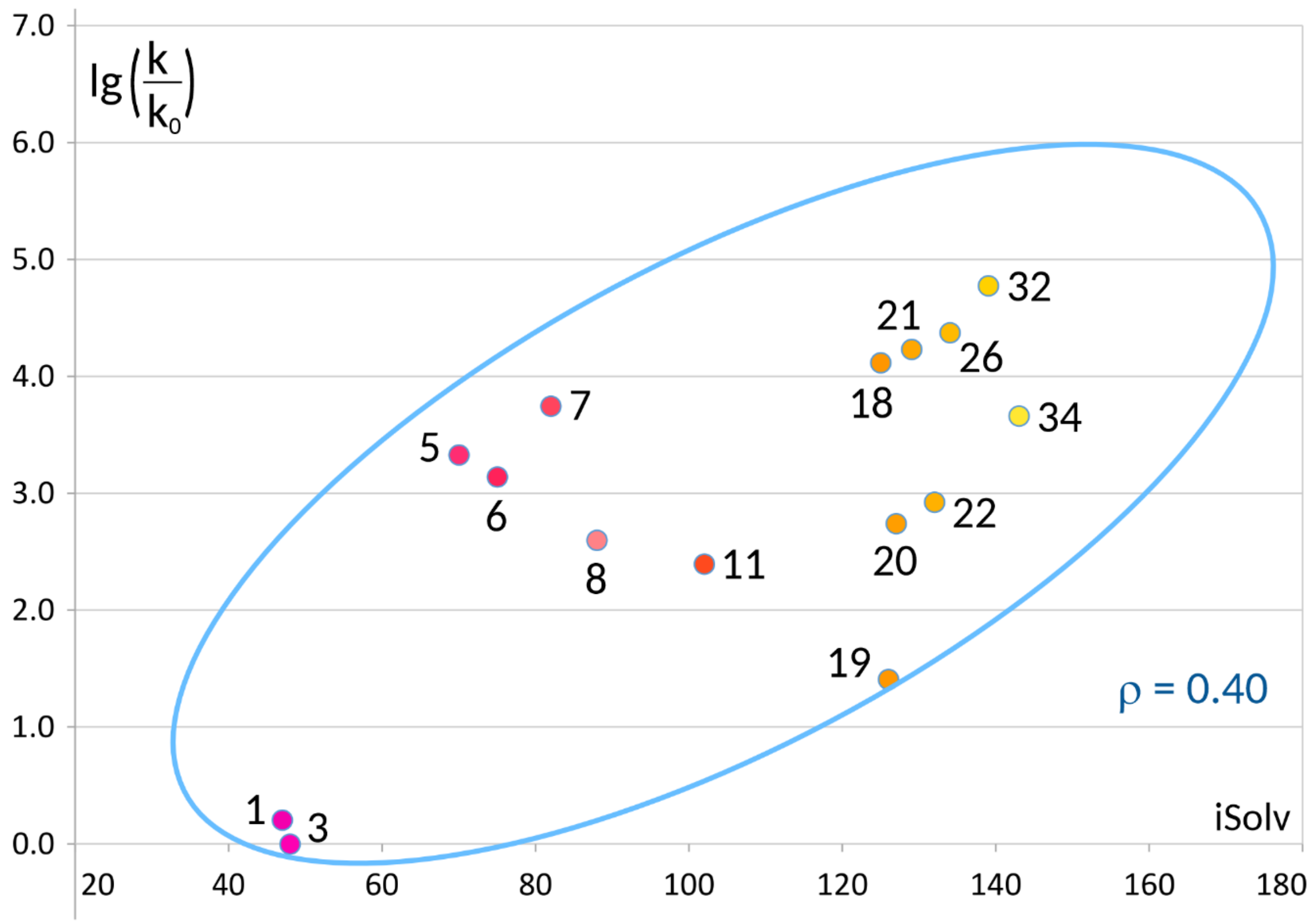

Figure S7. Correlation between lg(k/k$_0$) for the Menshutkin reaction (reaction of Et_3N with EtI) in different solvents[13] and iSolv value of iodine solutions. The Spearman correlation coefficient (ρ) and the confidence ellipse (confidence level = 0.9) are provided.

3. Prediction of iodine solution color by machine learning methods

Validation protocol

Before model development, the data were divided into a 35-solvent training set (Table 1 of the main text and Table S1) and a 10-solvent independent test set (Tables S3 and S4). The independent test set was not used for feature selection, normalization, or hyperparameter tuning. These steps were performed only on the training set using leave-one-out cross-validation. Final models were retrained on the complete training set and evaluated once on the independent test set (Tables S5 and S6). Because both sets are small and contain conventional molecular solvents, the external test supports generalization.

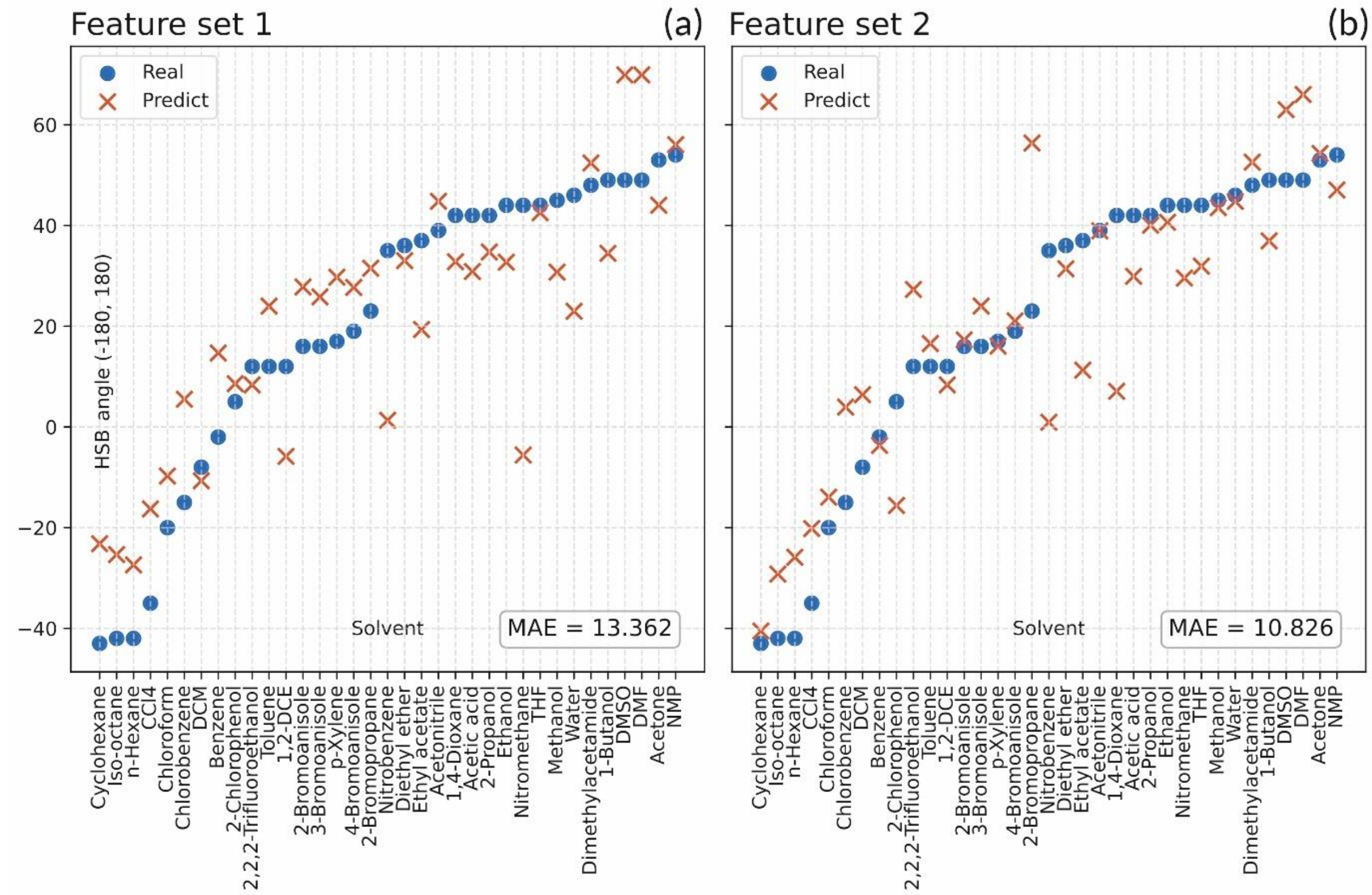

Figure S8. Comparison of the actual Hue values of iodine solutions for a series of solvents with the Hue values predicted by the "Linear Regression" algorithm using Feature set 1 (a) and Feature set 2 (b). For each algorithm, the minimum mean absolute error (MAE) in predicting the Hue angle is provided (Table S2).

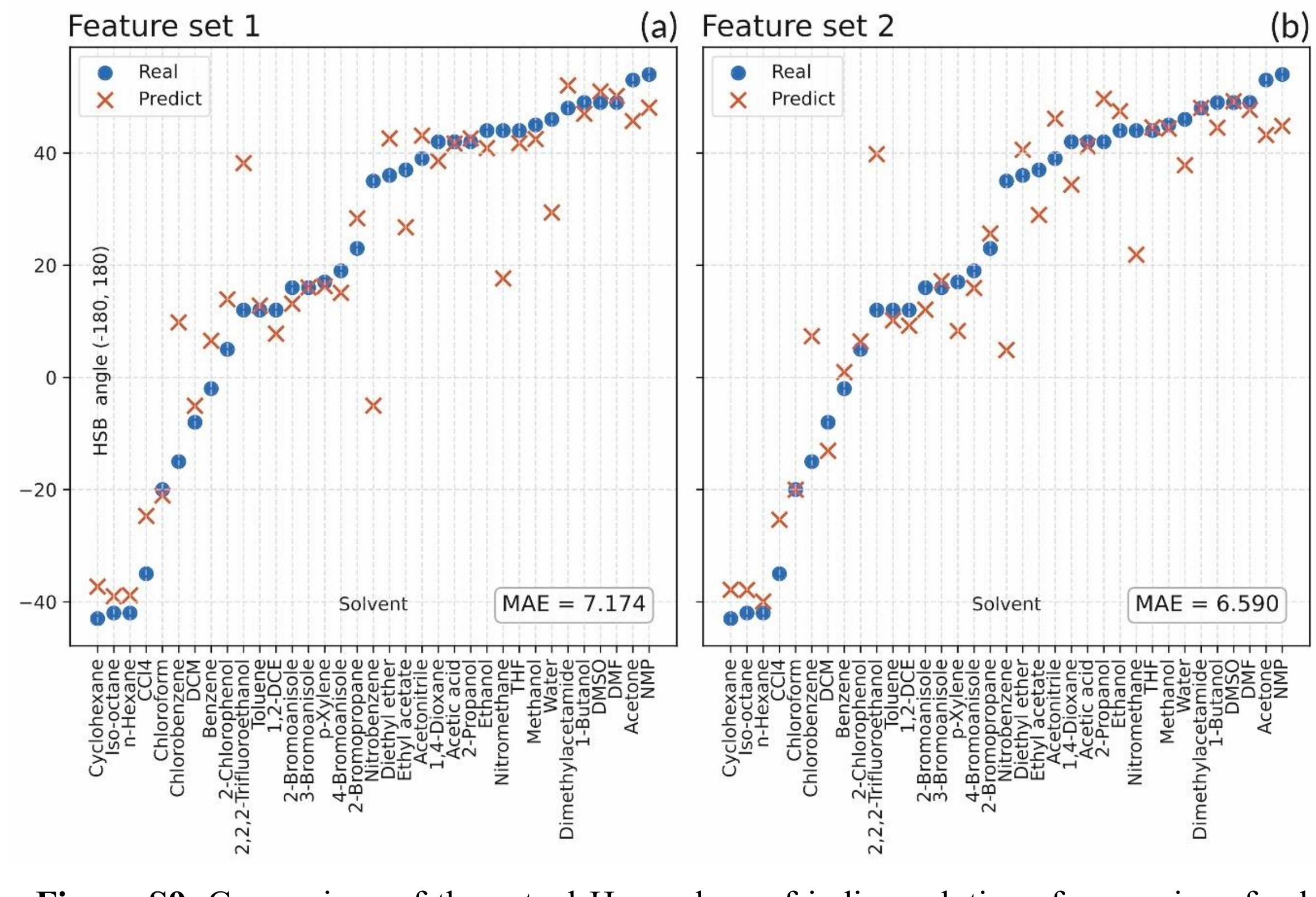

Figure S9. Comparison of the actual Hue values of iodine solutions for a series of solvents with the Hue values predicted by the "Random Forest" algorithm using Feature set 1 (a) and Feature set 2 (b). For each algorithm, the minimum mean absolute error (MAE) in predicting the Hue angle is provided (Table S2).

Table S2. Results of model training on Feature sets 1 and 2 (MAE - Mean absolute error).

	**FEATURE SET 1**		**FEATURE SET 2**	
	Optimal Dataset	MAE	Optimal Dataset	MAE
Linear regression	φ_1	13.362	E_{LUMO}<br>r_2(I-I)<br>ε_r<br>μ<br>n	10.826
Random forest	$\Delta E_{GAP\text{-}1}$<br>$\Delta E_{I2\text{-}Solv1}$<br>$r_1$(I-I)<br>$\varphi_1$	7.174	$\Delta E_{GAP\text{-}1}$<br>$\Delta E_{I2\text{-}Solv1}$<br>$\varepsilon_r$<br>$n^{25}$	6.590
Gradient boosting	$\Delta E_{GAP\text{-}1}$<br>$\Delta E_{I2\text{-}Solv1}$<br>$r_1$(I-I)<br>$\varphi_1$	**6.836**	$\Delta E_{GAP\text{-}1}$<br>$\Delta E_{I2\text{-}Solv1}$<br>$\varepsilon_r$	**5.868**

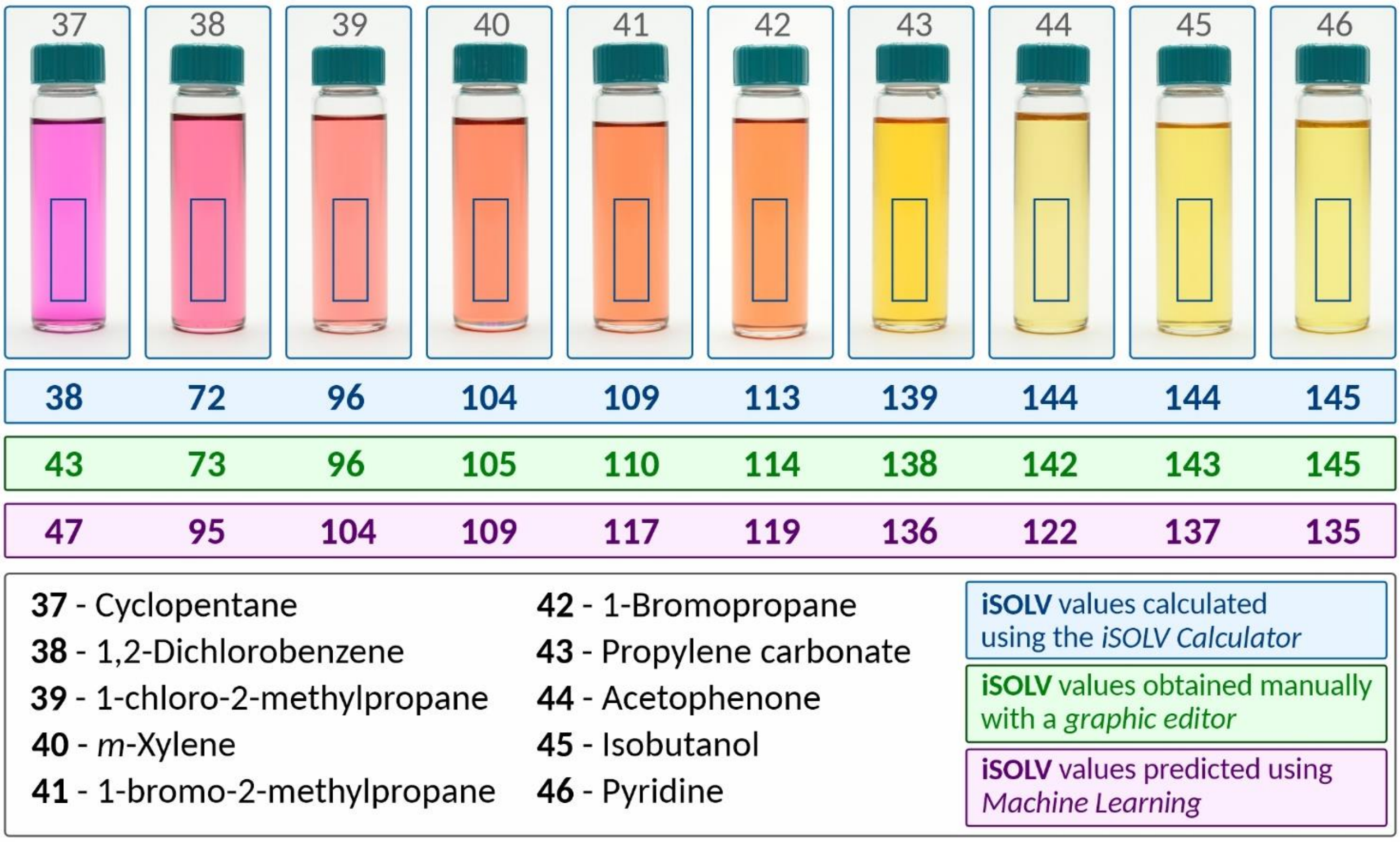

Figure S10. Iodine solutions in 10 different organic solvents for predicting iSolv values using machine learning methods. For each solution, the rectangular color averaging regions are shown, along with the iSolv values obtained automatically using the iSolv Calculator program (highlighted in blue), manually using a graphic editor (highlighted in green) and iSolv values predicted by gradient boosting method with feature set 2 (highlighted in violet).

Table S3. Values of E_{HOMO} (eV) for isolated solvent molecules; difference in energies |LUMO-HOMO| (ΔE_{GAP-1}, eV) for a complex of one solvent molecule with one I_2 molecule; bonding energy of the I_2 molecule with the first solvent molecule ($\Delta E_{I2-Solv1}$, kcal/mol); bonding energy of the I_2 molecule with the second solvent molecule ($\Delta E_{I2-Solv2}$, kcal/mol); interatomic distance I-I in the complex of the I_2 molecule with one solvent molecule (r_1(I-I), Å); interatomic distance I-I in the complex of the I_2 molecule with two solvent molecules (r_2(I-I), Å); maximum positive values of ESP in the σ-hole region of the I_2 molecule within the complex with one solvent molecule (φ_1, a.u.). Calculations were performed using the PBE0 D4 def2TZVP ZORA method.

№	Solvent	E_{HOMO}	ΔE_{GAP-1}	$\Delta E_{I2-Solv1}$	$\Delta E_{I2-Solv2}$	r_1(I-I)	r_2(I-I)	φ_1
37	Cyclopentane	-8.69	3.77	-2.42	-2.47	2.668	2.670	0.1238
38	1,2-Dichlorobenzene	-7.19	3.87	-4.22	-3.74	2.676	2.679	0.1085
39	1-Chloro-2-methylpropane	-8.25	3.95	-5.45	-4.49	2.687	2.695	0.1029
40	*m*-Xylene	-6.79	3.84	-7.36	-5.85	2.697	2.705	0.0931
41	1-Bromo-2-methylpropane	-7.74	3.94	-5.82	-4.25	2.696	2.706	0.0969
42	1-Bromopropane	-7.77	3.94	-6.33	-4.86	2.696	2.708	0.0988
43	Propylene carbonate	-8.54	4.05	-6.42	-3.55	2.687	2.689	0.0889
44	Acetophenone	-7.31	3.99	-6.44	-4.35	2.686	2.689	0.0930
45	Isobutanol	-7.78	4.09	-7.58	-5.28	2.693	2.701	0.0936
46	Pyridine	-7.41	4.32	-12.40	-4.85	2.738	2.733	0.0597

Table S4. The values of the relative permittivity (ε_r), dipole moment (μ, D) and refractive index (n) for solvents used for ML-prediction of the iSolv values.[1,2,6,14]

№	Solvent	ε_r	μ	n
37	Cyclopentane	1.97	0.00	1.4065
38	1,2-Dichlorobenzene	9.93	2.51	1.5491
39	1-Chloro-2-methylpropane	9.96	2.13	1.3970
40	*m*-Xylene	2.37	0.33	1.4972
41	1-Bromo-2-methylpropane	7.20	1.92	1.4362
42	1-Bromopropane	8.09	3.16	1.4343
43	Propylene carbonate	64.14	4.90	1.4189
44	Acetophenone	17.44	3.02	1.5322
45	Isobutanol	17.93	1.78	1.3958
46	Pyridine	12.30	2.30	1.5067

Table S5. The iSolv values predicted by machine learning methods for 10 solutions of iodine in various organic solvents. Also shown are the iSolv values determined manually (iSolv exp. Manually) using a graphic editor and automatically using the iSolv Calculator program (iSolv exp. Automatic).

№	Linear regression FEATURE SET1	Linear regression FEATURE SET2	Random forest FEATURE SET1	Random forest FEATURE SET2	Gradient boosting FEATURE SET1	Gradient boosting FEATURE SET2	iSOLV exp. Automatic	iSOLV exp. Manually
37	68	53	50	49	48	47	38	43
38	98	79	101	100	103	95	72	73
39	108	117	118	120	119	104	96	96
40	127	114	107	102	107	109	104	105
41	120	133	120	120	112	117	109	110
42	116	145	118	119	114	119	113	114
43	135	132	134	135	132	136	139	138
44	127	109	124	126	132	122	144	142
45	126	125	131	137	132	137	144	143
46	190	181	139	136	138	135	145	145

Table S6. The mean absolute error values for various machine learning methods used to predict the iSolv values of 10 different iodine solutions.

	iSolv exp. Automatic	iSolv exp. Manually
Linear regression FEATURE SET1	18.8	17.6
Linear regression FEATURE SET2	20.4	19.2
Random forest FEATURE SET1	12.7	11.4
Random forest FEATURE SET2	12.2	11.0
Gradient boosting FEATURE SET1	10.8	9.6
Gradient boosting FEATURE SET2	10.1	**8.8**

4. Practical use of the method for determining iSolv value and solvent solvating ability based on the iodine solution color

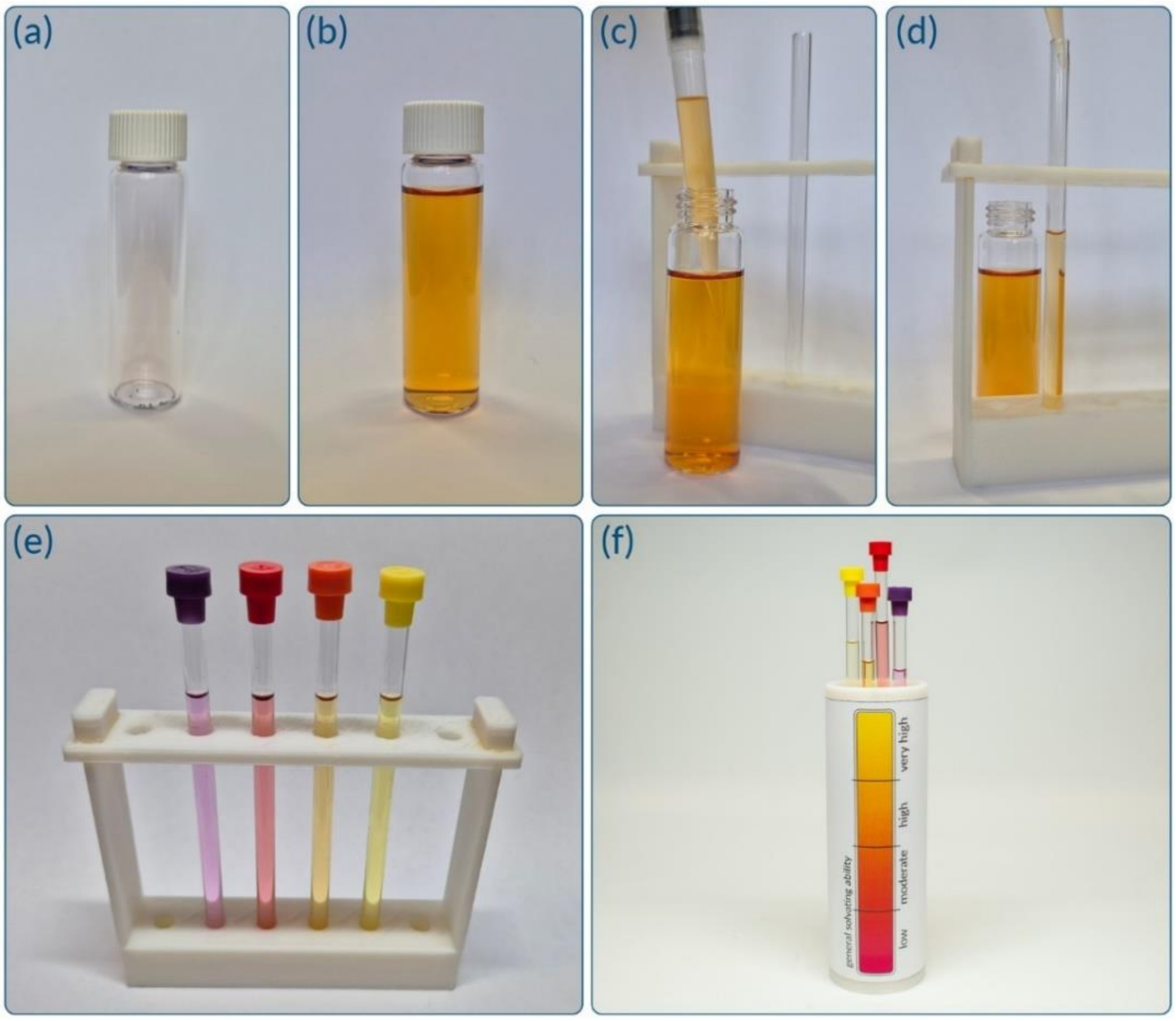

Figure S11. Steps in the preparation of iodine solutions to determine the iSolv value and solvating ability of the solvents: (a) vial with crystal iodine portion; (b) vial filled with iodine solution; (c) withdrawal of a small volume of solution by pipette; (d) filling a 5 mm diameter ampoule with iodine solution; (e) prepared iodine solutions in ampoules; (f) container with color scale and ampoules.

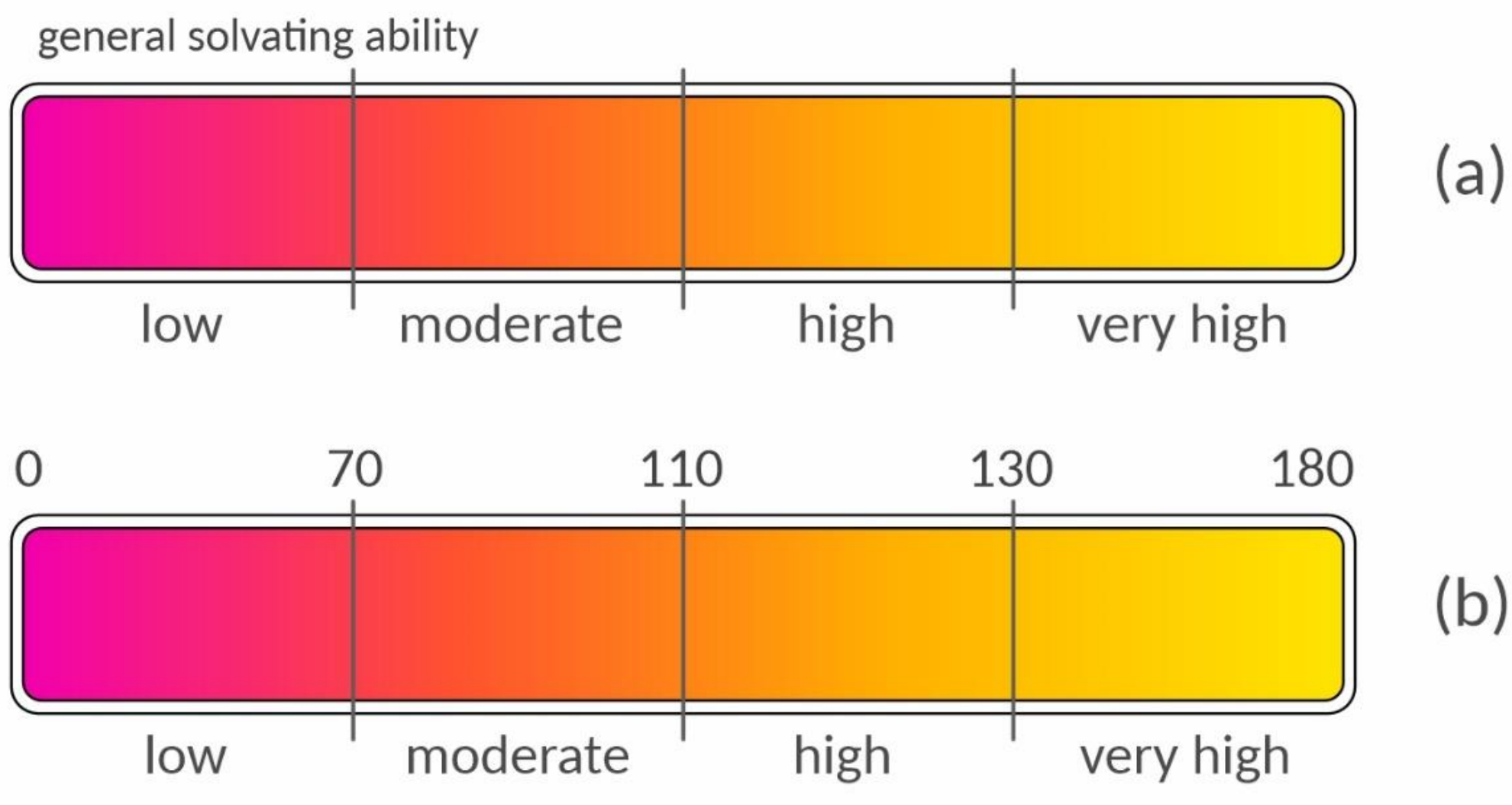

Figure S12. A color scale for the practical determination of general solvating ability on the basis of the color of standard-concentration iodine solutions: (a) iSolv scale without numerical values; (b) iSolv scale with numerical iSolv values. The operational intervals are low, $0 \leq \text{iSolv} < 70$; moderate, $70 \leq \text{iSolv} < 110$; high, $110 \leq \text{iSolv} < 130$; and very high, $130 \leq \text{iSolv} \leq 180$. These are heuristic visual boundaries; iSolv remains a continuous variable.

5. In-house developed software «iSolv Calculator» for Hue and iSolv values calculation: step-by-step user guide

5.1. Requirements for solutions

Recommended concentration: 10^{-3} mol/L for all solutions (analyzed and two reference). Iodine crystals must be fully dissolved. Use transparent, colorless glass vials. Solution color should be uniform, with no visible gradients or precipitates. The tubes used for preparing the analyzed and reference solvents are recommended to be the same. However, they may differ from the tubes shown in this instruction.

5.2. Requirements for photos

Take photos of the solutions under bright, neutral lighting (avoid warm/yellow or colored light). Do not use a colored background; place the solution being photographed on a white, matte background with no extraneous objects. When shooting, position the camera directly facing the vial. Avoid creating shadows and glare on the glass wall of the vessel containing the solution (Figure S13).

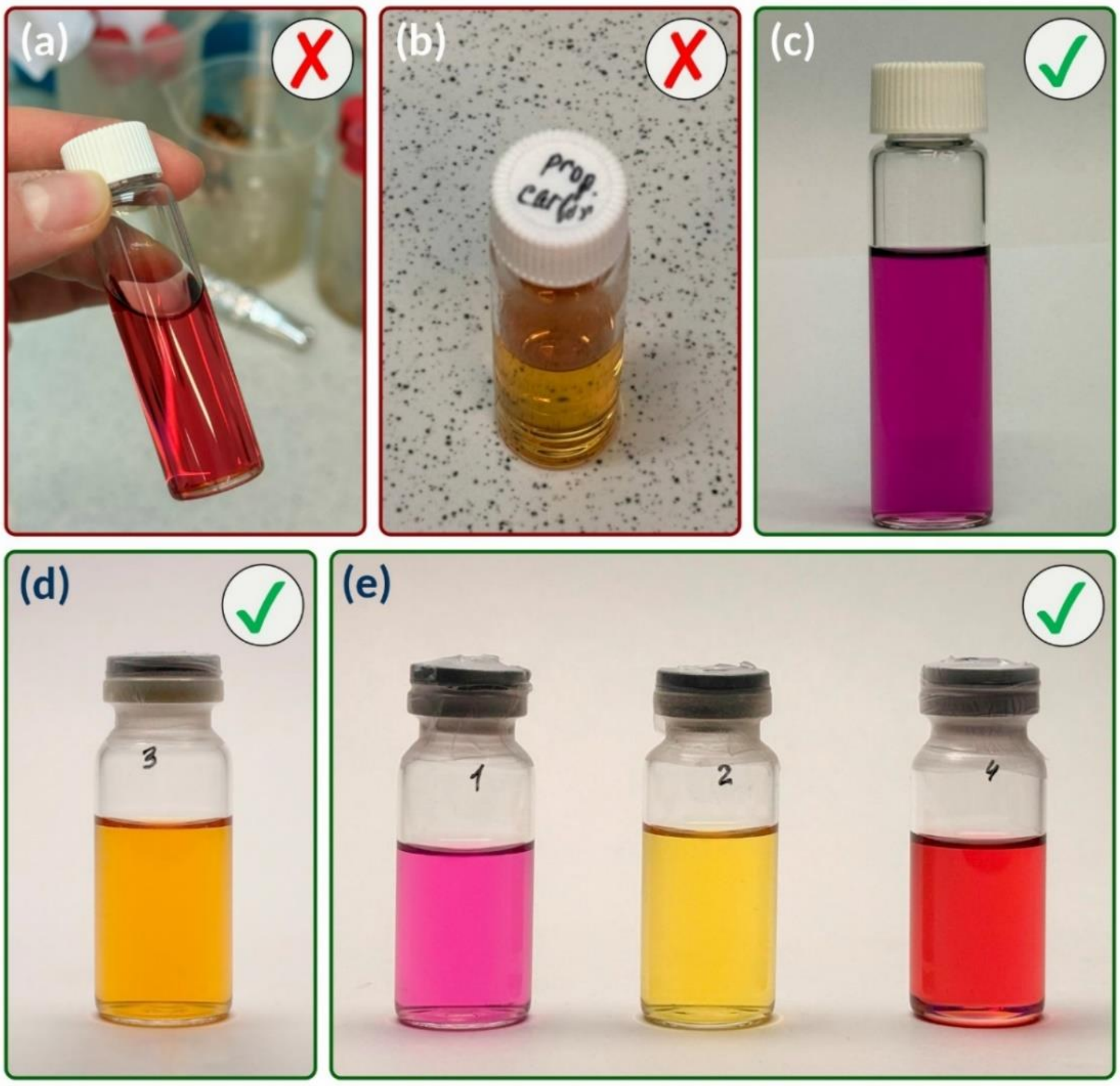

Figure S13. Different photographs of iodine solutions: (a) unsuitable photograph due to excessive glare in the image; (b) unsuitable photograph as the vial is photographed at an unfavorable angle and placed on a spotted background; (c, d) photographs well-suited for analysis; (e) a photograph of three solutions simultaneously (two reference solutions 1 and 2, and the analyzed solution 4), suitable for analysis.

5.3. Guide to working with the web interface of the iSolv Calculator program

The web service for calculating the iSolv descriptor is freely available at: https://isolv.ananikovlab.ai

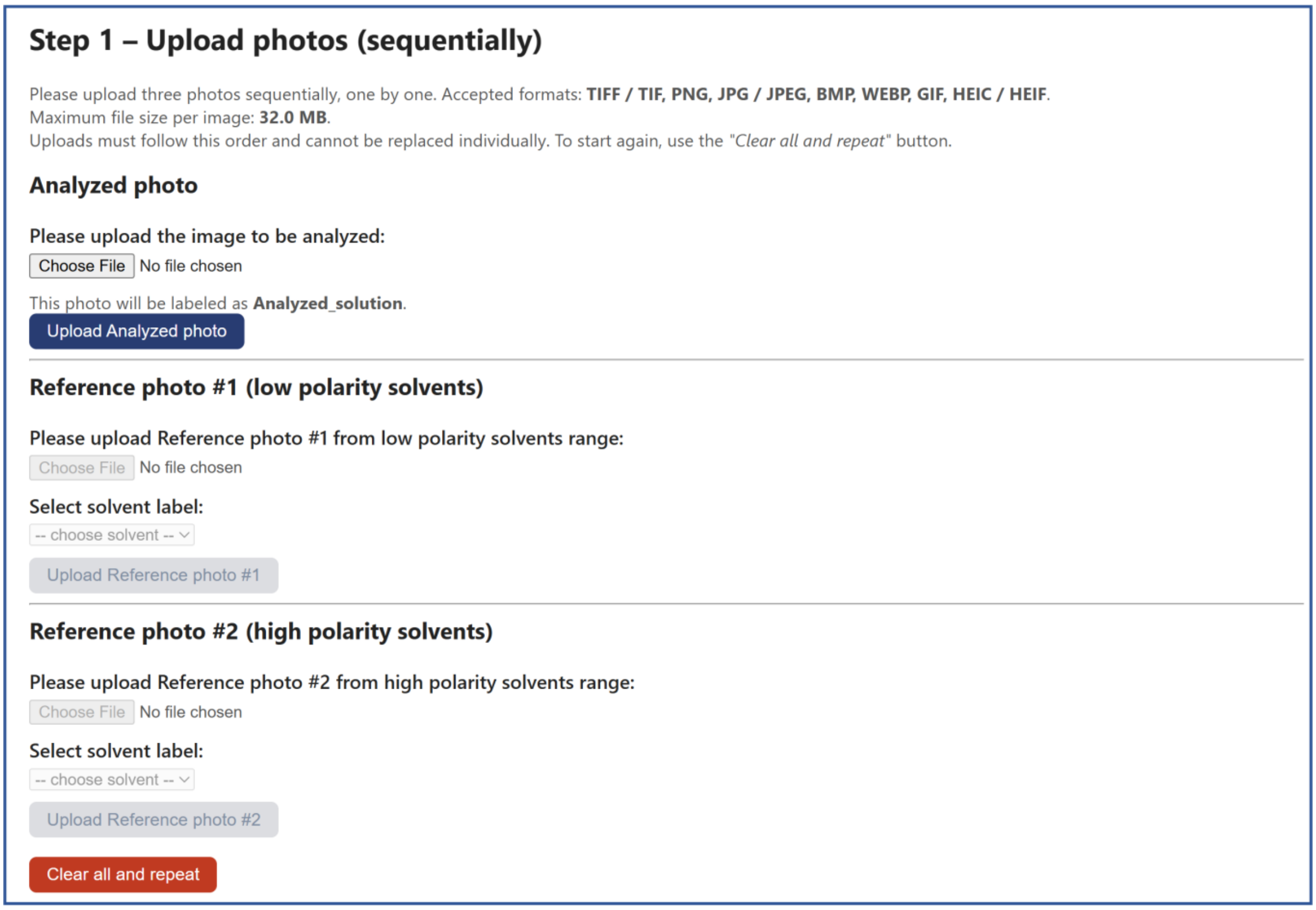

Figure S14. Interface for uploading photos of the analyzed solution and reference solutions.

Step 1 – Upload Photos (Sequentially)

In this step upload three photos in the specific order. The three photos are uploaded one by one, and cannot be individually replaced once all have been uploaded.

Accepted file formats: TIFF/TIF, PNG, JPG/JPEG, BMP, WEBP, GIF, HEIC/HEIF

Maximum file size: 32 MB per image.

1. Upload the analyzed sample photo (Figure S14):

- Click “Choose File” in the “Analyzed photo” section.
- Select the image of the solution you want to analyze from your computer or device.
- Click “Upload Analyzed photo”.
- This image will be labeled “Analyzed_solution”.

After uploading the image that you want to analyze, upload the two reference-solution images (Figure S15).

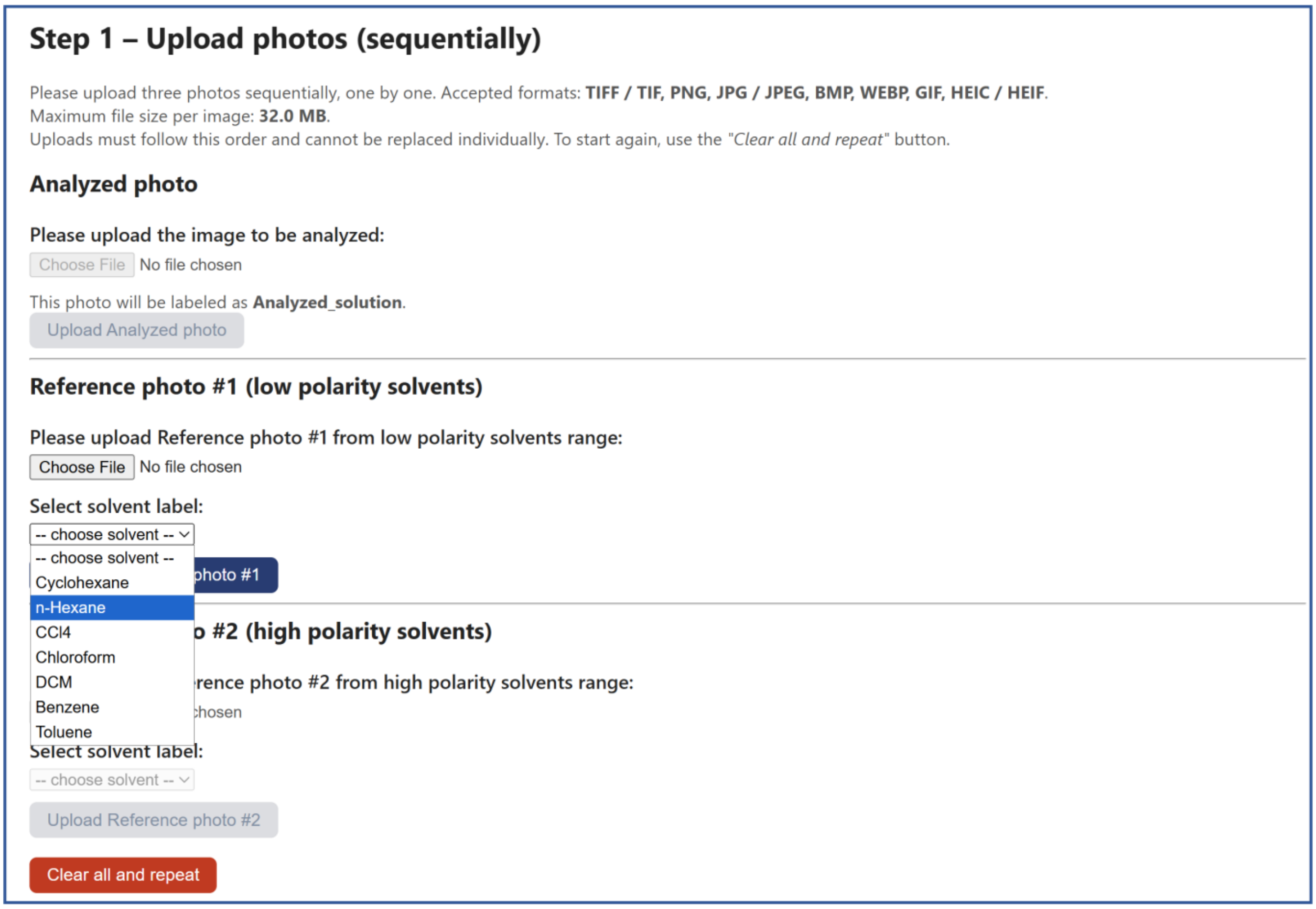

Figure S15. Uploading photos of reference solutions.

Upload reference photo #1 (low-polarity solvents)

● Click “Choose File” in the Reference photo #1 (low polarity solvents) section.

● Select the photo corresponding to a low-polarity reference solvent from your computer or device.

● In “Select solvent label”, choose the appropriate solvent from the dropdown list.

● Click “Upload Reference photo #1”.

Upload reference photo #2 (high-polarity solvents)

● Click “Choose File” in the Reference photo #2 (high polarity solvents) section.

● Select the photo corresponding to a high-polarity reference solvent from your computer or device.

● In “Select solvent label”, choose the appropriate solvent from the dropdown list.

● Click “Upload Reference photo #2”.

Restarting the process

If you need to start over, click “Clear all and repeat”.
This will remove all uploaded images so you can upload them again in the correct order.

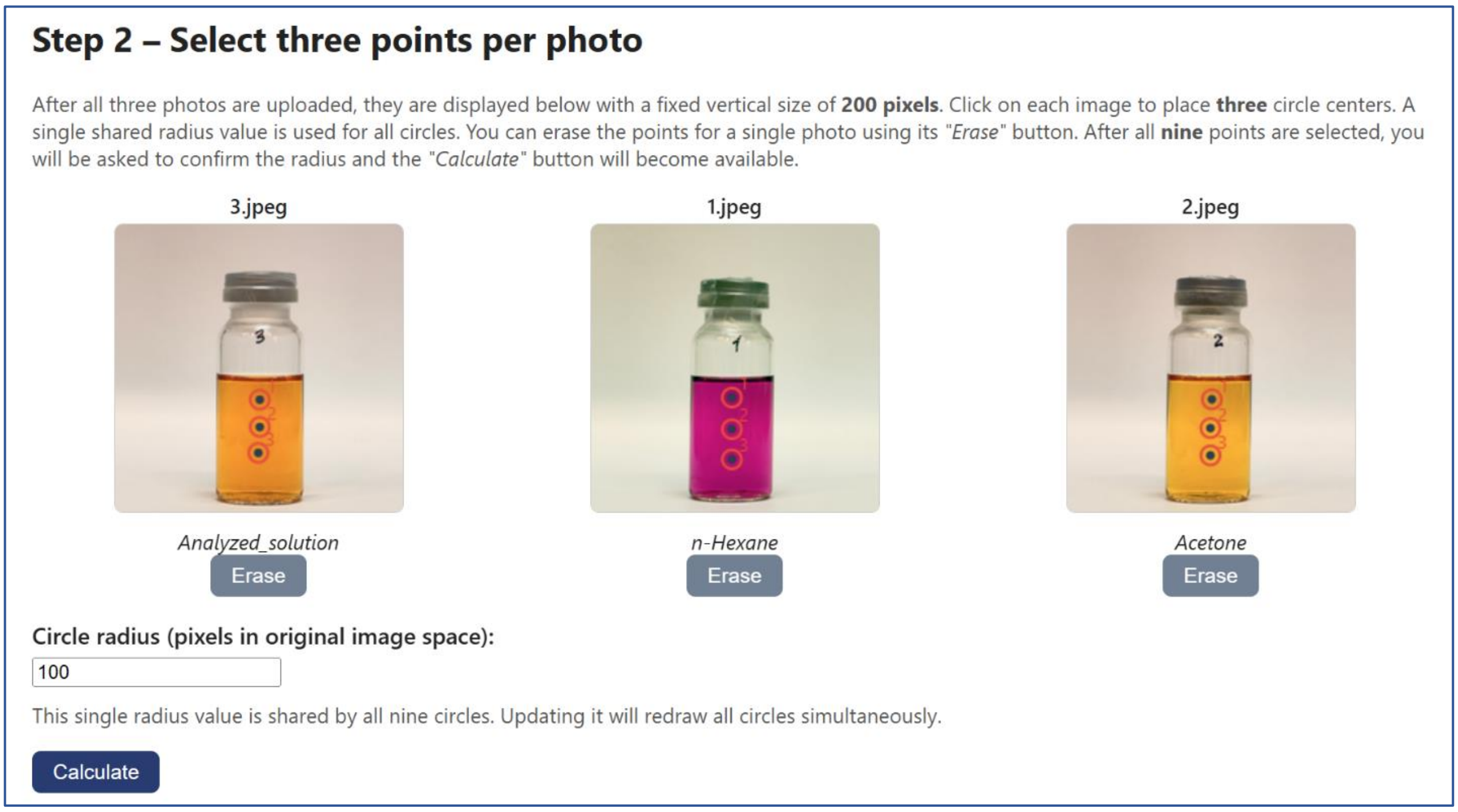

Figure S16. Photographs of iodine solutions with three user-specified points on each photo. For each point, a circle is shown within which the iSolv calculation will be performed.

Step 2 – Select three points per photo

After uploading the three images in Step 1, they will appear on the screen with a fixed vertical size of 200 pixels. In this step you must mark three points on each photo. An area for color averaging, bounded by a circle, will be formed around each user-specified point. The radius of the circle is set in pixels at the scale of the photograph, meaning the visible radius of the circles will vary depending on the photo's resolution. Higher image resolution requires a larger circle radius, and vice versa. Points should be placed only within the area of the colored solution, vertically in the central region of the solution (Figure S16). Additionally, the circles must not intersect and should not be placed too close to the edges of the vial or the liquid meniscus. Figure S17 provides examples of correct and incorrect placement of points on photographs.

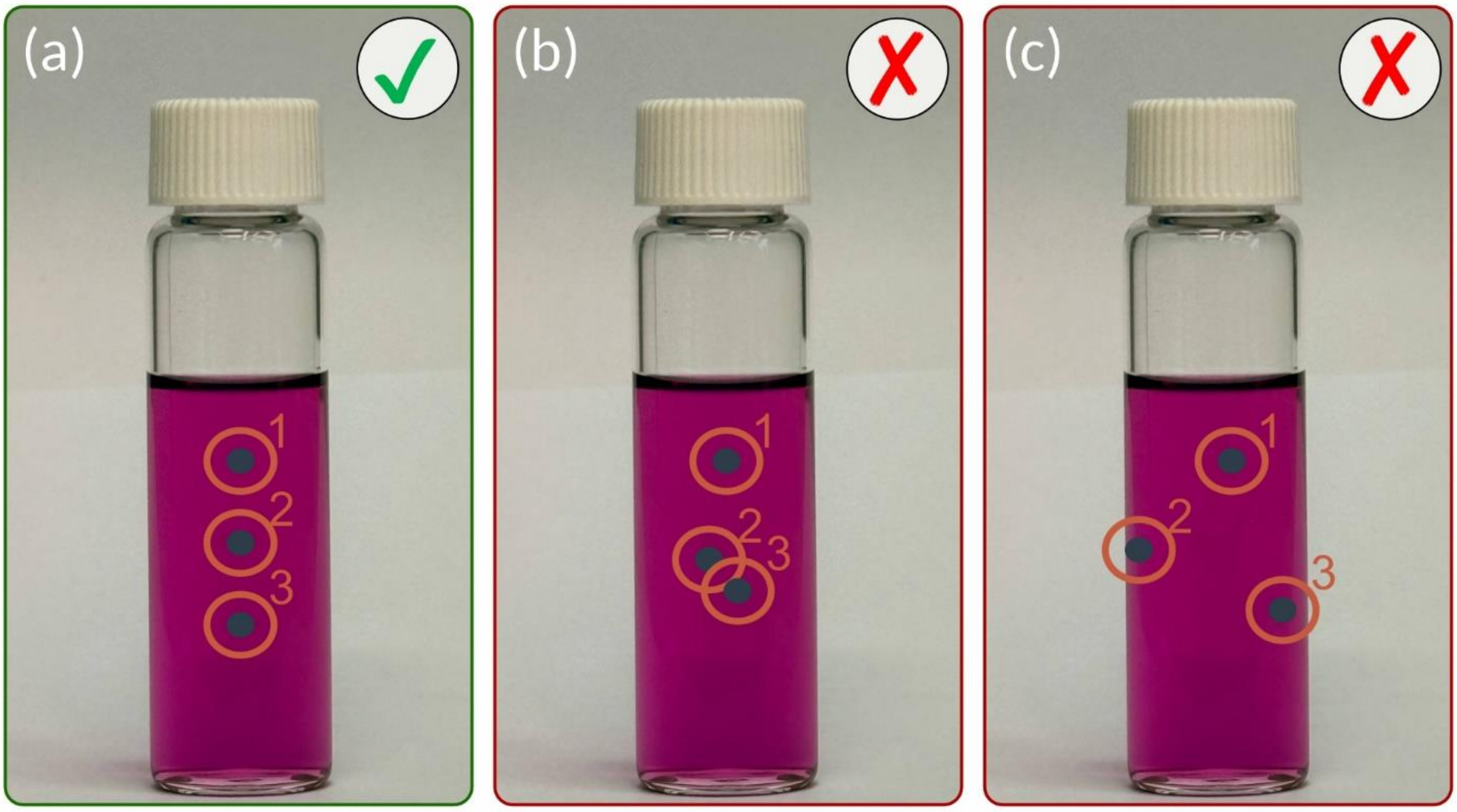

Figure S17. Examples of correct and incorrect placement of points on a photograph of a vial with an iodine solution: (a) correct placement of points; (b) incorrect placement of points because the circles overlap at the given radius; (c) incorrect placement of points because the circles extend beyond the boundaries of the vial.

Examples of file resolution and corresponding circle radii:

3024 × 3024 – recommended circle radius 100 pixels;
400 × 300 – recommended circle radius 10 pixels.

1. Select points on each image

● Click directly on an image to place the center of a circle.
● Select three points on each photo (a total of nine points across all images).
● Each selected point will appear as a circle on the image.

2. The circle radius is defined in the field "Circle radius (pixels in original image space)".

● The default value is 10 pixels.
● This single radius value is applied to all circles on all images.
● Changing the radius will automatically redraw all circles.

3. Remove points if necessary

● If you want to reset the points for one image, click the "Erase" button under that photo.
● This will remove all three circles from that specific image so you can select them again.

4. Run the calculation

● After all nine points (three per photo) are selected press Enter or click the "Calculate" button to start the analysis.

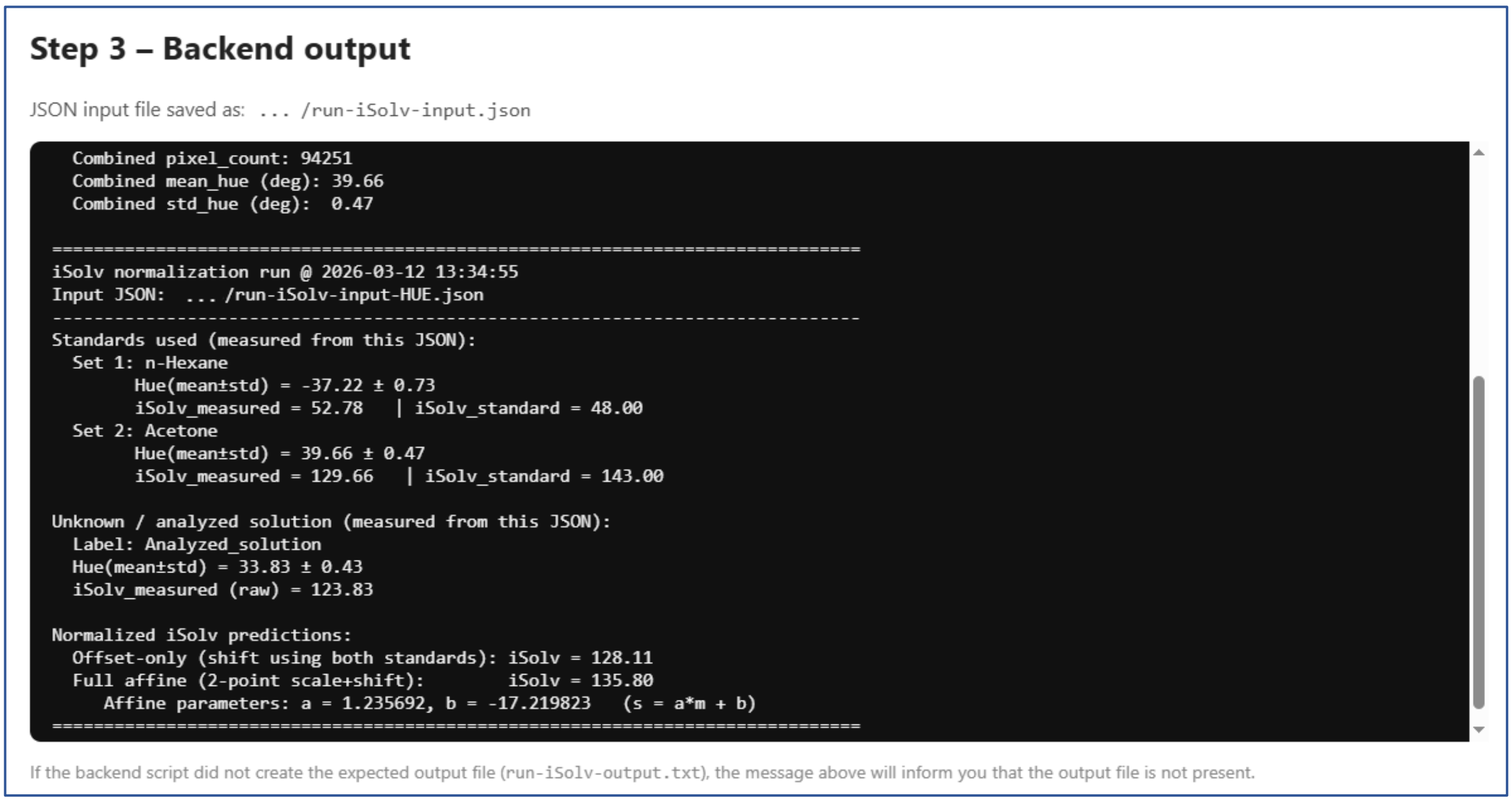

Figure S18. Window with iSolv calculation results for reference solutions and for the analyzed solution.

Step 3 – Backend output

After clicking "Calculate" in Step 2, the system processes the selected regions and displays the backend analysis output (Figure S18).

1. Input data confirmation

At the top of the output, the system indicates the location of the generated JSON input file, which contains the processed image data used for the calculation.

2. Reference solvent calibration

The program first analyzes the reference solvents selected in Step 1.

For each reference solvent (e.g., *n*-Hexane and Acetone), the following parameters are displayed:

- Mean hue value (degrees) calculated from the selected circular regions
- Standard deviation of hue
- Measured iSolv value
- Literature (standard) iSolv value

These reference values are used to normalize the scale for the analyzed solution.

3. Analyzed solution data

The output then displays the calculated values for the analyzed sample, including:

- Label of the analyzed solution
- Mean hue value
- Standard deviation
- Raw (unnormalized) iSolv value

4. Normalized iSolv prediction

Finally, the system reports the predicted iSolv value using calibration with the reference solvents. Two normalization approaches may be displayed:

- Offset-only correction (shift based on the reference solvents)
- Full affine normalization using two-point calibration

The affine transformation parameters (a and b) used for scaling are also shown.

So, as the result of the iSolv calculation, you can use either iSolv (offset-only), iSolv (full affine), or their average value.

5.4. Examples of test calculations for the iSolv descriptor

Five sets of solutions, each containing three solutions, were used for test calculations with iSolv. In each set, two solutions are reference (control) solutions and remain constant across all sets, while one solution is the analyzed solution and varies in each set. Thus, five different analyzed solutions were tested under constant reference conditions (Tables S7-S10). Table S7 contains the results of iSolv calculations for the photographs used in the main text of the article (Figure 2) to verify the reproducibility of iSolv values using the developed software.

Reference solutions (used unchanged across all five sets):
Reference solution No. 1 – low polarity (purple), solvent – *n*-hexane;
Reference solution No. 2 – high polarity (yellow), solvent – acetone.

Analyzed solutions:
Analyzed solution 1 – low polarity;
Analyzed solution 2 – high polarity;
Analyzed solution 3 – medium, closer to low;
Analyzed solution 4 – medium, closer to high;
Analyzed solution 5 – medium, closer to low.

In Tables S7-S10, the following notations for quantities are used: iSolv_meas. – iSolv values calculated by the software for *n*-hexane and acetone; iSolv1 – iSolv value calculated by the software for the analyzed solution using the "Offset-only (shift using both stand.)" correction; iSolv2 – iSolv value calculated by the software for the analyzed solution using the "Full affine (2-point scale+shift)" correction; iSolv^{-} – the arithmetic mean of iSolv1 and iSolv2 (it is recommended to use the iSolv^{-} value as the final result); iSolv_ref. – the reference iSolv value for the analyzed solvent, determined manually in this work (Figure 2); Δ1 – the difference [(iSolv_ref.) – (iSolv1)]; Δ2 – the difference [(iSolv_ref.) – (iSolv2)]; Δ^{-} – the difference [(iSolv_ref.) – (iSolv^{-})].

Table S7. iSolv values for photographs of standard solutions (*n*-hexane and acetone) and analyzed solutions obtained under LED lighting conditions (5600 K) using a Nikon D610 camera (each photograph contains one vial with a solution, circle radius 30 pixels, reference iSolv values are 48 for *n*-hexane and 143 for acetone; the resolution of each photo is 397×1324 pixels). See page S20 for an explanation of the symbols used in the table.

n-hexane iSolv_meas.	*acetone* iSolv_meas.	Analyzed solution	iSolv1	iSolv2	iSolv⁻	iSolv_ref.	**Δ1**	**Δ2**	**Δ⁻**
47.0	142.7	3_methanol	134.2	134.0	134.1	135.0	**0.8**	**1.0**	**0.9**
46.9	142.7	4_benzene	87.7	87.7	87.7	88.0	**0.3**	**0.3**	**0.3**
47.1	142.5	5_diethyl ether	125.5	125.3	125.4	126.0	**0.5**	**0.7**	**0.6**
47.2	142.7	6_DCM	81.8	81.9	81.85	82.0	**0.2**	**0.1**	**0.15**
47.1	142.7	7_cyclohexane	47.3	47.5	47.4	47.0	**-0.3**	**-0.5**	**-0.4**

Table S8. iSolv values for photographs of standard solutions (*n*-hexane and acetone) and analyzed solutions obtained under laboratory lighting conditions using a mobile phone camera (each photograph contains one vial with a solution, circle radius 30 pixels, reference iSolv values are 48 for *n*-hexane and 143 for acetone; the resolution of each photo is 3024×3024 pixels). See page S20 for an explanation of the symbols used in the table.

n-hexane iSolv_meas.	*acetone* iSolv_meas.	Analyzed solution	iSolv1	iSolv2	iSolv⁻	iSolv_ref.	**Δ1**	**Δ2**	**Δ⁻**
53.3	129.7	3_methanol	128.1	136.0	132.0	135.0	**6.9**	**-1.0**	**3.0**
53.3	129.8	4_benzene	86.8	84.7	85.8	88.0	**1.2**	**3.3**	**2.2**
53.3	129.8	5_diethyl ether	118.2	123.7	120.9	126.0	**7.8**	**2.3**	**5.1**
53.3	129.8	6_DCM	76.8	72.4	74.6	82.0	**5.2**	**9.6**	**7.4**
53.3	129.8	7_cyclohexane	55.7	46.1	50.9	47.0	**-8.7**	**0.9**	**-3.9**

Table S9. iSolv values for photographs of standard solutions (*n*-hexane and acetone) and analyzed solutions obtained under laboratory lighting conditions using a mobile phone camera (each photograph contains one vial with a solution, circle radius 100 pixels, reference iSolv values are 48 for *n*-hexane and 143 for acetone; the resolution of each photo is 3024×3024 pixels). See page S20 for an explanation of the symbols used in the table.

n-hexane iSolv_meas.	*acetone* iSolv_meas.	Analyzed solution	iSolv1	iSolv2	iSolv⁻	iSolv_ref.	**Δ1**	**Δ2**	**Δ⁻**
52.9	129.6	3_methanol	128.1	135.8	132.0	135.0	**6.9**	**-0.8**	**3.1**
53.2	129.7	4_benzene	87.0	85.0	86.0	88.0	**1.0**	**3.0**	**2.0**
53.0	129.7	5_diethyl ether	119.0	124.6	121.8	126.0	**7.0**	**1.4**	**4.2**
53.0	129.8	6_DCM	76.3	71.8	74.1	82.0	**5.7**	**10.2**	**7.9**
53.1	129.7	7_cyclohexane	55.3	45.7	50.9	47.0	**-8.3**	**1.3**	**-3.9**

Table S10. iSolv values for photographs of standard solutions (*n*-hexane and acetone) and analyzed solutions obtained under laboratory lighting conditions using a mobile phone camera (each photograph contains three vials with a solution, circle radius 30 pixels, reference iSolv values are 48 for *n*-hexane and 143 for acetone; the resolution of each photo is 4032×3024 pixels). See page S20 for an explanation of the symbols used in the table.

n-hexane iSolv_meas.	*acetone* iSolv_meas.	Analyzed solution	iSolv1	iSolv2	iSolv⁻	iSolv_ref.	**Δ1**	**Δ2**	**Δ⁻**
51.8	133.8	3_methanol	129.6	135.0	132.3	135.0	**5.4**	**0.0**	**2.7**
51.3	132.5	4_benzene	87.0	85.6	86.3	88.0	**1.0**	**2.4**	**1.7**
52.3	132.7	5_diethyl ether	120.1	124.6	122.4	126.0	**5.9**	**1.4**	**3.7**
51.4	133.7	6_DCM	84.5	82.8	83.7	82.0	**-2.5**	**-0.8**	**-1.7**
51.3	133.0	7_cyclohexane	55.0	48.4	51.7	47.0	**-8.0**	**-1.4**	**-4.7**

6. Reproducibility of the color measurements

Reproducibility was examined at three levels. Preparation-to-preparation consistency was assessed by including independent duplicate solutions of n-hexane, chloroform, benzene, 1,2-dichloroethane, p-xylene, diethyl ether, nitromethane, and 1,2-dichlorobenzene in the 50-solution visual-categorization set (Figures S19 and S20). Concentration robustness was evaluated for DCM and MeOH by varying iodine loading from 1.5 to 25 mg per 10 mL around the standard 2.5 mg loading (Figure S21). Camera dependence was evaluated for five solutions using 10 mobile-phone cameras; the resulting across-camera standard deviations were 1.8–4.5 iSolv units (Tables S11–S21).

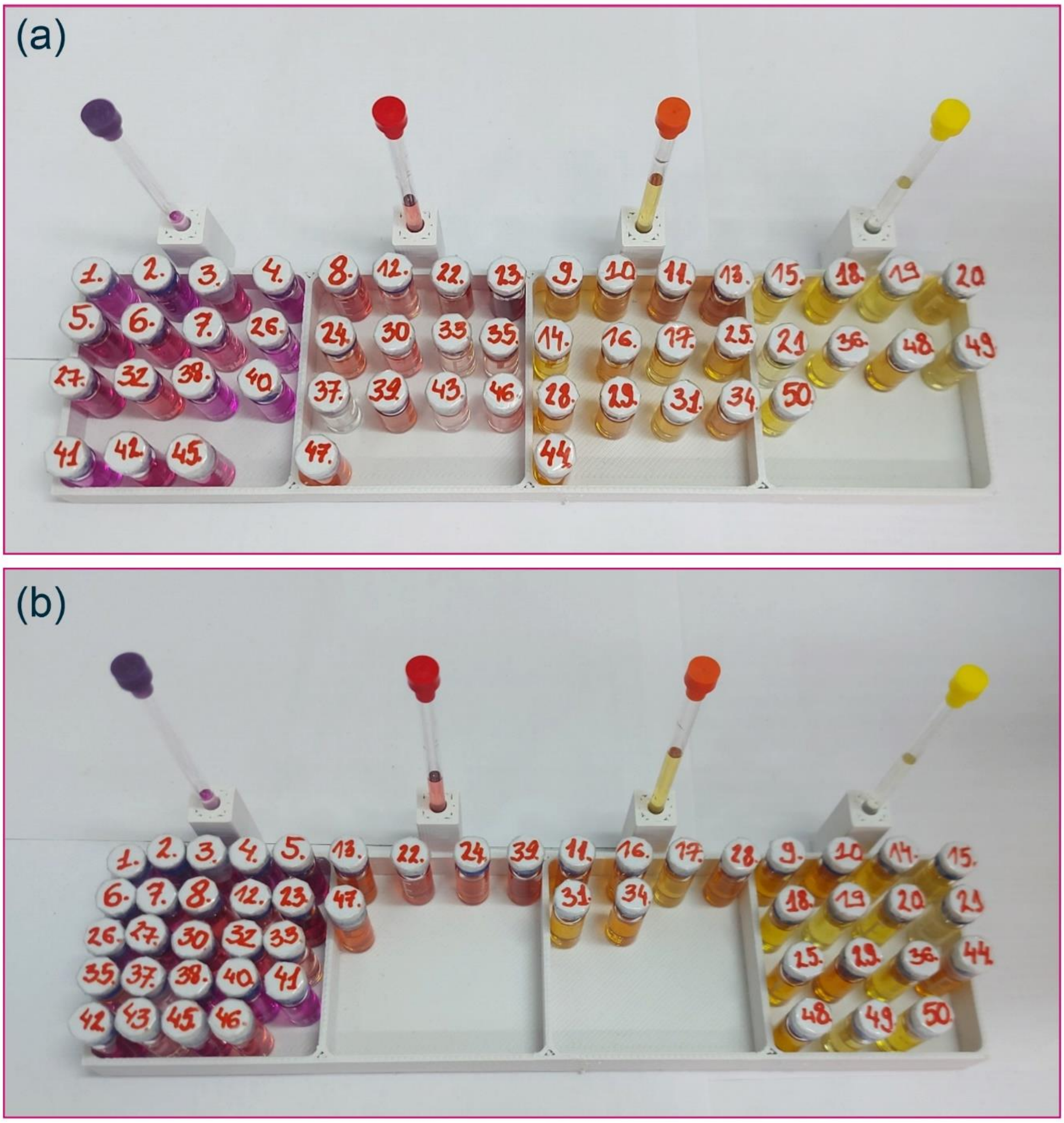

Figure S19. Examples of distribution of iodine solutions into four color groups by two researchers.

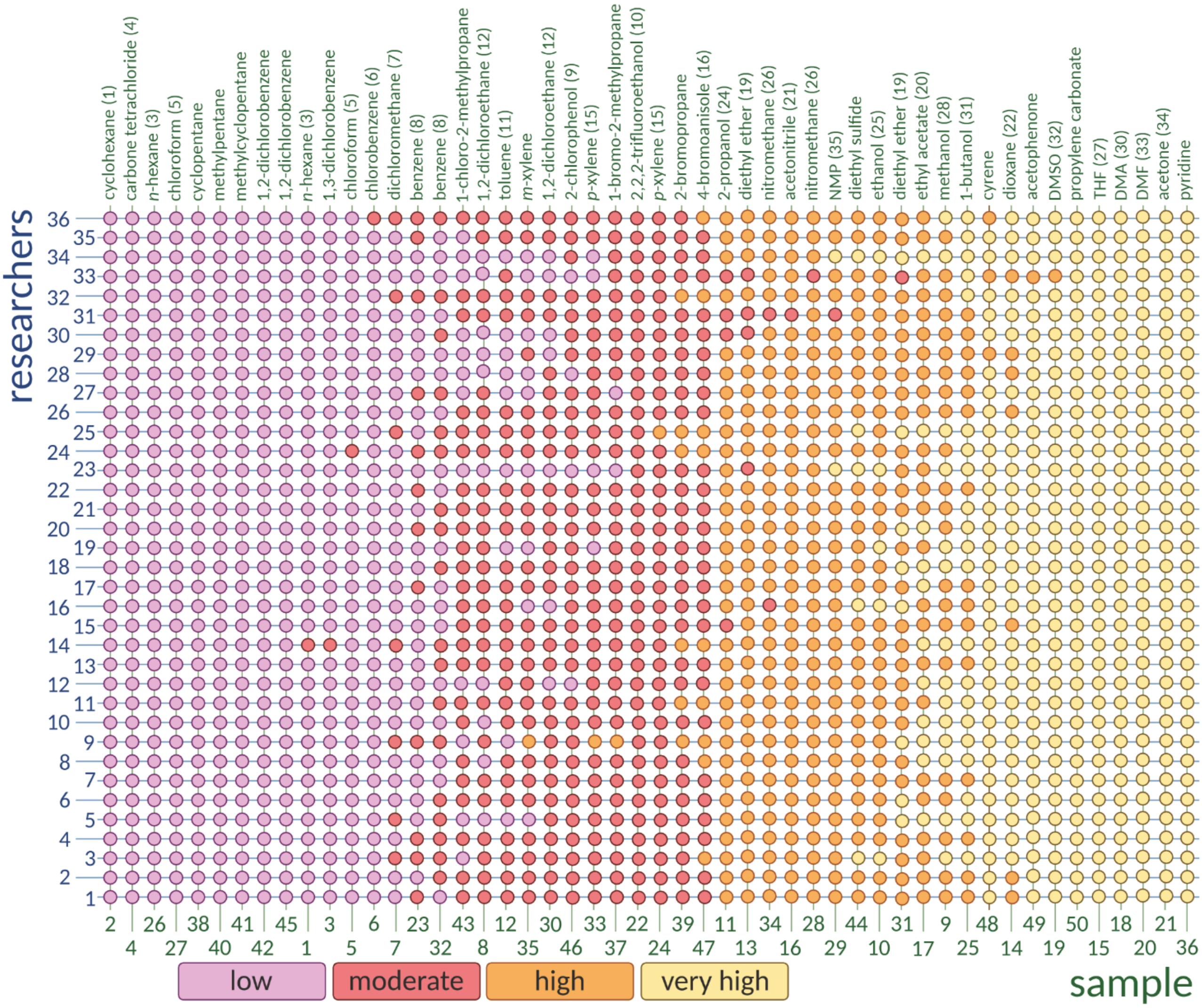

Figure S20. Distribution of solvents by degree of solvating ability, on the basis of the color of the iodine solution, as assessed by 36 researchers. For some solvents (e.g., benzene), two series of experiments were conducted to check reproducibility. The number of the solvents from the main set, as indicated in Figure 2, is given in parentheses next to the solvent name.

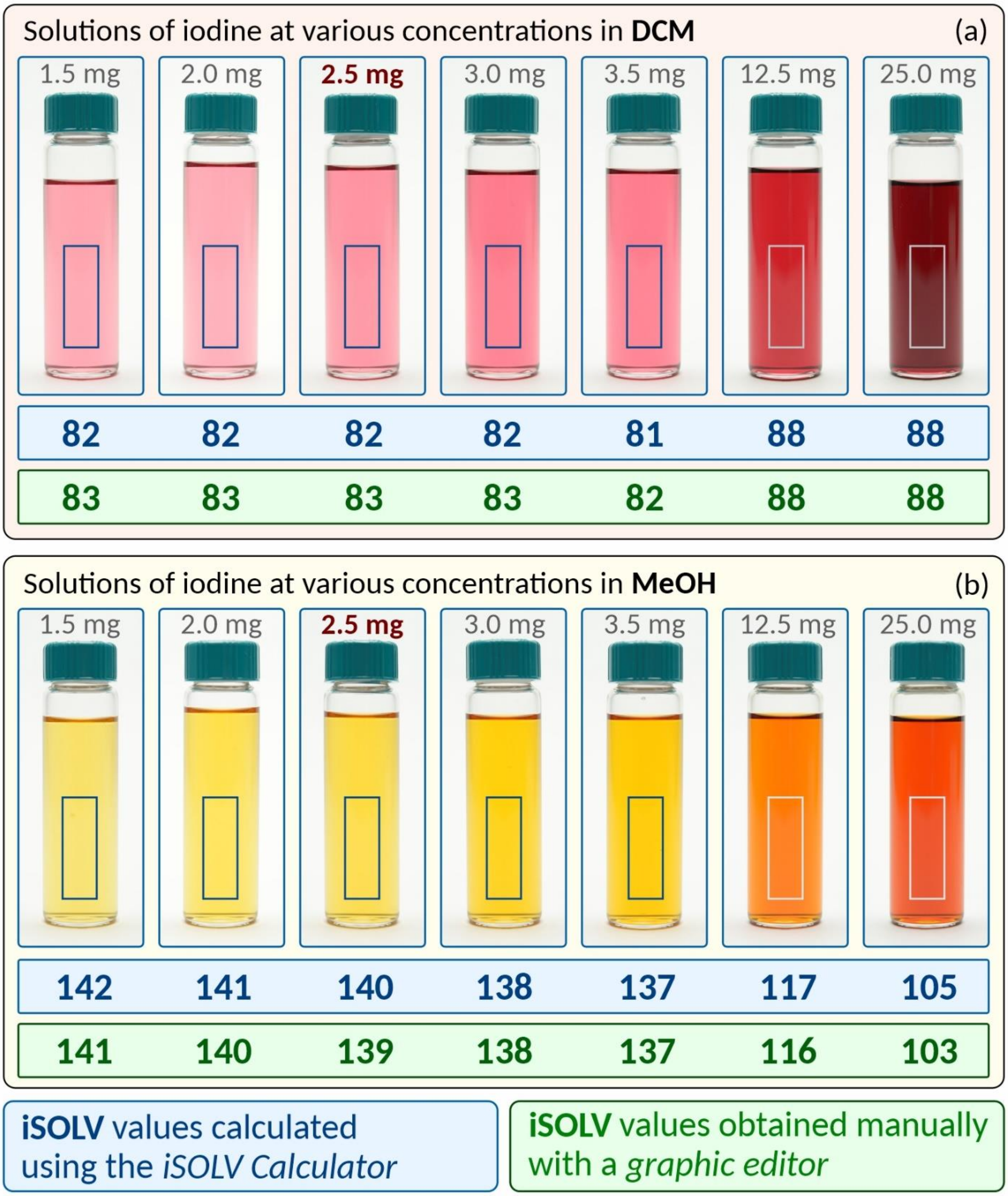

Figure S21. Solutions of iodine in DCM (a) and MeOH (b) containing different amounts of iodine (indicated in mg). The amount of iodine in the solution used throughout this work is highlighted in red (2.5 mg). For each solution, the rectangular color averaging regions are shown, along with the iSolv values obtained automatically using the iSolv Calculator program (highlighted in blue) and manually using a graphic editor (highlighted in green).

Table S11. iSolv values for photographs of standard solutions (*n*-hexane and acetone) and analyzed solutions obtained under laboratory lighting conditions using a mobile phone camera 1 (each photograph contains one vial with a solution, circle radius 30 pixels, reference iSolv values are 48 for *n*-hexane and 143 for acetone; the resolution of each photo is 960×1280 pixels).

n-hexane iSolv_meas.	*acetone* iSolv_meas.	Analyzed solution	iSolv1	iSolv2	iSolv⁻	iSolv_ref.	**Δ1**	**Δ2**	**Δ⁻**
52.86	142.23	3_methanol	132.58	134.91	133.75	135.0	**2.42**	**0.09**	**1.255**
52.96	142.24	4_benzene	89.65	89.28	89.47	88.0	**-1.65**	**-1.28**	**-1.465**
52.59	142.19	5_diethyl ether	121.55	123.12	122.34	126.0	**4.45**	**2.88**	**3.665**
52.88	142.24	6_DCM	74.13	72.78	73.46	82.0	**7.87**	**9.22**	**8.545**
52.56	142.2	7_cyclohexane	49.92	47.19	48.56	47.0	**-2.92**	**-0.19**	**-1.555**

Table S12. iSolv values for photographs of standard solutions (*n*-hexane and acetone) and analyzed solutions obtained under laboratory lighting conditions using a mobile phone camera 2 (each photograph contains one vial with a solution, circle radius 30 pixels, reference iSolv values are 48 for *n*-hexane and 143 for acetone; the resolution of each photo is 1440×1920 pixels).

n-hexane iSolv_meas.	*acetone* iSolv_meas.	Analyzed solution	iSolv1	iSolv2	iSolv⁻	iSolv_ref.	**Δ1**	**Δ2**	**Δ⁻**
44.85	139.78	3_methanol	137.24	137.27	137.26	135	**-2.24**	**-2.27**	**-2.255**
44.91	139.83	4_benzene	94.26	94.26	94.26	88	**-6.26**	**-6.26**	**-6.26**
44.99	139.71	5_diethyl ether	126.13	126.22	126.18	126	**-0.13**	**-0.22**	**-0.175**
44.91	139.9	6_DCM	78.47	78.46	78.47	82	**3.53**	**3.54**	**3.535**
45.05	139.73	7_cyclohexane	47.51	47.35	47.43	47	**-0.51**	**-0.35**	**-0.43**

Table S13. iSolv values for photographs of standard solutions (*n*-hexane and acetone) and analyzed solutions obtained under laboratory lighting conditions using a mobile phone camera 3 (each photograph contains one vial with a solution, circle radius 30 pixels, reference iSolv values are 48 for *n*-hexane and 143 for acetone; the resolution of each photo is 3000×3000 pixels).

n-hexane iSolv_meas.	*acetone* iSolv_meas.	Analyzed solution	iSolv1	iSolv2	iSolv⁻	iSolv_ref.	**Δ1**	**Δ2**	**Δ⁻**
41.26	138.02	3_methanol	138.35	137.57	137.96	135	**-3.35**	**-2.57**	**-2.96**
41.55	137.79	4_benzene	94.74	94.75	94.75	88	**-6.74**	**-6.75**	**-6.745**
41.22	137.82	5_diethyl ether	129.63	129.06	129.35	126	**-3.63**	**-3.06**	**-3.345**
41.00	137.67	6_DCM	80.93	81.19	81.06	82	**1.07**	**0.81**	**0.94**
40.69	137.90	7_cyclohexane	46.91	48.01	47.46	47	**0.09**	**-1.01**	**-0.46**

Table S14. iSolv values for photographs of standard solutions (*n*-hexane and acetone) and analyzed solutions obtained under laboratory lighting conditions using a mobile phone camera 4 (each photograph contains one vial with a solution, circle radius 30 pixels, reference iSolv values are 48 for *n*-hexane and 143 for acetone; the resolution of each photo is 3000×3000 pixels).

n-hexane iSolv_meas.	*acetone* iSolv_meas.	Analyzed solution	iSolv1	iSolv2	iSolv^{-}	iSolv_ref.	**Δ1**	**Δ2**	**Δ^{-}**
41.45	132.71	3_methanol	138.02	136.93	137.48	135	**-3.02**	**-1.93**	**-2.475**
41.57	138.89	4_benzene	96.57	96.54	96.56	88	**-8.57**	**-8.54**	**-8.555**
41.6	138.94	5_diethyl ether	129.82	128.99	129.41	126	**-3.82**	**-2.99**	**-3.405**
42.02	138.78	6_DCM	80.77	81.04	80.91	82	**1.23**	**0.96**	**1.095**
41.25	138.94	7_cyclohexane	45.56	46.94	46.25	47	**1.44**	**0.06**	**0.75**

Table S15. iSolv values for photographs of standard solutions (*n*-hexane and acetone) and analyzed solutions obtained under laboratory lighting conditions using a mobile phone camera 5 (each photograph contains one vial with a solution, circle radius 30 pixels, reference iSolv values are 48 for *n*-hexane and 143 for acetone; the resolution of each photo is 960×1280 pixels).

n-hexane iSolv_meas.	*acetone* iSolv_meas.	Analyzed solution	iSolv1	iSolv2	iSolv^{-}	iSolv_ref.	**Δ1**	**Δ2**	**Δ^{-}**
56.82	137.91	3_methanol	130.56	136.58	133.57	135	**4.44**	**-1.58**	**1.43**
56.81	137.82	4_benzene	91.22	90.48	90.85	88	**-3.22**	**-2.48**	**-2.85**
56.70	137.87	5_diethyl ether	121.28	125.67	123.48	126	**4.72**	**0.33**	**2.525**
56.81	137.75	6_DCM	78.32	75.34	76.83	82	**3.68**	**6.66**	**5.17**
56.78	137.87	7_cyclohexane	52.22	44.8	48.51	47	**-5.22**	**2.2**	**-1.51**

Table S16. iSolv values for photographs of standard solutions (*n*-hexane and acetone) and analyzed solutions obtained under laboratory lighting conditions using a mobile phone camera 6 (each photograph contains one vial with a solution, circle radius 30 pixels, reference iSolv values are 48 for *n*-hexane and 143 for acetone; the resolution of each photo is 3072×3072 pixels).

n-hexane iSolv_meas.	*acetone* iSolv_meas.	Analyzed solution	iSolv1	iSolv2	iSolv^{-}	iSolv_ref.	**Δ1**	**Δ2**	**Δ^{-}**
46.27	141.65	3_methanol	137.07	136.90	136.99	135	**-2.07**	**-1.9**	**-1.985**
46.56	142.23	4_benzene	92.34	92.37	92.36	88	**-4.34**	**-4.37**	**-4.355**
45.99	141.94	5_diethyl ether	127.76	127.45	127.61	126	**-1.76**	**-1.45**	**-1.605**
46.75	141.77	6_DCM	74.76	74.76	74.76	82	**7.24**	**7.24**	**7.24**
46.37	141.82	7_cyclohexane	46.20	46.43	46.32	47	**0.8**	**0.57**	**0.685**

Table S17. iSolv values for photographs of standard solutions (*n*-hexane and acetone) and analyzed solutions obtained under laboratory lighting conditions using a mobile phone camera 7 (each photograph contains one vial with a solution, circle radius 30 pixels, reference iSolv values are 48 for *n*-hexane and 143 for acetone; the resolution of each photo is 960×1280 pixels).

n-hexane iSolv_meas.	*acetone* iSolv_meas.	Analyzed solution	iSolv1	iSolv2	iSolv⁻	iSolv_ref.	**Δ1**	**Δ2**	**Δ⁻**
42.88	141.43	3_methanol	138.66	137.10	137.88	135	**-3.66**	**-2.1**	**-2.88**
41.73	141.43	4_benzene	96.88	96.62	96.75	88	**-8.88**	**-8.62**	**-8.75**
42.54	141.51	5_diethyl ether	125.70	121.57	123.64	126	**0.3**	**4.43**	**2.365**
42.72	141.62	6_DCM	80.55	81.14	80.85	82	**1.45**	**0.86**	**1.155**
42.55	141.55	7_cyclohexane	42.78	44.91	43.85	47	**4.22**	**2.09**	**3.155**

Table S18. iSolv values for photographs of standard solutions (*n*-hexane and acetone) and analyzed solutions obtained under laboratory lighting conditions using a mobile phone camera 8 (each photograph contains three vials with a solution, circle radius 30 pixels, reference iSolv values are 48 for *n*-hexane and 143 for acetone; the resolution of each photo is 1280×960 pixels).

n-hexane iSolv_meas.	*acetone* iSolv_meas.	Analyzed solution	iSolv1	iSolv2	iSolv⁻	iSolv_ref.	**Δ1**	**Δ2**	**Δ⁻**
39.99	141.86	3_methanol	140.62	137.58	139.10	135	**-5.62**	**-2.58**	**-4.1**
39.53	142.37	4_benzene	92.49	92.72	92.61	88	**-4.49**	**-4.72**	**-4.605**
37.71	143.51	5_diethyl ether	130.29	127.08	128.69	126	**-4.29**	**-1.08**	**-2.685**
36.57	143.01	6_DCM	74.51	76.77	75.64	82	**7.49**	**5.23**	**6.36**
37.47	143.21	7_cyclohexane	41.80	47.25	44.53	47	**5.2**	**-0.25**	**2.475**

Table S19. iSolv values for photographs of standard solutions (*n*-hexane and acetone) and analyzed solutions obtained under laboratory lighting conditions using a mobile phone camera 9 (each photograph contains three vials with a solution, circle radius 30 pixels, reference iSolv values are 48 for *n*-hexane and 143 for acetone; the resolution of each photo is 1280×960 pixels).

n-hexane iSolv_meas.	*acetone* iSolv_meas.	Analyzed solution	iSolv1	iSolv2	iSolv⁻	iSolv_ref.	**Δ1**	**Δ2**	**Δ⁻**
51.32	136.89	3_methanol	132.24	136.28	134.26	135	**2.76**	**-1.28**	**0.74**
48.57	138.24	4_benzene	96.05	96.09	96.07	88	**-8.05**	**-8.09**	**-8.07**
51.31	137.20	5_diethyl ether	122.68	125.56	124.12	126	**3.32**	**0.44**	**1.88**
49.66	138.11	6_DCM	87.50	89.61	88.56	82	**-5.5**	**-7.61**	**-6.555**
48.88	138.43	7_cyclohexane	50.23	47.48	48.86	47	**-3.23**	**-0.48**	**-1.855**

Table S20. iSolv values for photographs of standard solutions (*n*-hexane and acetone) and analyzed solutions obtained under laboratory lighting conditions using a mobile phone camera 10 (each photograph contains three vials with a solution, circle radius 30 pixels, reference iSolv values are 48 for *n*-hexane and 143 for acetone; the resolution of each photo is 1280×960 pixels).

n-hexane iSolv_meas.	*acetone* iSolv_meas.	Analyzed solution	iSolv1	iSolv2	iSolv⁻	iSolv_ref.	**Δ1**	**Δ2**	**Δ⁻**
51.86	134.66	3_methanol	128.49	133.35	130.92	135	**6.51**	**1.65**	**4.08**
51.58	134.63	4_benzene	92.94	92.57	92.76	88	**-4.94**	**-4.57**	**-4.755**
50.22	134.24	5_diethyl ether	118.69	121.72	120.21	126	**7.31**	**4.28**	**5.795**
51.20	134.42	6_DCM	81.43	79.44	80.44	82	**0.57**	**2.56**	**1.565**
41.97	142.31	7_cyclohexane	43.84	46.59	45.22	47	**3.16**	**0.41**	**1.785**

Table S21. iSolv values for five different solvents obtained using 10 cameras of various mobile phones (σ – standard deviation for the iSolv values of each solvent).

Camera №	3_methanol	4_benzene	5_diethyl ether	6_DCM	7_cyclohexane
1	134	89	122	73	49
2	137	94	126	78	47
3	138	95	129	81	47
4	137	97	129	81	46
5	134	91	123	77	49
6	137	92	128	75	46
7	138	97	124	81	44
8	139	93	129	76	45
9	134	96	124	89	49
10	131	93	120	80	45
**σ**	**2.5**	**2.6**	**3.3**	**4.5**	**1.8**

7. Computational Details

Studying the molecular and electronic structure of bimolecular complexes of the I_2 molecule with solvent molecules.

The molecular structures of the $I_2 \cdot C_6H_{12}$, $I_2 \cdot CH_2Cl_2$, and $I_2 \cdot$MeOH complexes were optimized using the PBE0[15,16] method with the def2-TZVP(ZORA)[17,18] basis set and the auxiliary basis set SARC/J.[19,20] Empirical corrections D4 were employed to better describe dispersion interactions.[21,22] Calculations at this level of theory were performed using the Orca 5.0.3 software package.[23,24] Visualization of the molecular electrostatic potential was performed by the GaussView 6.0 software package (electron density threshold was 0.008 a.u.).[25]

DFT molecular dynamics of the I_2 molecule under microsolvation conditions.

In the case of cyclohexane, the microsolvation complex consisted of one iodine molecule and 6 cyclohexane molecules. For the dichloromethane and methanol media, the iodine molecule was solvated with 10 solvent molecules. In the first stage, the solvate complexes were optimized using the PBE0 method with the LANL2DZ[26,27,28] basis set for iodine atoms and the def2-SVP[17] basis set for all other atoms. Empirical D3 corrections were used for dispersion interactions.[29] In the next step, the optimized structure was used for molecular dynamics simulation at the same theoretical level. MD simulations were performed using the NVT ensemble (T = 298 K, Langevin thermostat, time step of 1 fs). To prevent dissociation of the microsolvation complex, a spherical confining potential was applied. Optimization of the structure and MD simulations of the microsolvation complexes were performed by the TeraChem software package.[30,31] Visualization and analysis of the MD simulation data were carried out using the VMD program.[32]

Modeling of the I_2 solutions by DFT molecular dynamics with periodic boundary conditions.

The model of the I_2 solution in cyclohexane consisted of a system containing one iodine molecule and 30 C_6H_{12} molecules in a periodic cell. Models of I_2 solutions in DCM and MeOH each contained one iodine molecule and 40 solvent molecules. In the first stage, the molecular structures of the solutions were optimized using the PBE DZVP D3BJ method.[33] Using the optimized structures as a starting point, MD simulations of the solutions were subsequently performed in the NPT-I ensemble by the same method (T = 298 K, p = 1 atm), which yielded average volumes of the periodic cell for each solvent along the trajectory. All DFT molecular dynamics calculations employing PBC were carried out by the CP2K 2023.2 software package.[34]

8. Materials and methods

Preparation of iodine solutions in various solvents.

Into clear 12-mL vials, 2.5 mg of crystalline iodine was added, followed by the addition of 10 mL of each solvent (resulting in an approximate solution concentration of 10^{-3} mol/L). Iodine dissolved rapidly and completely within a few minutes in most solvents used. For solvents with low iodine solubility (e.g., water), an ultrasonic bath was employed to facilitate dissolution. The samples were sonicated for 30–60 minutes at room temperature until complete visual dissolution of iodine crystals was achieved. A total of 36 iodine samples were prepared in molecular solvents. The formamide sample did not provide a color suitable for quantitative iSolv assignment and was therefore excluded from the dataset (Table S1; Figure 2b of the main text).

Sample preparation for color classification study

Into clear 2-mL vials, 0.4 mg of crystalline iodine was added, followed by the addition of 1.5 mL of corresponding solvent (resulting in an approximate solution concentration of 10^{-3} mol/L). For *n*-hexane, chloroform, benzene, 1,2-dichloroethane, *p*-xylene, diethyl ether, nitromethane and 1,2-

dichlorobenzene duplicate solutions were made to study the reproducibility of the color of the iodine solutions in these solvents. Combined with fourteen new solvents, this approach yielded a final set of fifty iodine solutions for the color categorization experiment.

Photography of iodine solutions.

To determine the color of the iodine solutions, photographs were taken under controlled lighting conditions immediately after their preparation to prevent any potential time-dependent color changes. Each solution was placed in a transparent cylindrical vial with a diameter of 18.5 mm. Each vial was individually photographed against a white background paper. Photography was performed by a Nikon D610 camera equipped with an AF-S Micro Nikkor 60 mm lens (f/5.6; shutter speed 1/200 s, ISO 200). For sample illumination, a continuous LED light source (GreenBean StudioLight II 200 bicolor) was positioned above the sample at a distance of 45 cm. The illuminated area of the light source was 455×265 mm, ensuring uniform illumination from all sides, which was enhanced by a diffusing filter. The total power output of the lighting setup during shooting was 100 W. The lighting device allowed precise adjustment of the color temperature, which was maintained at 5600 K during the shoot, corresponding to cool white light. The Color Rendering Index (CRI) of this LED light source exceeds 95. Since color heterogeneities exist when the solutions are photographed in a cylindrical glass container, the final color value of the solution was obtained by averaging the color in a rectangular area located centrally within the colored solution in the photograph (Figure 2).

9. XYZ coordinates and energies of the optimized molecular structures

Table S22. Atomic coordinates of optimized structures (Å), total SCF energies (E, a.u.), enthalpies (H, a.u.), and Gibbs free energies (G, a.u.) for individual solvent molecules, intermolecular complexes of an iodine molecule with one solvent molecule $[I_2]\cdot[Solvent]_1$, and intermolecular complexes of an iodine molecule with two solvent molecules $[I_2]\cdot[Solvent]_2$. Structure optimization and thermodynamic parameter calculations were performed using the PBE0 D4 RIJCOSX ZORA-def2-TZVP SARC/J method.

I_2 and individual solvent molecules

I_2
E = -14447.67635852
H = -14447.67201550
G = -14447.70153083

I	-0.051800	-1.248617	0.000000
I	-0.051800	1.413117	0.000000

Cyclohexane
E = -235.83374122
H = -235.65657336
G = -235.69200483

C	-1.107578	-0.682104	-0.651407
C	-1.133771	0.839647	-0.606602
C	0.318611	-1.214128	-0.605740
C	-0.381046	1.371106	0.605771
C	1.045144	0.839080	0.651440
C	1.071335	-0.682671	0.606636
H	-1.665082	-1.076020	0.207969
H	-1.619948	-1.045221	-1.547351
H	-0.668616	1.233345	-1.519273
H	-2.166018	1.202224	-0.602418
H	0.316401	-2.308217	-0.602693
H	0.845358	-0.905792	-1.517857
H	-0.378827	2.465196	0.602721
H	-0.907784	1.062780	1.517895
H	1.602650	1.232995	-0.207936
H	1.557512	1.202195	1.547386
H	2.103581	-1.045251	0.602448
H	0.606179	-1.076363	1.519310

Iso-octane
E = -315.63991590
H = -315.38346690
G = -315.42942921

C	-0.401798	2.070433	-1.184431
H	-0.747941	3.103196	-1.090030
H	0.685444	2.084997	-1.102943
H	-0.657198	1.726187	-2.191555
C	0.492024	-2.378470	0.067862
C	0.623860	-0.873636	-0.167662
C	-0.803202	-0.306644	-0.292719
C	-1.055635	1.192584	-0.126092
C	-2.559584	1.438914	-0.112373
C	1.359392	-0.253048	1.015597
C	1.440486	-0.677097	-1.444443
H	1.475243	-2.855071	0.112633
H	-0.027843	-2.587436	1.006909
H	-0.072719	-2.852461	-0.740340
H	-1.215650	-0.626248	-1.259365
H	-1.413339	-0.809659	0.467570
H	-2.786500	2.492808	0.067151
H	-3.005437	1.162667	-1.073296
H	-3.054282	0.849727	0.663912
H	1.547463	0.812340	0.863520
H	0.784660	-0.364075	1.939750
H	2.326777	-0.740865	1.164701
H	1.637068	0.372403	-1.661017
H	2.407473	-1.180812	-1.355904
H	0.921566	-1.105098	-2.306844
H	-0.664729	1.495565	0.852909

n-Hexane
E = -237.02840032
H = -236.83002524
G = -236.87084580

C	1.422714	-2.743956	0.000001
C	1.386654	-1.226066	-0.000001
C	-0.025596	-0.665119	-0.000002
C	-0.074831	0.852471	-0.000001
C	-1.487083	1.413416	0.000000
C	-1.523147	2.931306	0.000001
H	2.446908	-3.123657	0.000002
H	0.917768	-3.149022	0.881509
H	0.917768	-3.149025	-0.881506
H	1.925039	-0.843981	0.875088
H	1.925040	-0.843982	-0.875090
H	-0.566238	-1.046310	-0.875654
H	-0.566240	-1.046311	0.875648
H	0.465812	1.233662	0.875651
H	0.465811	1.233663	-0.875652
H	-2.025467	1.031329	-0.875088
H	-2.025465	1.031329	0.875090
H	-2.547343	3.311003	0.000001
H	-1.018202	3.336374	0.881508
H	-1.018203	3.336375	-0.881507

CCl_4
E = -1887.37726988
H = -1887.36111111
G = -1887.39859714

C	0.038311	-0.039598	0.000000
Cl	0.037965	1.722942	-0.000000
Cl	1.700104	-0.626807	-0.000000
Cl	-0.793040	-0.626868	1.438812
Cl	-0.793040	-0.626868	-1.438812

Chloroform
E = -1425.66431321
H = -1425.63891652
G = -1425.67349505

C	0.029925	-0.110058	-0.000000
Cl	1.709946	-0.630974	0.000000
Cl	-0.803815	-0.653105	1.450216
Cl	-0.803816	-0.653105	-1.450216
H	0.019960	0.974441	-0.000000

Chlorobenzene
E = -693.93856514
H = -693.84081892
G = -693.87700921

C	-0.329225	-0.144197	0.000000
C	-0.343876	1.241241	0.000000
C	0.863259	-0.849297	0.000000
C	2.059784	-0.149813	0.000000
C	2.063028	1.236820	-0.000000
C	0.860513	1.927309	0.000000
Cl	-1.825534	-1.009708	-0.000001
H	-1.288252	1.770407	0.000001
H	0.848527	-1.931679	0.000000
H	2.995170	-0.697136	0.000000
H	3.001237	1.778465	-0.000001
H	0.854669	3.011088	0.000000

DCM
E = -963.94496396
H = -963.91097040
G = -963.94227226

C	0.882524	0.989868	0.016115
H	0.863749	2.075381	0.016084
H	1.899728	0.610614	0.016120
Cl	0.086911	0.427564	1.484282
Cl	0.086887	0.427473	-1.452001

Benzene
E = -232.20841509
H = -232.10240872
G = -232.13390912

C	-1.190894	-0.764065	0.000000
C	-1.190898	0.623154	0.000000
C	0.010529	-1.457580	-0.000000
C	1.211961	-0.764062	0.000000
C	1.211964	0.623150	-0.000000
C	0.010529	1.316661	-0.000000
H	-2.129717	-1.306176	-0.000000
H	-2.129724	1.165256	0.000000
H	0.010539	-2.541671	-0.000000
H	2.150782	-1.306176	0.000000
H	2.150790	1.165255	-0.000000
H	0.010539	2.400753	-0.000000

2-Chlorophenol
E = -769.21149691
H = -769.10813952
G = -769.14662211

C	-0.783507	0.374346	0.077825
C	-0.773059	1.763478	0.040766
C	0.436890	-0.299309	0.062654
C	1.637686	0.384925	0.012512
C	1.633849	1.768855	-0.024049
C	0.425314	2.451435	-0.009682
O	-1.965565	-0.265051	0.126538
Cl	0.412169	-2.031684	0.108129
H	-1.723486	2.282879	0.052391
H	2.565465	-0.173201	0.002502
H	2.571595	2.308597	-0.063733
H	0.414401	3.534510	-0.038385
H	-1.796350	-1.216279	0.148531

2,2,2-Trifluoroethanol
E = -453.09332990
H = -453.02887718
G = -453.06596832

C	-4.096083	1.437062	8.524144
C	-3.987675	2.887189	8.932884
O	-3.133915	3.529664	8.032030
F	-4.909720	0.798970	9.378910
F	-4.603145	1.293930	7.299029
F	-2.913515	0.820422	8.548959
H	-5.004991	3.296723	8.925640
H	-3.609642	2.897452	9.962342
H	-3.055813	4.451589	8.285061

Toluene
E = -271.51873338
H = -271.38451278
G = -271.41976258

C	-0.927417	-0.443811	0.000000
C	-0.908211	0.949282	0.000001
C	0.288443	-1.117592	0.000000
C	1.490682	-0.424634	0.000000
C	1.495212	0.960474	-0.000000
C	0.288434	1.645939	-0.000000
C	-2.227450	-1.188550	-0.000000
H	-1.847040	1.494369	0.000001
H	0.293821	-2.202817	0.000000
H	2.426874	-0.971505	0.000000
H	2.432556	1.504560	-0.000001
H	0.280309	2.730174	-0.000000
H	-2.066795	-2.267444	-0.000000
H	-2.826510	-0.938223	0.879720
H	-2.826509	-0.938223	-0.879721

1,2-DCE
E = -1003.25818128
H = -1003.19405194
G = -1003.22814002

C	0.132631	-0.790572	-0.000000
C	0.156944	0.715198	-0.000000
Cl	1.804923	-1.404765	0.000001

2-Bromoanisole
E = -2970.26503570
H = -2970.13237882
G = -2970.17558658

O	-1.671525	0.031276	0.000223
C	-2.982515	0.543123	-0.005000
H	-3.642039	-0.322316	-0.004534

Cl	-1.515348	1.329389	0.000001
H	-0.361543	-1.179015	0.888563
H	-0.361542	-1.179015	-0.888564
H	0.651118	1.103640	0.888563
H	0.651117	1.103640	-0.888564

H	-3.179703	1.148640	0.885753
H	-3.174486	1.143708	-0.900225
C	-0.652096	0.907951	-0.000454
C	-0.805181	2.291058	-0.004824
C	0.643776	0.382700	0.003555
C	1.747099	1.211930	0.002939
C	1.582722	2.589934	-0.001484
C	0.306299	3.121485	-0.005283
Br	0.882292	-1.483809	0.009736
H	-1.795523	2.726071	-0.007938
H	2.736406	0.772137	0.005935
H	2.451606	3.236160	-0.001913
H	0.162667	4.195549	-0.008584

3-Bromoanisole
E = -2970.26722968
H = -2970.13464272
G = -2970.17789388

O	-1.640080	0.022465	0.013605
C	-2.955745	0.522585	-0.005563
H	-3.610258	-0.346791	0.000164
H	-3.163803	1.136777	0.876826
H	-3.146839	1.112215	-0.908382
C	-0.627445	0.914953	0.012208
C	-0.793921	2.295548	-0.004980
C	0.654560	0.366746	0.029101
C	1.744013	1.209794	0.028304
C	1.606276	2.590243	0.012129
C	0.327114	3.114927	-0.004456
H	-1.779713	2.739080	-0.019085
H	2.477213	3.231220	0.012050
H	0.194801	4.190691	-0.017906
H	0.769461	-0.708806	0.041640
Br	3.476566	0.458652	0.048448

p-Xylene
E = -310.82887372
H = -310.66545765
G = -310.70532032

C	-1.224327	-0.748881	-0.000001
C	-1.196202	0.644587	-0.000006
C	-0.008556	-1.417931	0.000004
C	0.000985	1.335740	-0.000007
C	1.221948	0.663689	-0.000002
C	1.193740	-0.723820	0.000003
C	-2.525870	-1.490608	0.000001
C	2.515279	1.419924	0.000001
H	-2.131266	1.196372	-0.000011
H	0.001942	-2.503251	0.000008
H	-0.009419	2.421437	-0.000012
H	2.128373	-1.275613	0.000007
H	-2.366503	-2.569807	-0.000007
H	-3.125207	-1.240507	0.879753
H	-3.125217	-1.240496	-0.879741
H	3.369765	0.741737	-0.000024
H	2.598258	2.063934	-0.879791
H	2.598278	2.063892	0.879822

4-Bromoanisole
E = -2970.26673718
H = -2970.13406842
G = -2970.17723321

O	-1.644717	0.014383	0.021278
C	-2.956423	0.521624	-0.024251
H	-3.616506	-0.343396	-0.043267
H	-3.183187	1.128135	0.859241
H	-3.122706	1.122130	-0.925001
C	-0.627177	0.903090	0.026774
C	-0.789203	2.283728	0.018505
C	0.656872	0.361509	0.044644
C	1.763682	1.185281	0.054757
C	1.591918	2.562151	0.046524
C	0.326078	3.111688	0.028455
H	-1.773314	2.732019	0.007277
H	0.199486	4.186765	0.023571
H	0.766758	-0.716039	0.051948
H	2.759124	0.760308	0.069159
Br	3.103813	3.693123	0.061087

2-Bromopropane
E = -2742.61166261
H = -2742.51113679
G = -2742.54679060

C	-0.064564	-1.045701	-1.004639
C	-0.118592	0.461624	-1.016685
C	-1.521652	1.015027	-1.004764
Br	0.860469	1.155641	0.544037
H	0.962229	-1.411361	-1.007918
H	-0.571897	-1.445532	-0.124373
H	-0.571349	-1.431157	-1.895419
H	0.447872	0.861717	-1.857498
H	-1.523740	2.104998	-1.006549
H	-2.053738	0.666873	-1.896080
H	-2.067836	0.669273	-0.124912

Nitrobenzene
E = -436.82182239
H = -436.71027677
G = -436.74938803

C	-0.094016	0.053537	0.000000
C	-0.122637	1.436669	0.000000
C	1.089631	-0.662700	0.000000
C	2.284392	0.036332	-0.000000
C	2.279887	1.424266	0.000000
C	1.080015	2.121883	0.000000
N	-1.364912	-0.679664	0.000000
O	-1.312838	-1.890430	0.000001
O	-2.387300	-0.028794	-0.000002
H	-1.074626	1.949177	0.000000
H	1.057815	-1.743397	-0.000000
H	3.222589	-0.505025	-0.000001

Diethyl ether
E = -233.66693143
H = -233.52253733
G = -233.56018451

O	-0.029275	-0.009197	-0.000001
C	-0.070496	1.394652	0.000000
C	-1.510653	1.838447	-0.000000
C	1.280546	-0.516116	-0.000000
C	1.218865	-2.021769	0.000000
H	0.455735	1.785818	0.885140
H	0.455735	1.785819	-0.885139
H	-1.574551	2.928649	-0.000000
H	-2.027343	1.461277	-0.884543
H	-2.027343	1.461277	0.884543
H	1.824693	-0.150389	-0.885104
H	1.824692	-0.150389	0.885103

H	3.218350	1.966299	0.000000
H	1.079947	3.205047	0.000000

H	2.225478	-2.445151	-0.000000
H	0.691159	-2.383363	0.884533
H	0.691159	-2.383364	-0.884533

Ethyl acetate
E = -307.76119809
H = -307.63457455
G = -307.67423087

C	0.527649	-2.148830	0.000000
C	0.778910	-0.672118	0.000003
O	-0.374027	0.011125	-0.000012
O	1.860821	-0.149794	0.000017
C	-0.252540	1.438141	-0.000006
C	-1.641713	2.015781	0.000001
H	1.476624	-2.678544	-0.000001
H	-0.056541	-2.426381	0.879171
H	-0.056544	-2.426379	-0.879169
H	0.317957	1.743977	0.880341
H	0.317952	1.743984	-0.880354
H	-1.587984	3.106376	0.000004
H	-2.196985	1.700483	-0.885232
H	-2.196978	1.700478	0.885237

Acetonitrile
E = -132.74431021
H = -132.69437529
G = -132.72188151

C	-0.437943	-1.207063	0.000005
C	-0.437882	0.242018	-0.000008
N	-0.437938	1.390850	-0.000021
H	0.586467	-1.581964	-0.000021
H	-0.950127	-1.581912	0.887221
H	-0.950177	-1.581928	-0.887176

1,4-Dioxane
E = -307.71654712
H = -307.58763694
G = -307.62211759

C	-1.274083	-0.756679	-0.616621
C	-0.559689	-1.261666	0.616319
O	-1.262148	0.653702	-0.658856
O	0.765920	-0.780288	0.658857
C	0.063461	1.135080	-0.616318
C	0.777855	0.630093	0.616620
H	-2.320913	-1.068034	-0.614346
H	-0.786801	-1.162451	-1.516182
H	-0.504338	-2.352414	0.614489
H	-1.104872	-0.937059	1.515594
H	0.608643	0.810474	-1.515594
H	0.008110	2.225829	-0.614489
H	0.290573	1.035866	1.516182
H	1.824685	0.941448	0.614346

Acetic acid
E = -229.15958849
H = -229.09296084
G = -229.12365438

C	0.021347	-1.391689	-0.000002
C	-0.062657	0.102948	-0.000007
O	-1.081883	0.736906	-0.000029
O	1.150294	0.684671	0.000019
H	1.049660	-1.745056	-0.000020
H	-0.502890	-1.769298	0.878853
H	-0.502929	-1.769308	-0.878828
H	0.994458	1.640026	0.000014

2-Propanol
E = -194.37422633
H = -194.25972985
G = -194.29353451

C	0.513251	-0.996905	0.046985
C	0.524699	0.519139	0.078293
C	-0.860534	1.106389	-0.059897
H	1.526098	-1.400628	0.138563
H	-0.079050	-1.401243	0.871586
H	0.086680	-1.351490	-0.894625
H	0.953326	0.850394	1.036907
H	-0.815244	2.196489	-0.048969
H	-1.308637	0.791043	-1.005343
H	-1.503735	0.776209	0.758246
O	1.296339	1.048206	-0.990135
H	2.174509	0.661396	-0.947709

Ethanol
E = -155.06258227
H = -154.97722022
G = -155.00781141

C	0.739379	-0.673411	0.398407
O	0.801402	0.738023	0.435120
H	1.777926	-1.011597	0.421190
H	0.253079	-1.065859	1.302541
H	-0.096611	1.077706	0.406308
C	0.043603	-1.198874	-0.837903
H	0.054194	-2.291852	-0.854920
H	-1.002559	-0.878386	-0.866134
H	0.536188	-0.833151	-1.741109

Nitromethane
E = -245.10562377
H = -245.05106589
G = -245.08121620

C	0.476592	-1.437929	0.000007
N	0.471741	0.047367	-0.000007
O	-0.612811	0.582733	-0.000005
O	1.541228	0.611449	-0.000022
H	1.505529	-1.781101	0.000015
H	-0.062395	-1.761253	0.888113
H	-0.062384	-1.761267	-0.888101

THF
E = -232.46286050
H = -232.33993574
G = -232.37362437

C	-1.097406	-0.613137	0.175988
C	1.144247	-0.003657	0.073896
O	0.234742	-1.091730	0.048264
H	-1.631979	-0.788829	-0.766254
H	-1.605327	-1.176450	0.964059
H	1.626778	0.054769	1.059704
H	1.920339	-0.181851	-0.674616
C	-0.996315	0.874365	0.482145
C	0.317125	1.240257	-0.196998
H	-0.921449	1.038965	1.560631

H	-1.853079	1.439164	0.112363
H	0.774287	2.148874	0.197275
H	0.167736	1.370359	-1.272559

Methanol
E = -115.75142294
H = -115.69573195
G = -115.72277146

C	0.165028	-0.734261	-0.000001
O	0.200789	0.674480	-0.000006
H	1.199098	-1.080017	-0.000026
H	-0.329607	-1.139845	0.891218
H	-0.329651	-1.139851	-0.891192
H	-0.701057	1.001094	0.000007

Water
E = -76.46477324
H = -76.43951919
G = -76.46093489

O	-0.122639	0.089330	0.000000
H	-0.085165	1.048173	0.000000
H	0.793904	-0.194903	0.000000

Dimethylacetamide
E = -287.85146967
H = -287.71248615
G = -287.75236823

N	0.498472	0.397218	-0.000003
C	0.527541	1.838158	0.000001
C	1.783559	-0.247022	-0.000001
C	-0.703322	-0.246407	0.000002
C	-0.683912	-1.757074	-0.000002
O	-1.759592	0.359595	0.000007
H	-0.495249	2.205940	0.000002
H	1.049747	2.212239	0.887099
H	1.049747	2.212243	-0.887095
H	1.684760	-1.328371	-0.000016
H	2.361920	0.043044	-0.884849
H	2.361908	0.043022	0.884862
H	-1.718046	-2.091228	0.000001
H	-0.180619	-2.156177	-0.883496
H	-0.180613	-2.156182	0.883486

1-Butanol
E = -233.67432988
H = -233.52925179
G = -233.56707890

C	0.952846	-2.297777	0.000001
C	0.930707	-0.779490	-0.000004
C	-0.479672	-0.215076	-0.000007
C	-0.503198	1.293748	0.000003
O	-1.851277	1.720284	0.000001
H	1.973950	-2.685227	0.000002
H	0.444217	-2.697447	0.881484
H	0.444217	-2.697454	-0.881479
H	1.473007	-0.404631	0.875744
H	1.473009	-0.404636	-0.875753
H	-1.027639	-0.574078	-0.877843
H	-1.027648	-0.574089	0.877819
H	0.027467	1.671258	0.886421
H	0.027473	1.671270	-0.886406
H	-1.869159	2.679544	0.000015

DMSO
E = -554.85142007
H = -554.76540969
G = -554.80012804

S	-0.216509	-0.014861	-0.308031
C	-0.010555	1.772270	-0.281799
C	1.537111	-0.416802	-0.282472
O	-0.759129	-0.401168	1.015591
H	-1.011005	2.203226	-0.249915
H	0.543678	2.053149	0.615312
H	0.505484	2.102010	-1.185145
H	1.609361	-1.503650	-0.248443
H	2.020743	-0.041938	-1.186068
H	1.986721	0.013064	0.614172

DMF
E = -248.53481355
H = -248.42711754
G = -248.46089515

C	-0.016845	-0.902152	-0.000000
N	-0.058794	0.444983	0.000001
C	-1.329077	1.137171	0.000000
C	1.198888	1.159722	0.000000
O	1.000881	-1.560183	-0.000003
H	-1.024852	-1.360239	0.000001
H	-1.148975	2.210452	0.000000
H	-1.922171	0.900010	-0.887817
H	-1.922172	0.900010	0.887817
H	1.004442	2.230635	-0.000001
H	1.791887	0.902797	0.880956
H	1.791888	0.902794	-0.880954

Acetone
E = -193.17178110
H = -193.08275830
G = -193.11509966

C	0.120078	-1.359248	-0.000022
C	0.168544	0.145911	0.000001
C	-1.159217	0.856890	0.000018
O	1.213238	0.747557	0.000008
H	1.129528	-1.765549	0.000135
H	-0.424304	-1.719173	0.877621
H	-0.423994	-1.719126	-0.877877
H	-1.007791	1.934452	-0.000160
H	-1.743399	0.565201	-0.877418
H	-1.743184	0.565484	0.877693

NMP
E = -325.96706775
H = -325.81989842
G = -325.85856229

C	-1.746645	-0.723629	0.863065
H	-1.912088	-0.480999	1.914898
H	-2.569751	-1.358069	0.534997
C	-0.374021	-1.360115	0.685954
C	-1.656653	0.569984	0.050812
C	0.543016	-0.196481	0.363146
N	-0.246725	0.870374	0.060963
O	1.754449	-0.205277	0.347495
C	0.279351	2.097804	-0.455981
H	-0.340592	-2.047570	-0.165298
H	-0.009118	-1.902883	1.556813
H	-2.232402	1.386708	0.496088
H	-2.019906	0.434596	-0.978324
H	-0.095709	2.954254	0.111891
H	1.365019	2.057512	-0.373633
H	0.006876	2.235589	-1.508685

Cyclopentane
E = -196.51754320
H = -196.37049572
G = -196.40485294

C	-0.951358	-0.723656	0.251110
C	-0.958924	0.803022	0.249801
C	0.257709	-1.060380	-0.611782
C	0.527700	1.209215	0.247791
C	1.313748	-0.061332	-0.139524
H	-1.884315	-1.157413	-0.115647
H	-0.792442	-1.099189	1.268300
H	-1.511068	1.227278	1.090779
H	-1.445919	1.160310	-0.662567
H	0.014159	-0.883475	-1.665343
H	0.582723	-2.099283	-0.522409
H	0.839131	1.572728	1.229429
H	0.709980	2.024287	-0.455588
H	1.829017	-0.464762	0.736864
H	2.078860	0.131350	-0.894113

1,2-Dichlorobenzene
E = -1155.66490417
H = -1155.57545850
G = -1155.61419126

C	-0.627899	0.684753	-0.000001
C	-0.622000	2.072292	0.000000
C	0.577789	-0.011413	0.000000
C	1.776424	0.687463	-0.000001
C	1.776581	2.070798	-0.000001
C	0.575858	2.764100	0.000000
Cl	-2.131547	-0.144898	-0.000004
Cl	0.611028	-1.728430	0.000004
H	-1.568791	2.597156	0.000001
H	2.704396	0.130009	-0.000000
H	2.718726	2.605243	-0.000002
H	0.567637	3.847228	0.000003

1-Chloro-2-methylpropane
E = -620.14641454
H = -620.01562592
G = -620.05333920

C	-0.183305	-2.258124	-0.669535
C	-0.239910	-0.735649	-0.651108
C	-1.674886	-0.237722	-0.682582
C	0.511491	-0.238132	0.568235
Cl	0.689590	1.539514	0.595998
H	0.845954	-2.624868	-0.687622
H	-0.676267	-2.678468	0.212471
H	-0.695098	-2.650686	-1.550746
H	0.283793	-0.356737	-1.536288
H	-1.719191	0.852190	-0.669741
H	-2.185462	-0.589181	-1.581793
H	-2.231066	-0.609804	0.183814
H	-0.006992	-0.512292	1.488877
H	1.522449	-0.643942	0.600119

m-Xylene
E = -310.82900951
H = -310.66654849
G = -310.70421816

C	-2.472434	1.134867	0.000879
H	-2.495612	1.879278	-0.798879
H	-3.328534	0.471600	-0.130101
H	-2.607096	1.670867	0.944840
C	-1.174478	-1.021542	-0.003239
C	0.024559	-1.715471	0.001730
C	-1.184084	0.369369	-0.005834
C	0.034363	1.039104	-0.005597
C	1.248217	0.360607	-0.000268
C	1.228691	-1.029961	0.003630
C	2.541737	1.117416	0.000048
H	-2.114561	-1.563204	-0.006423
H	0.020761	-2.799783	0.002473
H	0.038254	2.126236	-0.010710
H	2.164920	-1.578231	0.006235
H	3.396950	0.440304	-0.003295
H	2.621726	1.764401	-0.877656
H	2.624422	1.758543	0.881869

1-Bromo-2-methylpropane
E = -2781.91591404
H = -2781.78558525
G = -2781.82456893

C	0.702990	-2.206066	-1.117728
C	0.641523	-0.682251	-1.096875
C	-0.796359	-0.193204	-1.130498
C	1.390319	-0.192114	0.124835
Br	1.613395	1.743760	0.159754
H	1.733398	-2.569137	-1.134182
H	0.209161	-2.629099	-0.237670
H	0.194256	-2.597395	-2.001377
H	1.163785	-0.302909	-1.982381
H	-0.848362	0.896526	-1.122417
H	-1.303823	-0.551833	-2.028540
H	-1.350403	-0.565364	-0.262901
H	0.866624	-0.442774	1.047613
H	2.401795	-0.592139	0.164866

1-Bromopropane
E = -2742.60793103
H = -2742.50687161
G = -2742.54292866

Br	-0.998830	-0.806922	0.000153
C	-0.944444	1.139224	-0.000021
C	0.471074	1.660907	-0.000016
C	0.491696	3.182248	0.000004
H	-1.495709	1.446695	-0.887535
H	-1.495758	1.446843	0.887411
H	0.997909	1.275300	0.876861
H	0.997914	1.275308	-0.876895
H	1.517357	3.555946	0.000004
H	-0.009763	3.587194	-0.882865
H	-0.009745	3.587157	0.882900

Propylene carbonate
E = -381.83062740
H = -381.72012915
G = -381.75748098

C	-0.807077	-0.333797	-0.262763
C	-0.881436	1.186943	-0.216487
C	1.054212	0.512422	0.734494
O	0.548633	-0.577132	0.124048
O	0.190300	1.546157	0.642605
O	2.107130	0.560526	1.275247
C	-1.090545	-0.948644	-1.604636

Acetophenone
E = -384.88864551
H = -384.74170482
G = -384.78280944

C	0.186518	-1.338636	-0.000002
C	1.376194	-2.041813	-0.000031
C	0.191056	0.054895	0.000031
C	2.586203	-1.359377	-0.000014
C	2.601118	0.026873	0.000027
C	1.407954	0.731580	0.000047
C	-1.119979	0.766818	0.000030

H	-1.449080	-0.775923	0.507180
H	-0.715538	1.633213	-1.202338
H	-1.813851	1.563891	0.202088
H	-0.419366	-0.540115	-2.362640
H	-0.960604	-2.030684	-1.569334
H	-2.121278	-0.738358	-1.900365

C	-1.119892	2.272205	-0.000035
O	-2.159161	0.146929	0.000088
H	-0.770111	-1.846922	-0.000006
H	1.365113	-3.125591	-0.000058
H	3.519696	-1.910594	-0.000037
H	3.544397	0.560461	0.000033
H	1.430966	1.814550	0.000065
H	-2.149151	2.623937	-0.000015
H	-0.600636	2.659574	0.880302
H	-0.600687	2.659511	-0.880426

Isobutanol
E = -233.67648417
H = -233.53198383
G = -233.56935997

C	0.629439	-1.815232	-0.270305
C	0.626723	-0.295837	-0.215035
C	-0.797784	0.220164	-0.248256
C	1.371420	0.218770	1.006370
O	-0.776394	1.632004	-0.323086
H	1.650455	-2.202881	-0.277887
H	0.123412	-2.238509	0.603288
H	0.122959	-2.188583	-1.164274
H	1.124129	0.089513	-1.113716
H	-1.320528	-0.207569	-1.115603
H	-1.324177	-0.117315	0.658084
H	1.350190	1.308216	1.048373
H	0.914720	-0.164957	1.924896
H	2.414776	-0.105840	0.993774
H	-1.679841	1.953456	-0.302623

Pyridine
E = -248.26190375
H = -248.16757510
G = -248.20018240

C	-0.940545	-0.656383	0.000000
C	-0.938613	0.728444	-0.000001
C	0.275190	-1.319387	0.000000
C	1.438856	-0.564518	-0.000000
C	0.282101	1.387009	-0.000000
N	1.455015	0.763690	0.000001
H	-1.873197	-1.209123	0.000001
H	-1.863339	1.292797	-0.000001
H	0.326250	-2.401507	0.000001
H	2.408806	-1.054536	-0.000001
H	0.317875	2.473114	0.000001

Intermolecular complexes of an iodine molecule with one solvent molecule $[I_2]\cdot[Solvent]_1$

$[I_2]\cdot[Cyclohexane]_1$
E = -14683.51334536
H = -14683.33054070
G = -14683.38174449

C	-1.042775	-0.668008	-0.720461
C	-0.804619	0.830585	-0.847466
C	0.216635	-1.392339	-0.265019
C	-0.269204	1.418826	0.450521
C	0.991293	0.697107	0.907364
C	0.752058	-0.801407	1.031526
H	-1.843722	-0.845029	0.008577
H	-1.392599	-1.079752	-1.671680
H	-0.078918	1.012574	-1.650199
H	-1.727618	1.338613	-1.142122
H	0.019186	-2.461329	-0.142695
H	0.982089	-1.305406	-1.046603
H	-0.069080	2.487578	0.330233
H	-1.037059	1.331612	1.229755
H	1.791030	0.877945	0.177925
H	1.342121	1.106601	1.859180
H	1.671919	-1.316894	1.338642
H	0.030315	-0.986092	1.837085
I	6.574108	-1.628144	-1.284800
I	4.186440	-1.423541	-0.115164

$[I_2]\cdot[Iso\text{-}octane]_1$
E = -14763.31887057
H = -14763.05873321
G = -14763.11626810

C	-0.498286	2.050416	-1.214253
H	-0.834693	3.084257	-1.102853
H	0.592593	2.060482	-1.185260
H	-0.790703	1.709139	-2.212096
C	0.497452	-2.381728	0.048141
C	0.608990	-0.871318	-0.163518
C	-0.826059	-0.327348	-0.300944
C	-1.105345	1.167192	-0.134316
C	-2.613067	1.385560	-0.087472
C	1.312893	-0.259607	1.042475
C	1.444089	-0.646274	-1.423120
H	1.487204	-2.842608	0.107916
H	-0.039244	-2.612599	0.972400
H	-0.041542	-2.853009	-0.778896
H	-1.225945	-0.652586	-1.270885
H	-1.432936	-0.841627	0.454153
H	-2.856049	2.437884	0.079481
H	-3.077053	1.083245	-1.031740
H	-3.076168	0.801257	0.711451
H	1.477966	0.814156	0.921423
H	0.727367	-0.402709	1.955318
H	2.290700	-0.724054	1.195922
H	1.636805	0.407245	-1.623449
H	2.414990	-1.140278	-1.326434
H	0.944111	-1.064520	-2.301192
H	-0.698057	1.484351	0.833320
I	5.609854	4.430854	-0.653674
I	3.291433	3.124326	-0.495596

$[I_2]\cdot[n\text{-}Hexane]_1$
E = -14684.70685493
H = -14684.50371887
G = -14684.55993286

C	1.333854	-2.747359	-0.000459

$[I_2]\cdot[CCl_4]_1$
E = -16335.05841132
H = -16335.03649684
G = -16335.08889145

C	0.180340	0.419052	-0.217325

C	1.298624	-1.229435	-0.000561
C	-0.112149	-0.665597	-0.000352
C	-0.147811	0.852525	-0.000250
C	-1.554104	1.427987	-0.000283
C	-1.571958	2.946248	-0.000197
H	2.356617	-3.136856	-0.001543
H	0.832844	-3.153802	0.882259
H	0.831215	-3.153894	-0.882213
H	1.836174	-0.845797	0.874290
H	1.835943	-0.845954	-0.875609
H	-0.654330	-1.043299	-0.876383
H	-0.654160	-1.043440	0.875723
H	0.397024	1.227856	0.875220
H	0.397084	1.227950	-0.875643
H	-2.096620	1.052233	-0.875523
H	-2.096724	1.052117	0.874842
H	-2.591378	3.338455	-0.000302
H	-1.062073	3.344941	0.881336
H	-1.061867	3.345029	-0.881572
I	5.109382	-1.580766	0.020348
I	7.418017	-0.254141	0.028373

Cl	0.347651	1.884280	-1.174352
Cl	1.346244	-0.789892	-0.787356
Cl	0.491501	0.774323	1.476425
Cl	-1.445405	-0.229402	-0.400952
I	4.256850	1.042572	-0.223995
I	6.272575	2.736467	0.212954

$[I_2]\cdot[\text{Chloroform}]_1$
E = -15873.34584740
H = -15873.31470337
G = -15873.36428873

C	0.214324	0.432864	-0.210492
Cl	0.289955	1.882455	-1.195178
Cl	1.367657	-0.781357	-0.779469
Cl	0.468172	0.793552	1.487264
H	-0.775800	0.003558	-0.319302
I	4.230870	1.055066	-0.222371
I	6.258242	2.742619	0.193828

$[I_2]\cdot[\text{Chlorobenzene}]_1$
E = -15141.62273881
H = -15141.51908378
G = -15141.57037740

C	-0.369956	-0.208358	-0.283450
C	-0.398623	1.150376	-0.021717
C	0.826943	-0.911008	-0.303014
C	2.010028	-0.238387	-0.053241
C	2.001055	1.124884	0.213015
C	0.794233	1.815963	0.225553
Cl	-1.847630	-1.045314	-0.586491
H	-1.342936	1.678745	-0.009406
H	0.822105	-1.973740	-0.508362
H	2.946299	-0.783443	-0.064120
H	2.930112	1.650091	0.398101
H	0.779149	2.881967	0.419564
I	1.201016	1.089075	6.063888
I	1.208705	1.190147	3.384781

$[I_2]\cdot[\text{DCM}]_1$
E = -15411.62714816
H = -15411.58738013
G = -15411.63441812

C	0.801815	0.904199	0.000664
H	0.778306	1.934401	0.341984
H	1.765296	0.442154	0.192118
Cl	-0.418806	0.004868	0.918339
Cl	0.491398	0.871281	-1.726375
I	0.899416	0.971344	3.740744
I	2.114775	1.944254	5.920726

$[I_2]\cdot[\text{Benzene}]_1$
E = -14679.89323513
H = -14679.78217387
G = -14679.82796640

C	-1.128045	-0.611912	0.262119
C	-1.130461	0.776272	0.163052
C	0.066776	-1.314325	0.141905
C	1.250895	-0.630671	-0.081623
C	1.245234	0.753557	-0.184155
C	0.057048	1.457243	-0.060255
H	-2.058977	-1.146396	0.413194
H	-2.061437	1.322766	0.258221
H	0.068399	-2.394822	0.224084
H	2.182174	-1.177180	-0.172920
H	2.173416	1.285989	-0.356649
H	0.055498	2.538280	-0.134411
I	-0.876602	-0.266365	3.386309
I	-0.847718	-0.194136	6.069328

$[I_2]\cdot[\text{2-Chlorophenol}]_1$
E = -15216.89541250
H = -15216.78619359
G = -15216.83927083

C	-0.666153	0.347891	0.090964
C	-0.691047	1.734073	0.129084
C	0.551576	-0.302050	-0.083676
C	1.727736	0.411452	-0.224793
C	1.693953	1.794828	-0.185567
C	0.483986	2.450619	-0.007670
O	-1.828778	-0.330434	0.237983
Cl	0.553679	-2.032461	-0.123057
H	-1.643451	2.230715	0.268668
H	2.659207	-0.123085	-0.361401

$[I_2]\cdot[\text{2,2,2-Trifluoroethanol}]_1$
E = -14900.77817558
H = -14900.70874268
G = -14900.75901214

I	-2.203425	0.991968	5.093633
I	-1.353290	1.287183	2.571417
C	-4.653727	2.090129	8.727267
C	-3.160863	1.878886	8.591563
O	-2.923909	0.718816	7.832820
F	-4.885241	3.212169	9.420899
F	-5.243911	2.211004	7.539240
F	-5.241953	1.081444	9.374672
H	-2.740903	2.760342	8.097521
H	-2.748095	1.809200	9.600293

H	2.613160	2.356736	-0.293129
H	0.452934	3.532917	0.024991
H	-1.642532	-1.275207	0.136076
I	-3.455844	0.016278	5.598066
I	-2.754727	-0.126771	3.018160

H	-2.300782	0.150959	8.289477

$[I_2]\cdot[\text{Toluene}]_1$
E = -14719.20517790
H = -14719.06402890
G = -14719.11526624

C	-0.914227	-0.512335	0.014985
C	-0.897668	0.872751	0.205662
C	0.300769	-1.164592	-0.148504
C	1.498970	-0.464480	-0.121499
C	1.505571	0.906611	0.074755
C	0.302080	1.576175	0.236714
C	-2.208467	-1.261587	0.013952
H	-1.836987	1.408150	0.299939
H	0.310007	-2.239031	-0.296957
H	2.434124	-0.997209	-0.250844
H	2.441055	1.452152	0.101455
H	0.290243	2.649943	0.383717
H	-2.069564	-2.294493	-0.306306
H	-2.644202	-1.277287	1.017646
H	-2.938536	-0.792215	-0.649428
I	-0.785221	0.650908	3.262445
I	-1.032247	0.336239	5.925867

$[I_2]\cdot[\text{1,2-DCE}]_1$
E = -15450.94165796
H = -15450.87174737
G = -15450.92146226

C	0.489565	-0.707262	0.400467
C	0.092715	0.662938	-0.080658
Cl	1.734286	-1.356156	-0.691982
Cl	-1.142641	1.343161	1.020578
H	0.911103	-0.670552	1.403334
H	-0.355744	-1.392857	0.382300
H	0.935026	1.351659	-0.069312
H	-0.346203	0.628069	-1.075959
I	-3.469979	-0.649785	-0.009369
I	-5.130428	-2.379714	-1.205301

$[I_2]\cdot[\text{2-Bromoanisole}]_1$
E = -17417.95117819
H = -17417.81269135
G = -17417.86974422

O	-1.750178	0.107353	-0.061404
C	-3.053065	0.641703	0.004872
H	-3.723104	-0.213385	0.060091
H	-3.180345	1.259981	0.898641
H	-3.288923	1.231528	-0.886395
C	-0.713243	0.957347	-0.037391
C	-0.823191	2.342313	-0.113457
C	0.564668	0.403038	0.072610
C	1.695036	1.193521	0.091802
C	1.571717	2.572542	0.003609
C	0.312728	3.137630	-0.094559
Br	0.736250	-1.469521	0.216497
H	-1.798914	2.802532	-0.192367
H	2.668241	0.727863	0.181121
H	2.457888	3.194329	0.017731
H	0.203467	4.213845	-0.157485
I	-1.382454	-2.022103	2.679291
I	-3.079979	-2.532915	4.705293

$[I_2]\cdot[\text{3-Bromoanisole}]_1$
E = -17417.95172470
H = -17417.81334456
G = -17417.87106391

I	-1.344054	-2.027831	2.676166
I	-3.091348	-2.554076	4.653667
C	-0.726919	0.955100	-0.012365
C	-0.872961	2.338336	-0.116572
C	0.540383	0.429689	0.094632
C	1.679290	1.219300	0.101479
C	1.511930	2.587922	-0.006887
C	0.251204	3.158006	-0.114469
Br	0.737956	-1.448881	0.236897
H	2.661722	0.776651	0.190226
H	2.384568	3.230551	-0.005336
H	0.156458	4.231813	-0.195223
O	-2.141116	2.781371	-0.215861
C	-2.350654	4.172427	-0.287107
H	-3.428538	4.312927	-0.335393
H	-1.959453	4.680828	0.600086
H	-1.890574	4.601402	-1.183345
H	-1.608595	0.328366	-0.011496

$[I_2]\cdot[p\text{-Xylene}]_1$
E = -14758.51588264
H = -14758.34743292
G = -14758.39920073

C	-1.261286	-0.747009	-0.007103
C	-1.219922	0.643473	-0.095284
C	-0.054411	-1.419046	0.114487
C	-0.022618	1.333576	-0.051160
C	1.191028	0.664012	0.080311
C	1.154655	-0.726570	0.157646
C	-2.565525	-1.481163	-0.027521
C	2.483152	1.414540	0.150582
H	-2.148847	1.196425	-0.193088
H	-0.048223	-2.501897	0.179814
H	-0.028028	2.416884	-0.114463
H	2.084947	-1.281231	0.225392
H	-2.412755	-2.560732	-0.004150
H	-3.179845	-1.212121	0.836133
H	-3.143181	-1.240837	-0.923730
H	3.335456	0.735257	0.187307
H	2.606925	2.069574	-0.715464
H	2.519512	2.044174	1.043922
I	0.984529	-0.586607	5.904920

$[I_2]\cdot[\text{4-Bromoanisole}]_1$
E = -17417.95161446
H = -17417.81314607
G = -17417.87065844

I	-1.298418	-2.047261	2.665522
I	-3.041796	-2.600883	4.642915
C	-0.719069	0.995878	-0.016129
C	-0.855108	2.364331	-0.116918
C	0.549928	0.450157	0.104822
C	1.672195	1.251073	0.128208
C	1.534422	2.628660	0.022640
C	0.268311	3.190476	-0.100728
Br	0.750029	-1.427367	0.233895
H	2.656707	0.812458	0.228004
H	2.421595	3.246453	0.040745
H	-1.593575	0.358160	-0.025803
O	0.029518	4.512840	-0.209237
C	1.134034	5.385569	-0.189918
H	0.727367	6.390686	-0.281329
H	1.690592	5.305588	0.749693
H	1.810825	5.192519	-1.028949
H	-1.833557	2.818963	-0.210835

I	0.948435	-0.685103	3.214849

$[I_2]\cdot[\text{2-Bromopropane}]_1$
E = -17190.29775360
H = -17190.19236235
G = -17190.24064829

C	-0.067485	-1.052566	-1.003272
C	-0.119116	0.448791	-0.891902
C	-1.503430	1.031848	-1.008618
Br	0.648856	0.982059	0.855598
H	0.950219	-1.429232	-0.903994
H	-0.691523	-1.524581	-0.241871
H	-0.449204	-1.346899	-1.985937
H	0.565850	0.921673	-1.595510
H	-1.492210	2.117211	-0.910604
H	-1.910163	0.779667	-1.992948
H	-2.170323	0.618668	-0.249451
I	4.881813	4.188094	-1.684519
I	2.998518	2.704564	-0.445472

$[I_2]\cdot[\text{Nitrobenzene}]_1$
E = -14884.50540160
H = -14884.38808459
G = -14884.44187881

C	-0.193808	0.307184	-0.074249
C	-0.014298	1.577124	-0.595151
C	0.850567	-0.435100	0.448962
C	2.117915	0.120350	0.448980
C	2.320365	1.394338	-0.062502
C	1.257462	2.120633	-0.582298
N	-1.538733	-0.263963	-0.078564
O	-1.710580	-1.316109	0.514147
O	-2.407544	0.332024	-0.669328
H	-0.863204	2.115278	-0.993749
H	0.665542	-1.424532	0.842160
H	2.949447	-0.443898	0.852555
H	3.314598	1.825357	-0.056159
H	1.419074	3.115771	-0.978379
I	-1.335544	-1.755215	3.371373
I	-0.930459	-2.060942	5.998101

$[I_2]\cdot[\text{Diethyl ether}]_1$
E = -14681.35520015
H = -14681.20470943
G = -14681.25612831

O	0.015435	0.025859	0.222122
C	-0.048444	1.423708	-0.010364
C	-1.485353	1.872467	0.016797
C	1.312307	-0.502593	-0.004466
C	1.255516	-2.006778	0.024923
H	0.546757	1.951605	0.747704
H	0.401359	1.634825	-0.989734
H	-1.543055	2.941730	-0.196915
H	-2.068510	1.337262	-0.734491
H	-1.935511	1.696254	0.994870
H	1.665581	-0.151548	-0.983339
H	2.006498	-0.116976	0.755116
H	2.244188	-2.417812	-0.189533
H	0.940278	-2.372371	1.003225
H	0.556030	-2.378272	-0.725879
I	-1.023669	-0.732238	5.530195
I	-0.608906	-0.417019	2.887170

$[I_2]\cdot[\text{Ethyl acetate}]_1$
E = -14755.44698941
H = -14755.31451854
G = -14755.36945001

C	0.398045	-2.228187	-0.250631
C	0.667495	-0.779700	-0.001883
O	-0.428117	-0.039056	-0.116699
O	1.750799	-0.328385	0.294407
C	-0.281123	1.367348	0.143936
C	-1.612350	2.018851	-0.107480
H	1.307360	-2.711334	-0.601076
H	0.110000	-2.687633	0.698949
H	-0.418450	-2.367541	-0.956921
H	0.049029	1.498168	1.176968
H	0.502110	1.761058	-0.507203
H	-1.537545	3.090256	0.088029
H	-1.928399	1.880458	-1.142880
H	-2.380656	1.604897	0.547634
I	2.789139	0.602779	5.611640
I	2.368962	0.213223	2.991310

$[I_2]\cdot[\text{Acetonitrile}]_1$
E = -14580.43025369
H = -14580.37447476
G = -14580.41933088

C	-0.372895	-1.229778	-0.013193
C	-0.641036	0.187551	0.098451
N	-0.853736	1.310988	0.186488
H	0.703661	-1.402954	-0.042651
H	-0.794664	-1.756794	0.843628
H	-0.821105	-1.621176	-0.927376
I	-1.311439	4.087862	0.033097
I	-1.728185	6.734101	-0.188444

$[I_2]\cdot[\text{1,4-Dioxane}]_1$
E = -14755.40469381
H = -14755.26972549
G = -14755.31838754

C	-1.187255	-0.814030	-0.803032
C	-0.861418	-1.068476	0.649716
O	-1.127292	0.563376	-1.096327
O	0.426127	-0.549878	0.955479
C	0.150618	1.074338	-0.790964
C	0.488818	0.839285	0.661954
H	-2.201292	-1.148847	-1.029898
H	-0.482191	-1.367143	-1.441122
H	-0.831401	-2.137146	0.867732
H	-1.611110	-0.592400	1.295500
H	0.910221	0.597375	-1.427866
H	0.131337	2.143703	-1.009850
H	-0.214048	1.382181	1.307792
H	1.504869	1.164967	0.890083
I	2.037849	-1.603609	6.000273
I	1.239569	-1.094696	3.478631

$[I_2]\cdot[\text{Acetic acid}]_1$
E = -14676.84687266
H = -14676.77441094
G = -14676.82001095

C	0.052596	-1.239503	0.245552
C	0.293883	0.214300	0.020528
O	-0.181803	1.118243	0.668003

$[I_2]\cdot[\text{2-Propanol}]_1$
E = -14642.06272182
H = -14641.94221689
G = -14641.99031130

C	0.063386	-0.928221	-0.107292
O	0.089209	0.503537	-0.178501
C	1.493077	-1.396676	-0.226516

O	1.136368	0.450349	-0.987143
H	0.237657	-1.815846	-0.658335
H	0.739606	-1.582045	1.023793
H	-0.962194	-1.390945	0.608505
H	1.255566	1.410132	-1.039234
I	-1.983267	0.723969	2.737964
I	-3.745614	0.203147	4.701268

H	-0.504991	-1.293670	-0.973581
H	-0.811838	0.829924	-0.093527
C	-0.606025	-1.397845	1.166538
H	-1.634533	-1.031643	1.233210
H	-0.056470	-1.040645	2.040645
H	-0.640717	-2.488991	1.200769
H	2.082461	-1.045981	0.624055
H	1.947477	-1.015360	-1.141928
H	1.532640	-2.487188	-0.246231
I	1.455034	1.972542	1.717096
I	2.691389	3.516617	3.546962

$[I_2]\cdot[\text{Ethanol}]_1$
E = -14602.75060966
H = -14602.65923939
G = -14602.70482386

C	0.058789	-0.870697	-0.074342
O	0.024995	0.549298	-0.187812
H	1.118360	-1.131613	-0.077530
H	-0.393123	-1.314407	-0.969316
H	-0.890192	0.840804	-0.128995
C	-0.616708	-1.376997	1.177273
H	-1.679204	-1.115876	1.186245
H	-0.150865	-0.953977	2.069190
H	-0.544330	-2.465667	1.233193
I	1.425475	1.971910	1.692158
I	2.738203	3.392322	3.566537

$[I_2]\cdot[\text{Nitromethane}]_1$
E = -14692.78921592
H = -14692.72889059
G = -14692.77451494

C	-0.354441	0.069572	0.411714
N	-1.713199	-0.489944	0.205389
O	-2.156570	-1.238921	1.055500
O	-2.284239	-0.187102	-0.810997
H	-0.366357	1.099008	0.065279
H	0.319076	-0.521822	-0.207614
H	-0.092578	-0.020251	1.461507
I	-1.258494	-1.609231	3.796600
I	-0.385798	-1.764707	6.323022

$[I_2]\cdot[\text{THF}]_1$
E = -14680.15252663
H = -14680.02344883
G = -14680.07127864

C	-1.129070	-0.505466	0.220454
C	1.130417	0.102277	0.120727
O	0.211732	-0.957249	0.426015
H	-1.469444	-0.836921	-0.767896
H	-1.760675	-0.966325	0.981266
H	1.683440	0.345861	1.032895
H	1.838059	-0.257852	-0.629052
C	-1.056962	1.003809	0.303622
C	0.287433	1.274104	-0.361011
H	-1.041984	1.321748	1.349432
H	-1.893148	1.495031	-0.194916
H	0.722257	2.235172	-0.085462
H	0.181995	1.250279	-1.448638
I	0.870585	-2.804366	5.429599
I	0.559064	-1.936704	2.897464

$[I_2]\cdot[\text{Methanol}]_1$
E = -14563.43875843
H = -14563.37698784
G = -14563.41988107

C	0.057911	-0.900268	-0.139463
O	0.066424	0.513969	-0.248848
H	1.093060	-1.228753	-0.218408
H	-0.344691	-1.232978	0.822121
H	-0.516977	-1.354110	-0.952186
H	-0.837171	0.836796	-0.195724
I	1.382278	1.898764	1.726625
I	2.557367	3.365980	3.651682

$[I_2]\cdot[\text{Water}]_1$
E = -14524.15077947
H = -14524.11945968
G = -14524.15865732

O	-0.175472	-0.093584	-0.146108
H	-0.170393	0.857829	-0.282782
H	0.721170	-0.387187	-0.329202
I	-0.912053	-0.643297	2.516194
I	-1.625452	-1.095462	5.063898

$[I_2]\cdot[\text{Dimethylacetamide}]_1$
E = -14735.54124570
H = -14735.39717591
G = -14735.44925575

N	0.549882	0.331282	-0.037564
C	0.531420	1.763948	0.150757
C	1.853297	-0.247393	-0.239596
C	-0.599479	-0.368433	0.020251
C	-0.544071	-1.861856	-0.160595
O	-1.681282	0.188422	0.219734
H	-0.496127	2.092583	0.274990
H	1.108503	2.037408	1.038746
H	0.972659	2.261106	-0.718000
H	1.788804	-1.316665	-0.414561
H	2.340972	0.214883	-1.103403
H	2.486342	-0.076676	0.637232
H	-1.560515	-2.236847	-0.076871
H	-0.141551	-2.136987	-1.137773
H	0.073020	-2.334415	0.606602
I	-2.253258	0.472520	2.814105
I	-2.694117	0.783921	5.458945

$[I_2]\cdot[\text{1-Butanol}]_1$
E = -14681.36262927
H = -14681.21145445
G = -14681.26324644

C	0.960931	-2.309890	-0.028583
C	0.955138	-0.791677	-0.046840
C	-0.448661	-0.215202	0.036167
C	-0.451088	1.291459	-0.003189
O	-1.796913	1.750002	0.121892
H	1.977039	-2.706398	-0.078355
H	0.496990	-2.693219	0.883940
H	0.402774	-2.714805	-0.876905
H	1.553480	-0.411789	0.788866
H	1.444499	-0.433415	-0.959913
H	-1.061822	-0.592283	-0.788922
H	-0.930331	-0.545361	0.962684
H	0.161345	1.695038	0.813294
H	-0.029793	1.647206	-0.951273
H	-1.816042	2.699566	-0.027088
I	-3.517575	1.514398	5.269547
I	-2.721171	1.566369	2.694578

$[I_2]\cdot[\text{DMSO}]_1$
E = -15002.54292825
H = -15002.45190829
G = -15002.49913234

S	-0.128469	-0.010163	-0.545293
C	-0.035675	1.756486	-0.275860
C	1.599325	-0.393685	-0.278877
O	-0.849195	-0.560054	0.650782
H	-1.055399	2.134827	-0.341681
H	0.373560	1.949452	0.716529
H	0.578703	2.210734	-1.054751
H	1.691709	-1.477289	-0.346189
H	2.200351	0.078195	-1.057686
H	1.896149	-0.052472	0.713603
I	-5.019086	-3.556162	-0.843111
I	-2.918976	-2.061469	-0.017965

$[I_2]\cdot[\text{DMF}]_1$
E = -14696.23104964
H = -14696.11526716
G = -14696.16560842

C	0.126199	-0.218382	1.077683
N	-0.027265	0.661806	0.084229
C	-1.332708	1.085572	-0.350743
C	1.119616	1.224741	-0.586043
O	1.205399	-0.617193	1.495814
H	-0.826288	-0.568535	1.504764
H	-1.451741	2.165456	-0.222906
H	-1.485315	0.844116	-1.406626
H	-2.099079	0.579167	0.236035
H	1.130645	2.312115	-0.470906
H	2.021038	0.804397	-0.146159
H	1.089444	0.987259	-1.652980
I	1.067285	-2.336963	3.481870
I	0.819969	-4.097755	5.521669

$[I_2]\cdot[\text{Acetone}]_1$
E = -14640.86014672
H = -14640.76518024
G = -14640.81226608

C	-0.006050	-1.384439	0.040166
C	0.090992	0.110091	0.073300
C	-1.187318	0.879912	-0.077601
O	1.144363	0.693822	0.218239
H	0.980015	-1.840947	0.094966
H	-0.614128	-1.727706	0.882091
H	-0.518546	-1.706082	-0.870021
H	-0.996472	1.947943	-0.000212
H	-1.643706	0.652995	-1.045038
H	-1.902629	0.570690	0.689312
I	3.594291	-0.392604	0.708575
I	5.995588	-1.478175	1.278022

$[I_2]\cdot[\text{NMP}]_1$
E = -14773.65694601
H = -14773.50372574
G = -14773.55679231

C	-1.939395	-0.834667	0.535979
H	-2.361448	-0.715051	1.535030
H	-2.643106	-1.422723	-0.051776
C	-0.553559	-1.465763	0.625176
C	-1.688198	0.541942	-0.086799
C	0.397798	-0.296206	0.564861
N	-0.292591	0.795445	0.191767
O	1.601520	-0.338267	0.792233
C	0.318296	2.062238	-0.095614
H	-0.327166	-2.122399	-0.220169
H	-0.378246	-2.039716	1.534601
H	-2.313628	1.323123	0.353214
H	-1.865759	0.548525	-1.170135
H	-0.118395	2.851949	0.520792
H	1.382689	1.990112	0.119438
H	0.180732	2.323862	-1.149626
I	3.582096	2.154906	5.108262
I	2.614163	0.918490	2.911266

$[I_2]\cdot[\text{Cyclopentane}]_1$
E = -14644.19775533
H = -14644.04516289
G = -14644.09472175

C	-1.022212	-0.602225	0.217285
C	-0.982794	0.893149	-0.065057
C	0.297960	-1.115135	-0.360205
C	0.462827	1.263865	0.251315
C	1.293319	0.057740	-0.222086

$[I_2]\cdot[\text{1,2-Dichlorobenzene}]_1$
E = -15603.34798559
H = -15603.25274518
G = -15603.30663016

C	-0.722442	0.720891	-0.228111
C	-0.673196	2.105963	-0.224988
C	0.428650	-0.022588	0.013096
C	1.626220	0.634256	0.256073
C	1.674305	2.016711	0.257195

H	-1.897227	-1.107713	-0.196680
H	-1.059714	-0.761639	1.305007
H	-1.711221	1.465092	0.513579
H	-1.194891	1.068956	-1.125750
H	0.157524	-1.367265	-1.414625
H	0.647362	-2.023539	0.134509
H	0.573655	1.394146	1.332574
H	0.775617	2.200651	-0.213779
H	2.093082	-0.171768	0.484611
H	1.774178	0.266376	-1.180064
I	0.635265	-1.186670	3.509382
I	2.081770	-1.560921	5.719784

C	0.524872	2.753813	0.016463
Cl	-2.230442	-0.058353	-0.521593
Cl	0.393437	-1.737329	0.022179
H	-1.582403	2.663295	-0.410273
H	2.514571	0.045242	0.444659
H	2.615294	2.517508	0.449362
H	0.556349	3.836341	0.018252
I	-2.898610	-1.039669	5.314535
I	-2.679108	-0.699680	2.669550

$[I_2]\cdot[\text{1-Chloro-2-methylpropane}]_1$
E = -15067.83145344
H = -15067.69483790
G = -15067.74731248

C	-0.138500	-2.199311	-0.772669
C	-0.494436	-0.723846	-0.636657
C	-1.962962	-0.537016	-0.297468
C	0.418689	-0.112565	0.405524
Cl	0.229870	1.667619	0.540293
H	0.906847	-2.338624	-1.057987
H	-0.306944	-2.732586	0.167846
H	-0.759962	-2.672052	-1.535555
H	-0.283964	-0.228346	-1.591123
H	-2.224541	0.518727	-0.211466
H	-2.593873	-0.978641	-1.071380
H	-2.206779	-1.025937	0.651056
H	0.210412	-0.504904	1.402277
H	1.468732	-0.278657	0.167481
I	1.558085	2.434486	-2.202682
I	2.726326	3.000452	-4.555689

$[I_2]\cdot[\text{m-Xylene}]_1$
E = -14758.51710428
H = -14758.34774492
G = -14758.40077604

C	-2.495927	1.058578	0.124592
H	-2.446241	2.015401	-0.399170
H	-3.326555	0.483809	-0.286487
H	-2.725587	1.273386	1.173071
C	-1.188285	-1.067196	-0.187337
C	0.015421	-1.750397	-0.260254
C	-1.207379	0.307251	0.013150
C	0.014351	0.974622	0.137096
C	1.236625	0.300666	0.060287
C	1.218499	-1.075239	-0.134295
C	2.524580	1.055416	0.160212
H	-2.124390	-1.606123	-0.283277
H	0.015206	-2.823753	-0.412737
H	0.015264	2.054986	0.252726
H	2.154789	-1.619629	-0.187675
H	3.360543	0.386486	0.367204
H	2.739664	1.579386	-0.775931
H	2.483544	1.805649	0.952778
I	0.025603	0.707521	3.138134
I	-0.028724	0.735580	5.834715

$[I_2]\cdot[\text{1-Bromo-2-methylpropane}]_1$
E = -17229.60154494
H = -17229.46537348
G = -17229.51910617

C	0.856366	-2.102040	-0.741374
C	0.588030	-0.669179	-1.190035
C	-0.902016	-0.374367	-1.218863
C	1.342780	0.258449	-0.263826
Br	1.300798	2.120248	-0.866379
H	1.922334	-2.339681	-0.760274
H	0.489714	-2.268549	0.275770
H	0.342607	-2.807285	-1.397467
H	0.994964	-0.541679	-2.199633
H	-1.104869	0.648905	-1.539671
H	-1.414047	-1.050985	-1.906165
H	-1.342241	-0.510810	-0.226119
H	0.917756	0.278950	0.739336
H	2.401933	0.016483	-0.206023
I	2.705126	3.117000	1.832941
I	3.855175	3.790856	4.176049

$[I_2]\cdot[\text{1-Bromopropane}]_1$
E = -17190.29436919
H = -17190.18748770
G = -17190.23804643

Br	-1.266387	-0.372521	1.100542
C	-0.901644	1.222537	0.029803
C	0.195413	2.059298	0.634997
C	0.484374	3.274064	-0.234348
H	-0.640349	0.840819	-0.955909
H	-1.854233	1.747561	-0.021724
H	-0.097285	2.372181	1.640704
H	1.096614	1.449942	0.740049
H	1.278616	3.879557	0.205704
H	0.808058	2.977820	-1.235251
H	-0.398452	3.909813	-0.341126
I	1.341664	-1.786024	-0.120878
I	3.540909	-2.754442	-1.344056

$[I_2]\cdot[\text{Propylene carbonate}]_1$
E = -14829.51721958
H = -14829.40080622
G = -14829.45262113

C	-0.969769	-0.002302	-0.174566
C	-0.797417	1.334385	-0.885837
C	0.667398	1.211732	0.819590
O	0.171659	-0.022066	0.696408
O	0.065103	2.065102	-0.018731
O	1.537029	1.523883	1.576481
C	-0.968175	-1.211356	-1.065927
H	-1.857665	0.007847	0.465623
H	-0.308446	1.222530	-1.857850
H	-1.727170	1.888425	-1.004201
H	-0.059397	-1.242740	-1.669986

$[I_2]\cdot[\text{Acetophenone}]_1$
E = -14832.57526375
H = -14832.42243514
G = -14832.47769898

C	0.175383	-1.177408	0.249620
C	1.319067	-1.950904	0.190469
C	0.250974	0.204957	0.085834
C	2.552538	-1.351173	-0.025739
C	2.638370	0.023217	-0.191224
C	1.491912	0.797534	-0.143022
C	-0.992644	1.009115	0.158373
C	-0.956795	2.441701	-0.291635
O	-2.036027	0.535633	0.568728
H	-0.791284	-1.634838	0.416856
H	1.250930	-3.025104	0.314913

H	-1.028014	-2.125633	-0.475129
H	-1.831690	-1.179810	-1.734539
I	3.152788	-2.030390	5.430602
I	2.400766	-0.288508	3.528262

H	3.449900	-1.957781	-0.067651
H	3.600607	0.492034	-0.359736
H	1.569492	1.869833	-0.273783
H	-1.966958	2.844091	-0.271890
H	-0.325617	3.029790	0.380551
H	-0.536630	2.530670	-1.295564
I	-2.907153	-1.221748	2.526904
I	-3.758105	-2.831097	4.502191

$[I_2]\cdot[\text{Isobutanol}]_1$
E = -14681.36492273
H = -14681.21434229
G = -14681.26568379

C	0.686773	-1.793833	-0.300410
C	0.642839	-0.284990	-0.115676
C	-0.795210	0.186247	-0.146646
C	1.341931	0.145119	1.163664
O	-0.825398	1.611679	-0.101216
H	1.718359	-2.150824	-0.309845
H	0.168949	-2.304898	0.517328
H	0.218577	-2.099531	-1.239381
H	1.152501	0.182979	-0.966996
H	-1.289011	-0.170942	-1.058694
H	-1.336196	-0.218651	0.719114
H	1.297836	1.226860	1.295605
H	0.873550	-0.323221	2.035545
H	2.392249	-0.154174	1.150162
H	-1.737127	1.901167	-0.009605
I	-0.011951	2.809727	-2.428955
I	0.669231	3.962385	-4.765494

$[I_2]\cdot[\text{Pyridine}]_1$
E = -14695.95801868
H = -14695.85749038
G = -14695.90393544

C	-1.007083	-0.688378	-0.013720
C	-1.002803	0.696856	-0.006457
C	0.202604	-1.363075	-0.001959
C	1.372271	-0.623726	0.016553
C	0.214574	1.355061	0.012207
N	1.371732	0.704073	0.023399
H	-1.942284	-1.235732	-0.028477
H	-1.924956	1.263938	-0.015226
H	0.245776	-2.444792	-0.007128
H	2.345920	-1.102418	0.026111
H	0.273412	2.438372	0.018315
I	3.609048	1.965063	0.048298
I	6.015189	3.270457	0.069985

Intermolecular complexes of an iodine molecule with two solvent molecules $[I_2]\cdot[Solvent]_2$

$[I_2]\cdot[Cyclohexane]_2$
E = -14919.35066878
H = -14918.98985831
G = -14919.05736682

C	-1.131549	-0.645743	-0.711978
C	-0.918854	0.857307	-0.829649
C	0.135953	-1.349523	-0.246937
C	-0.407510	1.448743	0.476559
C	0.860945	0.747544	0.942992
C	0.647697	-0.755413	1.057715
H	-1.936325	-0.840150	0.008385
H	-1.464356	-1.059361	-1.668485
H	-0.187831	1.055790	-1.623633
H	-1.847506	1.350387	-1.131912
H	-0.043257	-2.422560	-0.131837
H	0.907556	-1.245139	-1.020256
H	-0.225751	2.521585	0.363504
H	-1.181768	1.344042	1.247415
H	1.664539	0.944987	0.222269
H	1.194875	1.158727	1.900158
H	1.574748	-1.253803	1.370973
H	-0.077690	-0.958047	1.855656
I	6.444797	-1.500862	-1.328679
I	4.084112	-1.359711	-0.092971
C	9.670160	-3.570209	-2.406599
C	9.879748	-2.066530	-2.519810
C	10.941252	-4.269218	-1.944099
C	10.393641	-1.473037	-1.215647
C	11.663669	-2.174802	-0.754513
C	11.454307	-3.678419	-0.638277
H	8.868765	-3.770865	-1.684276
H	9.334867	-3.980802	-3.363543
H	10.603316	-1.861811	-3.318868
H	8.951181	-1.569478	-2.830227
H	10.762205	-5.342628	-1.832103
H	11.713476	-4.161708	-2.716619
H	10.570425	-0.399513	-1.329945
H	9.624305	-1.579709	-0.440342
H	12.466310	-1.977805	-1.476565
H	11.997950	-1.761734	0.201720
H	12.384617	-4.170004	-0.338696
H	10.725380	-3.879323	0.157018

$[I_2]\cdot[Iso\text{-}octane]_2$
E = -15078.96429478
H = -15078.44387569
G = -15078.53293107

C	-0.528217	2.364611	-0.251794
H	-0.422866	3.276870	0.344336
H	-0.087064	2.551901	-1.232874
H	-1.599901	2.203106	-0.404447
C	0.298220	-2.022181	-1.739450
C	0.349788	-0.502073	-1.581633
C	-0.174336	-0.177846	-0.169876
C	0.110986	1.183343	0.465146
C	-0.349453	1.158902	1.917292
C	1.792973	-0.039613	-1.753417
C	-0.521173	0.105827	-2.680366
H	0.607355	-2.322850	-2.744284
H	0.959089	-2.514987	-1.021096
H	-0.715848	-2.397948	-1.574355
H	-1.259402	-0.349176	-0.157525
H	0.248982	-0.929252	0.507678
H	-0.107561	2.095782	2.425449
H	-1.433559	1.018693	1.977876
H	0.121935	0.343510	2.471283
H	1.882341	1.048722	-1.718582
H	2.436609	-0.452639	-0.971331
H	2.187779	-0.369774	-2.718184
H	-0.494987	1.195167	-2.684334
H	-0.179222	-0.232504	-3.662600
H	-1.563980	-0.204189	-2.567319
H	1.197431	1.334605	0.468807
I	4.784867	5.532037	-0.763418
I	2.458232	4.234490	-0.575152
C	9.245552	4.481548	0.320545
H	9.263714	3.916957	1.256450
H	8.203465	4.544461	0.004408
H	9.788278	3.897534	-0.429180
C	9.367006	8.217018	-2.535730
C	8.897596	7.080903	-1.627584
C	10.102490	6.647478	-0.770751
C	9.884837	5.849373	0.515649
C	11.222941	5.694981	1.228496
C	7.760983	7.595285	-0.751391
C	8.390996	5.950723	-2.522350
H	8.562385	8.545176	-3.199554
H	9.695923	9.079880	-1.950235
H	10.205864	7.896309	-3.160115
H	10.797983	6.091539	-1.413895
H	10.636340	7.561174	-0.482285
H	11.102729	5.189151	2.189627
H	11.916482	5.100108	0.625534
H	11.692381	6.664427	1.413698
H	7.364201	6.806773	-0.104116
H	8.091889	8.408329	-0.098766
H	6.943145	7.980596	-1.367696
H	7.994378	5.109415	-1.954554
H	7.593550	6.311838	-3.178573
H	9.195721	5.571135	-3.157936
H	9.231153	6.436353	1.172571

$[I_2]\cdot[n\text{-}Hexane]_2$
E = -14921.74015291
H = -14921.33581697
G = -14921.41810235

C	1.805691	-2.985292	-0.734129
C	1.903441	-1.517465	-0.359554
C	0.553484	-0.836218	-0.209991
C	0.653191	0.630410	0.171029
C	-0.698514	1.309950	0.314592
C	-0.591779	2.774468	0.700719
H	2.792791	-3.441936	-0.834537

$[I_2]\cdot[CCl_4]_2$
E = -18222.44045861
H = -18222.40144340
G = -18222.47077017

C	0.170159	0.414514	-0.220030
Cl	0.325338	1.880080	-1.179441
Cl	1.339925	-0.787417	-0.792895
Cl	0.485338	0.774818	1.472141
Cl	-1.453811	-0.242204	-0.395518
I	4.266782	1.067647	-0.212692
I	6.316025	2.729963	0.213834

H	1.254038	-3.547078	0.024472
H	1.281452	-3.112382	-1.684988
H	2.455083	-1.416058	0.584100
H	2.481747	-0.983442	-1.125771
H	-0.002739	-0.932067	-1.150650
H	-0.036163	-1.370004	0.545742
H	1.208047	0.724415	1.113038
H	1.248185	1.162347	-0.582243
H	-1.248825	1.216500	-0.628732
H	-1.292275	0.774081	1.064254
H	-1.575870	3.239534	0.792478
H	-0.078354	2.892180	1.658936
H	-0.025812	3.338498	-0.045879
I	5.329299	-0.751615	-0.251694
I	7.983732	-0.447880	-0.219002
C	13.819308	-4.017201	0.610260
C	13.953037	-2.532295	0.322960
C	12.613139	-1.828088	0.187595
C	12.738410	-0.341505	-0.095917
C	11.399502	0.362265	-0.239687
C	11.519773	1.849565	-0.517953
H	14.795115	-4.499476	0.700288
H	13.276099	-4.190608	1.543466
H	13.269993	-4.524603	-0.187820
H	14.531585	-2.053072	1.121267
H	14.529947	-2.385650	-0.597514
H	12.033362	-2.303714	-0.613609
H	12.032139	-1.975391	1.106735
H	13.310937	0.136586	0.708601
H	13.322330	-0.192504	-1.012530
H	10.838740	-0.116772	-1.053598
H	10.820965	0.208045	0.680629
H	10.539522	2.321322	-0.615631
H	12.054821	2.356736	0.289398
H	12.070829	2.031715	-1.444430

C	8.453339	6.288186	0.229500
Cl	9.091021	4.736694	0.799528
Cl	7.042211	6.721359	1.185981
Cl	9.697931	7.519473	0.410113
Cl	8.001240	6.142984	-1.464124

[I_2]·[Chloroform]$_2$
E = -17299.01508090
H = -17298.95760627
G = -17299.02174822

C	0.199243	0.425758	-0.212745
Cl	0.263657	1.874748	-1.200255
Cl	1.354092	-0.783236	-0.784446
Cl	0.459670	0.792064	1.483420
H	-0.790252	-0.006557	-0.314935
I	4.254910	1.083096	-0.213394
I	6.312099	2.742883	0.196635
C	8.437130	6.264216	0.229843
Cl	9.099545	4.727498	0.794642
Cl	7.039927	6.732862	1.182898
H	9.207078	7.015479	0.368991
Cl	8.050801	6.189989	-1.480453

[I_2]·[Chlorobenzene]$_2$
E = -15835.56817438
H = -15835.36570200
G = -15835.43283663

C	-0.238354	-0.255235	0.066667
C	-0.373729	1.115772	0.207420
C	1.003944	-0.866994	0.140314
C	2.127312	-0.087996	0.358171
C	2.011349	1.289269	0.500439
C	0.758201	1.885633	0.424614
Cl	-1.642713	-1.221448	-0.200080
H	-1.354534	1.569803	0.151563
H	1.082133	-1.941061	0.032258
H	3.100238	-0.560523	0.420774
H	2.894463	1.896584	0.658055
H	0.659323	2.959018	0.534881
I	1.261306	0.938726	6.393627
I	1.458260	1.159336	3.719963
C	3.684142	1.188502	9.767270
C	3.549860	-0.187751	9.660390
C	2.575688	2.016363	9.813722
C	1.310023	1.452237	9.749237
C	1.157944	0.075520	9.639951
C	2.281841	-0.739278	9.597371
Cl	5.263100	1.881733	9.835406
H	4.432823	-0.812560	9.621889
H	2.704878	3.087965	9.892414
H	0.438182	2.094692	9.786168
H	0.167277	-0.360381	9.595304
H	2.171244	-1.813429	9.508813

[I_2]·[DCM]$_2$
E = -16375.57742183
H = -16375.50175936
G = -16375.56396180

C	0.788467	0.856310	-0.014288
H	0.767662	1.901591	0.277639
H	1.714718	0.377502	0.287455
Cl	-0.540676	0.036905	0.820515

[I_2]·[Benzene]$_2$
E = -14912.10847665
H = -14911.89030594
G = -14911.95014998

C	-1.138360	-0.500095	-0.128236
C	-1.008108	0.883760	-0.096935
C	-0.011577	-1.299431	-0.279975
C	1.239665	-0.716320	-0.403107

Cl	0.643702	0.754146	-1.761976
I	0.750249	1.126094	3.731183
I	2.156769	2.167323	5.766479
C	2.554343	2.258904	9.545786
H	2.766881	1.224556	9.294437
H	1.525598	2.521097	9.319517
Cl	2.832503	2.492515	11.264010
Cl	3.603385	3.281556	8.550743

C	1.368097	0.665381	-0.373498
C	0.246188	1.464907	-0.219496
H	-2.118681	-0.954501	-0.042317
H	-1.886434	1.507109	0.023333
H	-0.113221	-2.378208	-0.298653
H	2.118636	-1.339798	-0.518389
H	2.347820	1.119692	-0.466169
H	0.347902	2.543439	-0.190470
I	-0.958429	-0.229431	3.143603
I	-1.060739	-0.266785	5.832616
C	-3.268008	-1.722613	9.244124
C	-3.397436	-0.343445	9.170591
C	-2.008990	-2.305208	9.239555
C	-0.876546	-1.508516	9.164381
C	-1.003857	-0.126014	9.094334
C	-2.266716	0.455476	9.095351
H	-4.152598	-2.346513	9.299799
H	-4.381726	0.110016	9.167543
H	-1.909841	-3.383189	9.290616
H	0.107823	-1.961788	9.158417
H	-0.119056	0.498628	9.048931
H	-2.365306	1.533048	9.035154

[I$_2$]·[2-Chlorophenol]$_2$
E = -15986.11327515
H = -15985.89971395
G = -15985.97059683

C	-0.579657	0.334524	-0.069603
C	-0.696066	1.715756	-0.010161
C	0.689526	-0.231599	-0.148952
C	1.825085	0.557609	-0.173803
C	1.698714	1.934904	-0.115108
C	0.437261	2.507928	-0.032967
O	-1.703391	-0.416352	-0.034390
Cl	0.808973	-1.957588	-0.212742
H	-1.686969	2.147678	0.058095
H	2.797537	0.085794	-0.235892
H	2.585200	2.556290	-0.131997
H	0.332875	3.585083	0.015954
H	-1.449179	-1.346779	-0.114709
I	-3.470316	-0.234988	5.376033
I	-2.763366	-0.293662	2.787931
C	-5.448638	-0.863855	8.447904
C	-5.334519	-2.243740	8.359519
C	-6.688246	-0.308430	8.752210
C	-7.796070	-1.106764	8.971362
C	-7.672106	-2.482752	8.882767
C	-6.440737	-3.044946	8.576416
O	-4.353603	-0.102578	8.225600
Cl	-6.806744	1.416285	8.848632
H	-4.367767	-2.667468	8.116620
H	-8.745819	-0.643257	9.206402
H	-8.537176	-3.111615	9.051500
H	-6.338880	-4.121012	8.503664
H	-4.597620	0.825334	8.353116

[I$_2$]·[2,2,2-Trifluoroethanol]$_2$
E = -15353.88061787
H = -15353.74554749
G = -15353.81211723

C	0.001121	0.158890	-1.376682
C	0.234540	1.105278	-0.218113
O	-0.964924	1.606257	0.296255
F	1.166722	-0.263224	-1.875388
F	-0.711453	-0.910119	-1.029080
F	-0.665988	0.781670	-2.365133
H	0.729894	0.551621	0.579877
H	0.907524	1.895654	-0.569075
H	-1.432946	2.093643	-0.388350
I	-2.622464	0.893941	5.644786
I	-1.887561	1.203145	3.084253
C	-4.933470	2.106457	9.408641
C	-3.478231	2.186469	9.003624
O	-3.053781	0.912730	8.590101
F	-5.388908	3.335317	9.688463
F	-5.688457	1.600440	8.434857
F	-5.109061	1.350800	10.495996
H	-3.400023	2.915221	8.189998
H	-2.920000	2.558384	9.866189
H	-2.177634	0.738426	8.938781

[I$_2$]·[Toluene]$_2$
E = -14990.73199299
H = -14990.45364929
G = -14990.52414601

C	-0.981506	-0.401404	-0.035517
C	-0.840387	0.969551	0.188417
C	0.168900	-1.152800	-0.242312
C	1.423846	-0.561242	-0.222972
C	1.552498	0.798709	0.007598
C	0.414598	1.564660	0.212066
C	-2.335099	-1.038480	-0.020907
H	-1.726803	1.579524	0.329917
H	0.080800	-2.219683	-0.418294
H	2.306474	-1.169179	-0.385873
H	2.532156	1.260880	0.028072
H	0.499897	2.630671	0.388905
H	-2.304370	-2.047045	-0.434813
H	-2.714882	-1.108283	1.003213
H	-3.056523	-0.453628	-0.595732

[I$_2$]·[1,2-DCE]$_2$
E = -16454.20643875
H = -16454.07143662
G = -16454.13568509

C	0.437922	-0.707054	0.448443
C	-0.041994	0.604007	-0.115050
Cl	1.639213	-1.402861	-0.664333
Cl	-1.245191	1.318483	0.996822
H	0.918688	-0.572744	1.415682
H	-0.379772	-1.419724	0.541005
H	0.769783	1.322021	-0.212538
H	-0.532764	0.468775	-1.077128
I	-3.567274	-0.765953	-0.101575
I	-5.187568	-2.496733	-1.369235
C	-8.775146	-3.776180	-1.483753
C	-9.176764	-2.409677	-1.971244
Cl	-7.549312	-4.460657	-2.590346
Cl	-10.400040	-1.744287	-0.863837
H	-8.327196	-3.731454	-0.492834

I	-0.674461	0.563045	3.347280
I	-0.925412	0.141254	6.001970
C	-0.268560	0.802996	9.409131
C	-1.372537	-0.015713	9.163200
C	0.970829	0.197925	9.580037
C	1.110851	-1.180240	9.504755
C	0.009402	-1.982588	9.254938
C	-1.235582	-1.395880	9.085521
C	-0.421621	2.290580	9.454187
H	-2.353240	0.434288	9.046821
H	1.841041	0.816832	9.771587
H	2.088753	-1.628398	9.638974
H	0.118904	-3.058581	9.191451
H	-2.106421	-2.011817	8.892818
H	0.454479	2.767670	9.895004
H	-0.549741	2.693360	8.444553
H	-1.299785	2.582816	10.034007

H	-9.617573	-4.464935	-1.478240
H	-8.328485	-1.727437	-1.976358
H	-9.616928	-2.455389	-2.965681

[I_2]·[2-Bromoanisole]$_2$
E = -20388.22393111
H = -20387.95086825
G = -20388.03274599

O	-1.749498	0.104568	-0.062064
C	-3.028320	0.692788	0.003063
H	-3.736567	-0.132727	0.004017
H	-3.148578	1.273486	0.922756
H	-3.217117	1.334632	-0.863245
C	-0.677231	0.908940	-0.007676
C	-0.730057	2.299127	-0.033532
C	0.576214	0.297153	0.080270
C	1.737083	1.041101	0.125412
C	1.671524	2.426535	0.087852
C	0.437373	3.046444	0.012188
Br	0.668758	-1.583152	0.159964
H	-1.685913	2.801931	-0.092729
H	2.690194	0.532468	0.195333
H	2.582931	3.009990	0.122822
H	0.372273	4.127827	-0.011755
I	-1.632105	-1.993440	2.722807
I	-3.271187	-2.436067	4.818473
O	-2.521185	-4.352620	7.705039
C	-1.150559	-4.657250	7.587953
H	-0.628649	-3.703785	7.631106
H	-0.809796	-5.294900	8.409984
H	-0.935308	-5.143766	6.631694
C	-3.405849	-5.351897	7.571566
C	-3.070376	-6.700994	7.511368
C	-4.756846	-5.004421	7.488707
C	-5.740365	-5.963880	7.366373
C	-5.393042	-7.306230	7.318736
C	-4.059125	-7.665429	7.388130
Br	-5.233701	-3.181168	7.516776
H	-2.032556	-7.001399	7.563733
H	-6.776179	-5.656010	7.300928
H	-6.164892	-8.059264	7.221621
H	-3.774049	-8.709988	7.345030

[I_2]·[3-Bromoanisole]$_2$
E = -20388.22442057
H = -20387.95250697
G = -20388.03192413

I	-0.742012	-2.287798	2.117982
I	-2.471182	-2.920988	4.090005
C	-0.559947	0.908786	-0.185114
C	-0.935319	2.241638	-0.020813
C	0.779473	0.592269	-0.189577
C	1.771436	1.549038	-0.040900
C	1.377810	2.865473	0.115741
C	0.037238	3.224861	0.128829
Br	1.283266	-1.220522	-0.396785
H	2.816039	1.269836	-0.044769
H	2.132478	3.634279	0.234380
H	-0.236001	4.262940	0.256898
O	-2.263328	2.473662	-0.019172
C	-2.699887	3.798842	0.167943
H	-3.787391	3.763194	0.153932
H	-2.364266	4.198449	1.130494
H	-2.350313	4.453494	-0.637397
H	-1.325682	0.152697	-0.293792
C	-3.719830	-5.790679	7.321252
C	-3.797558	-7.121484	7.732157
C	-4.885568	-5.111720	7.045993
C	-6.134638	-5.703451	7.156692
C	-6.189294	-7.022657	7.567310
C	-5.037058	-7.740072	7.857339
Br	-4.784942	-3.299302	6.513092
H	-7.033299	-5.146532	6.929844
H	-7.153115	-7.508696	7.664783
H	-5.115531	-8.770096	8.175834
O	-2.617026	-7.720395	7.986986
C	-2.637246	-9.061914	8.413825
H	-1.597727	-9.346566	8.563750
H	-3.181802	-9.173101	9.357296
H	-3.085134	-9.715790	7.658426
H	-2.749843	-5.320592	7.228134

[I_2]·[*p*-Xylene]$_2$
E = -15069.35364289
H = -15069.01693278
G = -15069.09551175

C	-1.224175	-0.618448	0.185112
C	-1.072176	0.750717	0.010191
C	-0.068171	-1.388262	0.286390
C	0.184731	1.335769	-0.043070
C	1.338859	0.573678	0.073401
C	1.189837	-0.804510	0.231195
C	-2.578134	-1.246935	0.296005
C	2.697108	1.200786	0.066942
H	-1.954428	1.375980	-0.081873
H	-0.151006	-2.463378	0.408750
H	0.267800	2.409629	-0.174707
H	2.072622	-1.432259	0.299044
H	-2.602948	-2.228397	-0.181621
H	-2.853575	-1.388700	1.345758

[I_2]·[4-Bromoanisole]$_2$
E = -20388.22463993
H = -20387.95160907
G = -20388.03288677

I	-1.492962	-2.014096	2.778122
I	-3.298354	-2.378713	4.750146
C	-0.744649	0.924385	0.190042
C	-0.883225	2.293334	0.096637
C	0.526367	0.369595	0.182758
C	1.648509	1.165097	0.083022
C	1.508474	2.543387	-0.014946
C	0.239924	3.112748	-0.006895
Br	0.725945	-1.507676	0.309540
H	2.635729	0.721121	0.080745
H	2.396331	3.155597	-0.093284
H	-1.618894	0.291974	0.274716
O	-0.001742	4.437799	-0.093166
C	1.103660	5.302504	-0.194877

H	-3.345696	-0.623358	-0.165049
H	3.404615	0.616085	-0.524958
H	2.666349	2.212903	-0.338717
H	3.096260	1.261429	1.084495
I	1.110334	-0.505706	6.099782
I	0.932013	-0.692713	3.409838
C	0.870484	-2.033866	9.399159
C	0.782156	-0.645121	9.297247
C	2.137821	-2.594299	9.460053
C	1.922236	0.143708	9.243186
C	3.194344	-0.424240	9.290248
C	3.278205	-1.804170	9.408063
C	-0.364184	-2.878607	9.410543
C	4.419307	0.428922	9.183912
H	-0.194705	-0.172899	9.267830
H	2.239280	-3.671339	9.544308
H	1.820831	1.221600	9.166726
H	4.254519	-2.275644	9.454884
H	-0.130431	-3.919988	9.635015
H	-1.084329	-2.523902	10.151948
H	-0.860209	-2.848791	8.435636
H	5.305968	-0.103148	9.531568
H	4.595663	0.724677	8.145008
H	4.319525	1.345299	9.769556

H	0.696445	6.310379	-0.246159
H	1.757648	5.222134	0.679924
H	1.686491	5.103043	-1.100533
H	-1.863907	2.752830	0.104181
C	-3.515326	-5.386766	7.243970
C	-3.151997	-6.709950	7.107819
C	-4.853318	-5.037025	7.140676
C	-5.821285	-5.990626	6.907281
C	-5.455541	-7.323720	6.773121
C	-4.117200	-7.687433	6.870624
Br	-5.354880	-3.220603	7.307160
H	-6.862111	-5.704929	6.825460
H	-6.224267	-8.061537	6.589150
H	-2.762782	-4.629509	7.420718
O	-3.660136	-8.951433	6.747980
C	-4.601975	-9.970316	6.513156
H	-4.035576	-10.896898	6.443401
H	-5.321024	-10.048053	7.335729
H	-5.143119	-9.807239	5.575137
H	-2.113754	-7.009806	7.179048

$[I_2]\cdot[\text{2-Bromopropane}]_2$
E = -19932.91650769
H = -19932.70962260
G = -19932.77439276

C	0.134120	-0.887162	-1.048287
C	0.089275	0.609553	-0.878857
C	-1.287612	1.205338	-1.019045
Br	0.804613	1.071331	0.906449
H	1.146611	-1.273751	-0.933677
H	-0.514163	-1.384672	-0.324246
H	-0.219316	-1.140709	-2.052843
H	0.799078	1.099348	-1.545423
H	-1.273364	2.286169	-0.879827
H	-1.665599	0.992562	-2.024032
H	-1.979477	0.769064	-0.295809
I	5.100969	4.283070	-1.829700
I	3.226391	2.872952	-0.474841
C	9.589431	6.295944	-1.468878
C	8.202037	6.860729	-1.633184
C	8.150004	8.366490	-1.668966
Br	7.425884	6.157818	-3.310282
H	9.580447	5.206192	-1.457930
H	10.246838	6.631309	-2.273482
H	10.006281	6.646312	-0.519266
H	7.525489	6.463969	-0.876423
H	7.130183	8.729973	-1.794101
H	8.541544	8.758609	-0.724864
H	8.763234	8.761362	-2.481385

$[I_2]\cdot[\text{Nitrobenzene}]_2$
E = -15321.33364155
H = -15321.10290050
G = -15321.17863363

C	-0.302563	0.066374	0.191549
C	-0.154774	1.226887	-0.548374
C	0.764507	-0.559381	0.812364
C	2.021802	0.004808	0.686520
C	2.193601	1.168644	-0.049346
C	1.108766	1.777596	-0.665593
N	-1.638238	-0.514759	0.321474
O	-1.756037	-1.513378	1.009126
O	-2.550526	0.024294	-0.260566
H	-1.020823	1.676510	-1.013811
H	0.602831	-1.465036	1.379805
H	2.870006	-0.466634	1.167282
H	3.180846	1.605669	-0.142977
H	1.246741	2.686210	-1.238677
I	-1.351797	-1.630023	3.973483
I	-0.802199	-1.737148	6.592870
C	-1.121272	-4.392195	9.604585
C	-0.850877	-5.744690	9.719469
C	-2.383967	-3.910364	9.306857
C	-3.408779	-4.820454	9.117631
C	-3.159394	-6.181610	9.220323
C	-1.884484	-6.642262	9.520019
N	-0.028131	-3.440970	9.796053
O	-0.241332	-2.273258	9.520885
O	1.025190	-3.863405	10.212922
H	0.153056	-6.070044	9.954528
H	-2.548571	-2.845166	9.228889
H	-4.404526	-4.464195	8.884568
H	-3.964834	-6.889749	9.064941
H	-1.693422	-7.705569	9.596800

$[I_2]\cdot[\text{Diethyl ether}]_2$
E = -14915.03098331
H = -14914.73408527
G = -14914.80508394

O	0.054830	0.077921	0.013571
C	0.033536	1.476961	-0.189354
C	-1.392900	1.959867	-0.180246
C	1.344437	-0.475462	-0.160355
C	1.251185	-1.978015	-0.119055
H	0.623363	1.975193	0.593667
H	0.509926	1.704345	-1.153648
H	-1.423416	3.036015	-0.363482
H	-1.971437	1.459514	-0.958860
H	-1.866998	1.763543	0.782838
H	1.748012	-0.141621	-1.126865

$[I_2]\cdot[\text{Ethyl acetate}]_2$
E = -15063.21538995
H = -15062.95529078
G = -15063.02921948

C	0.567399	-2.164381	-0.351219
C	0.781994	-0.743651	0.062922
O	-0.335501	-0.026441	-0.021519
O	1.834928	-0.297114	0.451226
C	-0.241917	1.348615	0.384056
C	-1.581390	1.987565	0.143138
H	1.509586	-2.588677	-0.690798
H	0.231015	-2.727941	0.523114
H	-0.198405	-2.243944	-1.121023
H	0.043499	1.384402	1.437748
H	0.553766	1.827784	-0.190614

H	2.018706	-0.101424	0.623685
H	2.238422	-2.417189	-0.277352
H	0.875156	-2.320084	0.846463
H	0.580871	-2.341888	-0.899820
I	-1.025776	-0.601699	5.476779
I	-0.619048	-0.343440	2.819847
O	-1.708741	-1.050332	8.276388
C	-3.001549	-0.502154	8.442708
C	-2.910407	1.000973	8.423783
C	-1.686404	-2.452096	8.459603
C	-0.258544	-2.930712	8.465190
H	-3.665404	-0.866134	7.645142
H	-3.416422	-0.850740	9.399156
H	-3.900385	1.436609	8.574488
H	-2.251372	1.354977	9.218560
H	-2.521681	1.356827	7.468272
H	-2.176769	-2.694699	9.413127
H	-2.262832	-2.940545	7.660614
H	-0.227363	-4.009388	8.632764
H	0.229577	-2.718641	7.512474
H	0.306428	-2.440281	9.259916

H	-1.546935	3.035238	0.448324
H	-1.852414	1.946709	-0.913347
H	-2.361855	1.489100	0.720478
I	2.536070	0.595199	5.943238
I	2.293482	0.207527	3.294999
C	5.331355	0.693661	9.298750
C	4.165288	1.564497	8.956040
O	4.523297	2.837085	8.803038
O	3.034143	1.168711	8.803280
C	3.483458	3.745725	8.408484
C	4.101922	5.108801	8.265716
H	4.981118	-0.191047	9.825628
H	5.802584	0.375008	8.364860
H	6.073171	1.232415	9.885991
H	3.047898	3.393261	7.470878
H	2.697779	3.726142	9.167097
H	3.338030	5.829252	7.966963
H	4.538772	5.443091	9.208432
H	4.883964	5.104207	7.504520

[I$_2$]·[Acetonitrile]$_2$
E = -14713.17914719
H = -14713.07342520
G = -14713.12977268

C	4.926727	5.961601	1.554223
C	3.490657	6.056733	1.396933
N	2.353014	6.138028	1.269732
H	5.419407	6.725620	0.951640
H	5.198167	6.104819	2.600920
H	5.271262	4.978812	1.229987
I	-0.472130	6.063515	0.297891
I	-2.945218	6.015736	-0.761968
C	-8.190704	4.882594	-1.519199
C	-6.841836	5.401706	-1.601012
N	-5.773288	5.815325	-1.663909
H	-8.209368	3.994873	-0.885554
H	-8.854609	5.635889	-1.093241
H	-8.549181	4.615551	-2.514144

[I$_2$]·[1,4-Dioxane]$_2$
E = -15063.12932790
H = -15062.86345050
G = -15062.92892405

C	-1.346369	-0.756765	-0.866456
C	-0.893686	-1.013824	0.551698
O	-1.247171	0.613731	-1.183418
O	0.438078	-0.560166	0.731268
C	0.077746	1.062688	-1.004493
C	0.541304	0.817842	0.412346
H	-2.392882	-1.041540	-0.992528
H	-0.733394	-1.349312	-1.562081
H	-0.898129	-2.081066	0.779896
H	-1.557495	-0.496722	1.258221
H	0.748528	0.544933	-1.706453
H	0.089354	2.130473	-1.232332
H	-0.066548	1.405255	1.114272
H	1.589745	1.094079	0.537405
I	2.005537	-1.378069	5.981284
I	1.281152	-1.008868	3.408356
C	2.818807	-3.367573	8.796109
C	3.285160	-3.760147	10.177595
O	2.818272	-1.955874	8.658508
O	4.569833	-3.240054	10.439040
C	4.107682	-1.429402	8.924501
C	4.565515	-1.836284	10.305297
H	1.796711	-3.703749	8.613520
H	3.478218	-3.811249	8.037405
H	3.354125	-4.846461	10.263652
H	2.569869	-3.392095	10.928390
H	4.818154	-1.794547	8.169911
H	4.030132	-0.344056	8.839417
H	3.902530	-1.388388	11.060728
H	5.585723	-1.492392	10.487244

[I$_2$]·[Acetic acid]$_2$
E = -14906.01395633
H = -14905.87203074
G = -14905.93434781

C	0.023254	-1.167979	0.286753
C	0.222981	0.295502	0.076168
O	-0.334886	1.177640	0.680436
O	1.133552	0.557875	-0.870037
H	0.229453	-1.729095	-0.622750
H	0.719741	-1.500007	1.060999
H	-0.988123	-1.351593	0.642997
H	1.220001	1.521172	-0.914724
I	-2.162531	0.645257	2.899790
I	-3.805395	0.010083	4.940602
C	-5.527089	1.598370	8.056603
C	-6.007086	0.191171	7.931862
O	-5.614375	-0.614657	7.124883
O	-6.933794	-0.122772	8.845089

[I$_2$]·[2-Propanol]$_2$
E = -14836.44526817
H = -14836.20823175
G = -14836.27301791

C	-0.022704	-1.092699	-0.219990
O	0.007439	0.329030	-0.360698
C	1.405481	-1.569141	-0.334405
H	-0.603962	-1.507924	-1.055836
H	-0.888532	0.664453	-0.263810
C	-0.670714	-1.499463	1.087326
H	-1.698278	-1.129862	1.152292
H	-0.107710	-1.096697	1.932607
H	-0.704153	-2.587600	1.178205
H	2.005857	-1.168919	0.486187
H	1.845384	-1.237869	-1.276133
H	1.447351	-2.659195	-0.294100
I	1.407801	1.899796	1.639881
I	2.594042	3.478372	3.484224

H	-6.244767	2.220807	8.586361
H	-4.588818	1.588657	8.617051
H	-5.315427	1.997629	7.066120
H	-7.163991	-1.052260	8.701095

C	3.599210	6.588188	5.157144
O	3.947605	5.211320	5.319709
C	2.217310	6.756240	5.741054
H	3.564251	6.819230	4.083064
H	4.856589	5.092087	5.028655
C	4.628701	7.475504	5.824966
H	5.622658	7.321890	5.394417
H	4.678965	7.260646	6.895088
H	4.372167	8.529149	5.693718
H	2.226058	6.516573	6.806957
H	1.506802	6.093900	5.244002
H	1.875380	7.785390	5.616878

$[I_2]\cdot[\mathrm{Ethanol}]_2$
E = -14757.82085280
H = -14757.64209161
G = -14757.70230135

C	0.051216	-1.025468	-0.076356
O	0.030287	0.369081	-0.349209
H	1.106578	-1.278694	0.041559
H	-0.323817	-1.577975	-0.947053
H	-0.885177	0.662595	-0.373355
C	-0.726899	-1.393206	1.164966
H	-1.787185	-1.146009	1.053667
H	-0.339570	-0.861592	2.036226
H	-0.657172	-2.466987	1.355031
I	1.436134	1.959876	1.593520
I	2.720442	3.430102	3.460118
C	3.836256	6.425638	5.177432
O	4.198606	5.056980	5.302521
H	2.825630	6.492762	5.584166
H	3.782172	6.709246	4.119012
H	5.097694	4.949874	4.977689
C	4.777153	7.337447	5.928476
H	5.789721	7.285049	5.517013
H	4.820763	7.064025	6.984409
H	4.444169	8.375457	5.852368

$[I_2]\cdot[\mathrm{Nitromethane}]_2$
E = -14937.90183722
H = -14937.78318426
G = -14937.84603205

C	-0.520324	0.204964	0.645101
N	-1.748962	-0.518131	0.233601
O	-2.222688	-1.301648	1.033510
O	-2.192307	-0.298938	-0.865371
H	-0.315364	0.972151	-0.093697
H	0.280262	-0.531774	0.692771
H	-0.691512	0.612274	1.638859
I	-1.276398	-1.808054	3.789252
I	-0.343270	-2.199376	6.273112
C	-1.084631	-4.274292	9.350934
N	0.131210	-3.550297	9.796689
O	0.602532	-2.736604	9.025880
O	0.580072	-3.814103	10.883539
H	-0.802958	-4.871278	8.485043
H	-1.817100	-3.529807	9.046808
H	-1.433961	-4.890288	10.172268

$[I_2]\cdot[\mathrm{THF}]_2$
E = -14912.62432609
H = -14912.37122799
G = -14912.43304573

C	-1.115336	-0.507062	0.144625
C	1.125706	0.130459	0.013770
O	0.230164	-0.944996	0.311390
H	-1.472853	-0.795406	-0.852552
H	-1.728969	-1.009939	0.894005
H	1.716755	0.338915	0.911235
H	1.805799	-0.186334	-0.781089
C	-1.066017	0.998756	0.295177
C	0.259816	1.319367	-0.384372
H	-1.032192	1.267582	1.354336
H	-1.920113	1.499955	-0.161895
H	0.691193	2.269824	-0.068843
H	0.128328	1.352547	-1.469093
I	0.846606	-2.678069	5.524620
I	0.580422	-1.897823	2.951228
C	0.359000	-4.766235	8.399673
C	2.547661	-3.948497	8.424832
O	1.179183	-3.624881	8.160480
H	-0.103486	-4.678632	9.391151
H	-0.431060	-4.779669	7.646665
H	3.109488	-3.858403	7.489219
H	2.947275	-3.224013	9.138502
C	1.293360	-5.956705	8.332807
C	2.558606	-5.376037	8.951976
H	1.468503	-6.238704	7.291120
H	0.908928	-6.827079	8.865945
H	3.465511	-5.913578	8.673252
H	2.482422	-5.384144	10.042538

$[I_2]\cdot[\mathrm{Methanol}]_2$
E = -14679.19770078
H = -14679.07821273
G = -14679.13344114

C	0.029056	-0.985394	-0.146713
O	0.045356	0.394009	-0.464745
H	1.064192	-1.324303	-0.158250
H	-0.386221	-1.171967	0.849162
H	-0.535093	-1.561087	-0.887731
H	-0.855857	0.725880	-0.452422
I	1.375225	1.966723	1.539799
I	2.520311	3.472653	3.464929
C	3.119534	6.305418	5.729849
O	3.632756	4.989023	5.639460
H	3.491391	6.849430	4.862605
H	2.024808	6.321380	5.707583
H	3.469525	6.811108	6.635739
H	3.328217	4.485529	6.398735

[I$_2$]·[Water]$_2$
E = -14600.62233797
H = -14600.56372043
G = -14600.61276205

O	-0.034822	0.023323	-0.610116
H	-0.020278	0.979746	-0.700940
H	0.871172	-0.261420	-0.755745
I	-0.775487	-0.566132	2.163597
I	-1.449447	-1.026449	4.729803
O	-2.293360	-1.697290	7.467539
H	-3.198486	-1.393298	7.574312
H	-2.342392	-2.656681	7.487050

[I$_2$]·[Dimethylacetamide]$_2$
E = -15023.40115063
H = -15023.11518684
G = -15023.19138504

N	0.521817	0.375281	0.054065
C	0.656739	1.773491	0.387292
C	1.755932	-0.323497	-0.190661
C	-0.705005	-0.194134	0.036617
C	-0.804364	-1.661814	-0.291503
O	-1.718779	0.451539	0.284242
H	-0.333060	2.203686	0.510203
H	1.218714	1.892475	1.318564
H	1.189965	2.299377	-0.410520
H	1.576951	-1.359059	-0.462926
H	2.307861	0.155990	-1.005804
H	2.391519	-0.306803	0.701398
H	-1.858573	-1.925073	-0.274711
H	-0.394095	-1.890065	-1.277458
H	-0.276287	-2.270830	0.445765
I	-2.175926	0.660586	3.075709
I	-2.501142	0.846462	5.752219
N	-4.769739	2.600047	8.442421
C	-3.885561	3.674182	8.056913
C	-6.171404	2.926675	8.449733
C	-4.263142	1.366714	8.672397
C	-5.215492	0.270947	9.077189
O	-3.064748	1.129454	8.557753
H	-2.857164	3.347340	8.182609
H	-4.044447	3.952174	7.010401
H	-4.074152	4.550396	8.684051
H	-6.774013	2.075780	8.752159
H	-6.366859	3.751691	9.142469
H	-6.496941	3.240136	7.451803
H	-4.619075	-0.612954	9.287221
H	-5.795341	0.533473	9.964226
H	-5.914292	0.038732	8.269961

[I$_2$]·[1-Butanol]$_2$
E = -14915.04519074
H = -14914.74692564
G = -14914.81860784

C	0.916119	-2.468948	-0.441289
C	1.041796	-0.965294	-0.271933
C	-0.307882	-0.274042	-0.176810
C	-0.181299	1.222968	-0.048363
O	-1.486206	1.778816	0.060102
H	1.895446	-2.949678	-0.491534
H	0.368578	-2.913423	0.394038
H	0.375747	-2.717996	-1.358517
H	1.626872	-0.745415	0.628207
H	1.607926	-0.547819	-1.112793
H	-0.911076	-0.500842	-1.062275
H	-0.862274	-0.656395	0.687061
H	0.416247	1.477932	0.837113
H	0.328612	1.634399	-0.929121
H	-1.415440	2.737077	0.056864
I	-3.251457	1.467797	5.330845
I	-2.475980	1.491620	2.741327
C	-7.819820	4.615861	7.496043
C	-6.400531	4.498050	8.023126
C	-5.826021	3.102944	7.843089
C	-4.416139	2.987512	8.365456
O	-3.949059	1.665864	8.119931
H	-8.213683	5.625796	7.628196
H	-7.862998	4.377720	6.429958
H	-8.490707	3.925560	8.014485
H	-5.758550	5.223364	7.510472
H	-6.379399	4.769684	9.085004
H	-6.454267	2.366458	8.355102
H	-5.829105	2.834297	6.781328
H	-3.767905	3.718571	7.864061
H	-4.393174	3.198745	9.442443
H	-3.081972	1.564918	8.521285

[I$_2$]·[DMSO]$_2$
E = -15557.40198894
H = -15557.22398045
G = -15557.28691192

S	0.139981	-0.047627	-0.485694
C	0.054492	1.740575	-0.380849
C	1.892838	-0.211366	-0.143025
O	-0.562465	-0.564922	0.725125
H	-0.995403	2.007564	-0.498646
H	0.414527	2.060571	0.597975
H	0.642771	2.184880	-1.185297
H	2.107260	-1.279385	-0.115259
H	2.468093	0.261077	-0.940907
H	2.113241	0.239763	0.825441
I	-5.009045	-3.411124	-0.937517
I	-2.841789	-2.070502	-0.019644
S	-7.960135	-5.520945	-0.549669
C	-9.735310	-5.389706	-0.770320
C	-7.820707	-7.273485	-0.907587
O	-7.348701	-4.811787	-1.711871
H	-9.991307	-4.338947	-0.637684
H	-9.997281	-5.714661	-1.778185
H	-10.242711	-5.992582	-0.015352
H	-6.757969	-7.512754	-0.880941
H	-8.349068	-7.848019	-0.145032
H	-8.223812	-7.470017	-1.902162

$[I_2]\cdot[DMF]_2$

E = -14944.77860214
H = -14944.55128435
G = -14944.62035585

C	0.251653	-0.103285	1.069532
N	0.038683	0.719949	0.029846
C	-1.289732	1.091725	-0.375062
C	1.147036	1.272189	-0.706588
O	1.346184	-0.452151	1.474727
H	-0.682794	-0.450557	1.541479
H	-1.434819	2.173730	-0.294594
H	-1.478139	0.796336	-1.411925
H	-2.020535	0.595915	0.264321
H	1.146869	2.364335	-0.639831
H	2.070412	0.884160	-0.281833
H	1.083735	0.989077	-1.761456
I	1.036428	-2.310717	3.590104
I	0.460770	-4.058615	5.576663
C	1.234384	-6.281399	8.088452
N	1.440392	-7.114238	9.121870
C	2.765369	-7.505738	9.519442
C	0.327763	-7.656167	9.859504
O	0.143317	-5.915062	7.689229
H	2.172029	-5.943374	7.616197
H	2.961193	-7.219933	10.557613
H	2.896287	-8.588931	9.431090
H	3.500224	-7.014931	8.880839
H	0.398763	-7.379837	10.915685
H	-0.592103	-7.253763	9.440557
H	0.313431	-8.747819	9.786639

$[I_2]\cdot[Acetone]_2$

E = -14834.03918161
H = -14833.85326366
G = -14833.91626241

C	-0.271409	-1.306978	0.126449
C	-0.200357	0.185274	-0.017184
C	-1.493503	0.898539	-0.294664
O	0.838119	0.798522	0.083042
H	0.721884	-1.729880	0.262461
H	-0.897968	-1.561334	0.986271
H	-0.750056	-1.746984	-0.752412
H	-1.333855	1.974437	-0.316960
H	-1.898993	0.565646	-1.254413
H	-2.236320	0.644876	0.466368
I	3.431643	-0.303541	0.741622
I	5.799574	-1.434080	1.377816
C	10.722889	-2.845332	2.038438
C	9.438857	-2.065309	2.011665
C	9.553759	-0.570344	2.075677
O	8.370921	-2.629981	1.941012
H	10.514541	-3.913104	2.035458
H	11.328928	-2.585810	1.165559
H	11.311076	-2.580211	2.920976
H	8.573025	-0.105273	1.998123
H	10.025927	-0.279843	3.018606
H	10.202517	-0.207688	1.273891

$[I_2]\cdot[NMP]_2$

E = -15099.63259148
H = -15099.33027512
G = -15099.40392085

C	-2.007800	-0.775493	0.563127
H	-2.347744	-0.621824	1.589111
H	-2.796385	-1.301749	0.026231
C	-0.673260	-1.511205	0.564086
C	-1.680224	0.576852	-0.073514
C	0.359266	-0.407374	0.527521
N	-0.269472	0.739053	0.189836
O	1.554560	-0.529266	0.741033
C	0.420389	1.965642	-0.087555
H	-0.530712	-2.123789	-0.331873
H	-0.509015	-2.154025	1.427814
H	-2.251586	1.398831	0.366155
H	-1.871904	0.582968	-1.155410
H	0.064282	2.769802	0.561680
H	1.482709	1.810269	0.092582
H	0.271170	2.264789	-1.130405
I	3.499324	2.135339	5.191264
I	2.594664	0.831997	3.003480
C	8.165394	3.559481	7.636308
H	8.399970	3.939478	8.632842
H	9.031017	3.737607	6.999097
C	6.884399	4.204297	7.122370
C	7.811870	2.073718	7.727551
C	5.792751	3.241887	7.531122
N	6.378700	2.083627	7.905358
O	4.591008	3.452401	7.498703
C	5.638742	0.897993	8.228292
H	6.858368	4.273304	6.029900
H	6.684290	5.199916	7.516191
H	8.301400	1.573170	8.567466
H	8.082128	1.528763	6.812990
H	5.995171	0.471005	9.169720
H	4.588595	1.168448	8.326492
H	5.734135	0.143989	7.440534

$[I_2]\cdot[\text{Cyclopentane}]_2$
E = -14840.71923984
H = -14840.41876483
G = -14840.48411809

C	-0.843052	-0.816022	0.255565
C	-1.108781	0.667308	0.041035
C	0.494272	-1.048913	-0.448734
C	0.263237	1.297074	0.261680
C	1.262514	0.285594	-0.327878
H	-1.640304	-1.465021	-0.112831
H	-0.754791	-1.015534	1.333019
H	-1.879890	1.071990	0.699945
H	-1.438552	0.833250	-0.990834
H	0.313216	-1.284410	-1.500797
H	1.047691	-1.891991	-0.030263
H	0.437782	1.415602	1.336140
H	0.354860	2.289729	-0.183212
H	2.145436	0.187798	0.307001
H	1.618844	0.612778	-1.306934
I	1.239102	-1.102721	3.380846
I	2.760126	-1.490407	5.540567
C	4.569828	-4.789460	7.307583
C	6.040421	-4.403105	7.397501
C	3.909297	-3.919296	8.376526
C	5.989749	-2.885553	7.554984
C	4.738251	-2.618216	8.412299
H	4.388934	-5.855980	7.456516
H	4.183481	-4.527906	6.316428
H	6.627943	-4.722757	6.534295
H	6.488693	-4.860233	8.286768
H	3.967108	-4.422494	9.345477
H	2.850989	-3.740075	8.177335
H	5.878526	-2.424522	6.568879
H	6.897798	-2.471254	7.996946
H	4.172412	-1.759641	8.033820
H	5.007969	-2.357730	9.437853

$[I_2]\cdot[\text{1,2-Dichlorobenzene}]_2$
E = -16759.01885373
H = -16758.83321896
G = -16758.90592519

C	-0.723964	0.816520	-0.398421
C	-0.544870	2.188463	-0.314386
C	0.334261	-0.044861	-0.126041
C	1.568490	0.479644	0.228897
C	1.745393	1.849098	0.310901
C	0.688679	2.704629	0.039252
Cl	-2.274781	0.202580	-0.825439
Cl	0.138494	-1.747050	-0.212474
H	-1.383496	2.839809	-0.524382
H	2.382586	-0.201589	0.440879
H	2.713409	2.246292	0.590891
H	0.820464	3.777633	0.104603
I	-2.998545	-0.519522	5.151794
I	-2.822068	-0.313060	2.486681
C	-4.831446	-2.025666	8.058282
C	-4.728534	-3.406905	8.004570
C	-6.043051	-1.403083	7.774761
C	-7.146006	-2.174848	7.439406
C	-7.041530	-3.553160	7.387973
C	-5.832555	-4.170300	7.670550
Cl	-3.436639	-1.100740	8.462404
Cl	-6.196680	0.304813	7.823595
H	-3.774677	-3.869705	8.222743
H	-8.081880	-1.677856	7.218342
H	-7.909518	-4.144529	7.123131
H	-5.744036	-5.248906	7.629187

$[I_2]\cdot[\text{1-Chloro-2-methylpropane}]_2$
E = -15687.98502524
H = -15687.71569486
G = -15687.78821758

C	-0.445870	-2.174053	-0.923616
C	-0.534164	-0.706996	-0.520675
C	-1.935450	-0.340730	-0.063103
C	0.506301	-0.445171	0.548891
Cl	0.635491	1.290032	0.984301
H	0.548801	-2.431824	-1.295336
H	-0.671298	-2.828532	-0.075936
H	-1.166430	-2.396512	-1.713246
H	-0.273085	-0.093939	-1.390574
H	-2.004863	0.712570	0.213053
H	-2.659848	-0.530075	-0.857814
H	-2.229115	-0.938731	0.805560
H	0.264833	-0.965701	1.477098
H	1.501624	-0.742513	0.219959
I	1.931505	2.261092	-1.837204
I	3.061616	2.908735	-4.196428
C	1.633103	7.068838	-6.654426
C	2.194986	5.717655	-7.078026
C	1.972388	5.463677	-8.558812
C	3.662755	5.673582	-6.704665
Cl	4.390514	4.052617	-6.951394
H	1.745485	7.232951	-5.580299
H	2.138587	7.885916	-7.177603
H	0.569546	7.132803	-6.894103
H	1.686951	4.936081	-6.502416
H	2.377869	4.498191	-8.864274
H	0.906565	5.473569	-8.795501
H	2.454533	6.239998	-9.161132
H	4.252445	6.361912	-7.312849
H	3.814326	5.906758	-5.651128

$[I_2]\cdot[\text{m-Xylene}]_2$
E = -15069.35543529
H = -15069.01879102
G = -15069.09690805

C	-2.584199	0.830217	0.254308
H	-2.633047	1.769817	-0.299880
H	-3.366598	0.168784	-0.120148
H	-2.812154	1.056855	1.300722
C	-1.091154	-1.179680	0.008869
C	0.168301	-1.750836	-0.072083
C	-1.233340	0.197162	0.137338
C	-0.079333	0.979410	0.181485
C	1.197185	0.420282	0.102816
C	1.304711	-0.959094	-0.023773
C	2.411252	1.292303	0.173271
H	-1.974998	-1.807082	-0.029688
H	0.265942	-2.825743	-0.174279
H	-0.177880	2.059276	0.251135
H	2.286536	-1.415294	-0.088402
H	3.306971	0.750881	-0.133022
H	2.306162	2.171471	-0.466135
H	2.569145	1.650280	1.195432
I	-0.013352	0.803782	3.307213
I	-0.014613	0.922605	6.009846
C	-2.571222	0.903806	9.084179
H	-2.633751	-0.020781	9.661876
H	-3.348606	1.582683	9.436873
H	-2.795382	0.652660	8.042725
C	-1.058945	2.899232	9.330479
C	0.206685	3.458553	9.408727
C	-1.214393	1.525247	9.194294
C	-0.067179	0.732281	9.138760
C	1.214226	1.279169	9.213036
C	1.335152	2.657381	9.349079
C	2.422128	0.400585	9.120231
H	-1.936648	3.534618	9.378479
H	0.314361	4.531807	9.518298
H	-0.175762	-0.346162	9.063292
H	2.321340	3.104442	9.412248

H	3.295817	0.878512	9.565737
H	2.258796	-0.555212	9.621948
H	2.659347	0.183185	8.073916

$[I_2]\cdot$[1-Bromo-2-methylpropane]$_2$
E = -20011.52423525
H = -20011.25769634
G = -20011.32887462

C	0.753643	-2.122648	-0.636693
C	0.561724	-0.735444	-1.241250
C	-0.912037	-0.424437	-1.439635
C	1.247051	0.266675	-0.338678
Br	1.298299	2.064721	-1.103616
H	1.811273	-2.372891	-0.526273
H	0.285759	-2.187017	0.350041
H	0.292277	-2.881704	-1.271732
H	1.064613	-0.710862	-2.214566
H	-1.058859	0.561390	-1.883484
H	-1.376628	-1.163720	-2.095182
H	-1.441831	-0.446622	-0.482202
H	0.734543	0.379856	0.616488
H	2.291010	0.016338	-0.160704
I	2.834287	3.083961	1.670497
I	4.048644	3.685038	4.013156
C	5.802865	8.943816	6.450547
C	5.869511	7.579254	7.129509
C	5.118089	7.587472	8.449742
C	5.329126	6.549337	6.161999
Br	5.560278	4.715044	6.797374
H	6.372092	8.960417	5.518135
H	4.768656	9.215415	6.219632
H	6.209129	9.716395	7.107067
H	6.921530	7.340542	7.321945
H	5.172334	6.618758	8.949084
H	5.533159	8.339541	9.123852
H	4.062003	7.826879	8.291476
H	4.257116	6.658998	5.999177
H	5.844043	6.572498	5.203694

$[I_2]\cdot$[1-Bromopropane]$_2$
E = -19932.91004949
H = -19932.70112362
G = -19932.76753157

Br	-1.524105	-0.296859	0.880656
C	-1.001609	1.362403	-0.007245
C	0.106109	2.065794	0.734104
C	0.512509	3.339744	0.008494
H	-0.703275	1.061380	-1.010437
H	-1.914115	1.954363	-0.059798
H	-0.224561	2.295507	1.750469
H	0.963357	1.393457	0.821987
H	1.312376	3.850991	0.547140
H	0.876446	3.122656	-0.998826
H	-0.326362	4.034882	-0.080235
I	1.169789	-1.716064	-0.459061
I	3.422023	-2.572479	-1.693963
Br	6.000667	-3.947589	-3.283708
C	4.967253	-5.603554	-3.360223
C	5.007362	-6.346055	-2.049228
C	4.166205	-7.611646	-2.123279
H	3.959159	-5.291872	-3.629284
H	5.404543	-6.172596	-4.179183
H	6.043285	-6.590823	-1.800476
H	4.634202	-5.693718	-1.255490
H	4.199311	-8.151022	-1.174979
H	3.120020	-7.379032	-2.336915
H	4.526111	-8.287566	-2.903320

$[I_2]\cdot$[Propylene carbonate]$_2$
E = -15211.35349647
H = -15211.12561891
G = -15211.19543028

C	-0.922261	-0.049301	-0.255878
C	-0.976480	1.331338	-0.898120
C	0.507167	1.362765	0.799296
O	0.218288	0.069513	0.603515
O	-0.241740	2.148047	0.005684
O	1.317769	1.769624	1.570271
C	-0.741773	-1.195938	-1.209980
H	-1.793190	-0.213672	0.387326
H	-0.482225	1.348131	-1.874394
H	-1.985987	1.729045	-0.991853
H	0.155970	-1.053548	-1.814601
H	-0.652875	-2.137729	-0.668012
H	-1.605524	-1.264352	-1.875570
I	3.047675	-1.850796	5.574253
I	2.281650	-0.126680	3.657731
C	6.682548	-3.617914	9.765317
C	6.689072	-2.247398	9.100313
C	4.935065	-3.434152	8.328556
O	5.401402	-4.131139	9.375533
O	5.719719	-2.376166	8.068572
O	3.956111	-3.716103	7.712792
C	6.810979	-3.605846	11.262343
H	7.439661	-4.269874	9.316738
H	6.373594	-1.456076	9.787148
H	7.646492	-1.983832	8.653531
H	6.024219	-2.995661	11.709943
H	6.742649	-4.616763	11.664854
H	7.780325	-3.189821	11.547294

$[I_2]\cdot$[Acetophenone]$_2$
E = -15217.47083690
H = -15217.17010454
G = -15217.24681402

C	0.100902	-1.163117	0.203796
C	1.195864	-2.001554	0.115037
C	0.257386	0.215868	0.073542
C	2.460924	-1.471741	-0.102132
C	2.627090	-0.101359	-0.234841
C	1.529510	0.738711	-0.151284
C	-0.940960	1.087626	0.182274
C	-0.797621	2.554609	-0.114089
O	-2.022062	0.636607	0.502942
H	-0.890484	-1.562560	0.375583
H	1.065076	-3.072317	0.217988
H	3.319917	-2.129672	-0.168636
H	3.613878	0.313290	-0.403817
H	1.670320	1.807518	-0.255287
H	-1.776503	3.023995	-0.048679
H	-0.124506	3.023960	0.608489
H	-0.373867	2.716491	-1.107799
I	-3.060776	-1.216546	2.511269
I	-3.907703	-2.879290	4.447092
C	-7.280237	-3.301857	6.501583
C	-8.499919	-2.652221	6.494830
C	-7.186992	-4.620808	6.943047
C	-9.641976	-3.313212	6.927376
C	-9.559975	-4.625257	7.368824
C	-8.337517	-5.275495	7.379073
C	-5.859956	-5.289736	6.935369
C	-5.744894	-6.674263	7.511192
O	-4.877605	-4.741083	6.479126
H	-6.383053	-2.798024	6.164643
H	-8.562652	-1.627088	6.149408
H	-10.598765	-2.803648	6.920236
H	-10.450460	-5.142569	7.706081

H	-8.285399	-6.300342	7.725390
H	-4.705021	-6.989250	7.463865
H	-6.091399	-6.696783	8.547089
H	-6.362164	-7.376784	6.945022

$[I_2]\cdot[\text{Isobutanol}]_2$
E = -14915.04981897
H = -14914.75272747
G = -14914.82411500

C	0.675747	-1.772264	-0.397375
C	0.641351	-0.277779	-0.119693
C	-0.787233	0.219651	-0.187783
C	1.283821	0.059782	1.215790
O	-0.796251	1.634534	-0.044458
H	1.702283	-2.144184	-0.385613
H	0.116402	-2.325611	0.363754
H	0.244417	-2.011719	-1.372712
H	1.196291	0.236672	-0.913904
H	-1.235615	-0.067965	-1.147455
H	-1.376569	-0.242116	0.617003
H	1.248605	1.132125	1.410955
H	0.765522	-0.451655	2.033757
H	2.329045	-0.257345	1.234638
H	-1.707197	1.937965	-0.021115
I	0.167771	2.949275	-2.415827
I	0.908005	4.154368	-4.716907
C	-1.030652	7.926084	-7.652236
C	0.224814	7.273747	-7.095349
C	0.506374	5.989787	-7.846888
C	1.415744	8.216957	-7.144021
O	1.611613	5.328007	-7.243915
H	-1.267323	8.840739	-7.104729
H	-0.897623	8.197905	-8.704316
H	-1.896005	7.261930	-7.583141
H	0.039851	6.998315	-6.049811
H	-0.380438	5.343294	-7.826137
H	0.736098	6.221166	-8.896580
H	2.314076	7.739564	-6.751065
H	1.619424	8.528188	-8.174062
H	1.222198	9.117487	-6.556686
H	1.831954	4.552994	-7.766619

$[I_2]\cdot[\text{Pyridine}]_2$
E = -14944.22765370
H = -14944.03182322
G = -14944.09198437

C	-1.073501	-0.780943	-0.007386
C	-1.108778	0.601727	-0.078335
C	0.155623	-1.413509	0.075592
C	1.300747	-0.633444	0.083972
C	0.090895	1.295141	-0.063409
N	1.269697	0.691406	0.015985
H	-1.991377	-1.357567	-0.016719
H	-2.046810	1.138507	-0.144701
H	0.231280	-2.492130	0.132996
H	2.286090	-1.085120	0.147773
H	0.111554	2.379228	-0.117281
I	3.705981	2.109288	0.026263
I	6.102681	3.422573	0.032906
C	10.999061	6.108610	-0.007614
C	9.824909	6.791840	0.262140
C	10.936973	4.748070	-0.258532
C	9.698904	4.126904	-0.229077
C	8.634707	6.082561	0.269500
N	8.572553	4.779110	0.029268
H	11.949472	6.629542	-0.022143
H	9.825656	7.855726	0.463914
H	11.829291	4.173653	-0.473897
H	9.601719	3.062554	-0.420099
H	7.689873	6.576076	0.475683

10. REFERENCES

[1] I. M. Smallwood, *The physical properties of solvent molecules (except those for which specific references are provided) are taken from the book Handbook of organic solvent properties*; Arnold, 1996.

[2] W. M. Haynes, D. R. Lide and T. J. Bruno, *CRC Handbook of Chemistry and Physics*; 95th ed.; CRC Press, 2014.

[3] M. H. Abraham, R. J. Abraham, A. E. Aliev and C. F. Tormena, Is there an intramolecular hydrogen bond in 2-halophenols? A theoretical and spectroscopic investigation. *Phys. Chem. Chem. Phys.,* 2015, **17**, 25151−25159, DOI: 10.1039/C5CP04061B.

[4] M. Llinas and M. P. Klein, Solution conformation of the ferrichromes. VI. Charge relay at the peptide bond. Proton magnetic resonance study of solvation effects on the amide electron density distribution. *J. Am. Chem. Soc.,* 1975, **97**, 4731−4737, DOI: 10.1021/ja00849a040.

[5] A. M. Mainar, J. Pardo, F. M. Royo, M. C. López and J. S. Urieta, Solubility of nonpolar gases in 2,2,2-trifluoroethanol at 25 °C and 101.33 kPa partial pressure of gas. *J. Solution Chem.,* 1996, **25**, 589−595, DOI: 10.1007/BF00973087.

[6] J. G. Speight, *Lange's Handbook of Chemistry*; 17th ed.; McGraw Hill Education, 2017.

[7] Results of quantum-chemical calculation by PBE0 D4 ZORA-def2-TZVP method.

[8] V. A. Rana, H. P. Vankar and H. A. Chaube, Static Permittivity and Refractive Index of Binary Mixtures of 3-Bromoanisole and 1-Propanol at Different Temperatures. *J. Chem. Eng. Data,* 2015, **60**, 3113−3119, DOI: 10.1021/acs.jced.5b00256.

[9] J. George and N. V. Sastry, Densities, Viscosities, Speeds of Sound, and Relative Permittivities for Water + Cyclic Amides (2-Pyrrolidinone, 1-Methyl-2-pyrrolidinone, and 1-Vinyl-2-pyrrolidinone) at Different Temperatures. *J. Chem. Eng. Data*, 2004, **49**, 235−242, DOI: 10.1021/je0340809.

[10] E. Fischer, The electric moment of 1-methylpyrrolid-2-one. *J. Chem. Soc.*, 1955, 1382−1383, DOI: 10.1039/JR9550001382.

[11] N. V. Živković, S. S. Šerbanović, M. Lj. Kijevčanin and E. M. Živković, Volumetric and Viscometric Behavior of Binary Systems 2-Butanol + PEG 200, + PEG 400, + Tetraethylene Glycol Dimethyl Ether, and + N-Methyl-2-pyrrolidone. *J. Chem. Eng. Data,* 2013, **58**, 3332−3341, DOI: 10.1021/je400486p.

[12] C. Laurence, J.-F. Gal, *Lewis Basicity and Affinity Scales. Data and Measurement*; John Wiley & Sons Ltd, 2010.

[13] C. Reichardt and T. Welton, *Solvents and Solvent Effects in Organic Chemistry*; 4th ed.; Wiley-VCH Verlag GmbH & Co. KGaA, 2011.

[14] J. A. Dean, *Handbook of Organic Chemistry*; McGraw-Hill Book Co., 2004, New York.

[15] J. P. Perdew, K. Burke and M. Ernzerhof, Generalized Gradient Approximation Made Simple. *Phys. Rev. Lett.,* 1996, **77**, 3865−3868, DOI: 10.1103/PhysRevLett.77.3865.

[16] C. Adamo and V. Barone, Toward reliable density functional methods without adjustable parameters: The PBE0 model. *J. Chem. Phys.,* 1999, **110**, 6158−6170, DOI: 10.1063/1.478522.

[17] F. Weigend and R. Ahlrichs, Balanced basis sets of split valence, triple zeta valence and quadruple zeta valence quality for H to Rn: Design and assessment of accuracy. *Phys. Chem. Chem. Phys.,* 2005, **7**, 3297−3305, DOI: 10.1039/B508541A.

[18] J. D. Rolfes, F. Neese and D. A. Pantazis, All-electron scalar relativistic basis sets for the elements Rb-Xe. *J. Comput. Chem.,* 2020, **41**, 1842−1849, DOI: 10.1002/jcc.26355.

[19] F. Weigend, Accurate Coulomb-fitting basis sets for H to Rn. *Phys. Chem. Chem. Phys.,* 2006, **8**, 1057−1065, DOI: 10.1039/b515623h.

[20] D. A. Pantazis and F. Neese, All-Electron Scalar Relativistic Basis Sets for the Lanthanides. *J. Chem. Theory Comput.,* 2009, **5**, 2229−2238, DOI: 10.1021/ct900090f.

[21] E. Caldeweyher, C. Bannwarth and S. Grimme, Extension of the D3 dispersion coefficient model. *J. Chem. Phys.,* 2017, **147**, 034112, DOI: 10.1063/1.4993215.

[22] E. Caldeweyher, S. Ehlert, A. Hansen, H. Neugebauer, S. Spicher, C. Bannwarth and S. Grimme, A generally applicable atomic-charge dependent London dispersion correction. *J. Chem. Phys.,* 2019, **150**, 154122, DOI: 10.1063/1.5090222.

[23] F. Neese, The ORCA program system. *WIREs Comput. Mol. Sci.* 2012, 2, 73−78, DOI: 10.1002/wcms.81.

[24] F. Neese, Software update: The ORCA program system - Version 5.0. *WIREs Comput. Mol. Sci.,* 2022, **12**, e1606, DOI: 10.1002/wcms.1606.

[25] T. A. Keith and J. M. Millam, GaussView, Version 6, Roy Dennington, Semichem Inc., Shawnee Mission, KS, 2016.

[26] P. J. Hay and W. R. Wadt, Ab initio effective core potentials for molecular calculations. Potentials for K to Au including the outermost core orbitals. *J. Chem. Phys.,* 1985, **82**, 299−310, DOI: 10.1063/1.448975.

[27] W. R. Wadt and P. J. Hay, *Ab initio* effective core potentials for molecular calculations. Potentials for main group elements Na to Bi. *J. Chem. Phys.,* 1985, **82**, 284−298, DOI: 10.1063/1.448800.

[28] P. J. Hay and W. R. Wadt, *Ab initio* effective core potentials for molecular calculations. Potentials for the transition metal atoms Sc to Hg. *J. Chem. Phys.,* 1985, **82**, 270−283, DOI: 10.1063/1.448799.

[29] S. Grimme, J. Antony, S. Ehrlich and H. Krieg, A consistent and accurate *ab initio* parametrization of density functional dispersion correction (DFT-D) for the 94 elements H-Pu. *J. Chem. Phys.,* 2010, **132**, 154104, DOI: 10.1063/1.3382344.
[30] I. S. Ufimtsev and T. J. Martínez, Quantum Chemistry on Graphical Processing Units. 3. Analytical Energy Gradients and First Principles Molecular Dynamics. *J. Chem. Theor. Comp.,* 2009, **5**, 2619−2628, DOI: 10.1021/ct9003004.
[31] A. V. Titov, I. S. Ufimtsev, N. Luehr and T. J. Martinez, Generating Efficient Quantum Chemistry Codes for Novel Architectures. *J. Chem. Theory Comput.,* 2013, **9**, 213−221, DOI: 10.1021/ct300321a.
[32] W. Humphrey, A. Dalke and K. Schulten, VMD: Visual molecular dynamics. *J. Molec. Graph.,* 1996, **14**, 33−38, DOI: 10.1016/0263-7855(96)00018-5.
[33] S. Grimme, S. Ehrlich and L. Goerigk, Effect of the Damping Function in Dispersion Corrected Density Functional Theory. *J. Comput. Chem.,* 2011, **32**, 1456−1465, DOI: 10.1002/jcc.21759.
[34] T. D. Kühne, M. Iannuzzi, M. Del Ben, V. V. Rybkin, P. Seewald, F. Stein, T. Laino, R. Z. Khaliullin, O. Schütt, F. Schiffmann, D. Golze, J. Wilhelm, S. Chulkov, M. H. Bani-Hashemian, V. Weber, U. Borštnik, M. Taillefumier, A. S. Jakobovits, A. Lazzaro, H. Pabst, T. Müller, R. Schade, M. Guidon, S. Andermatt, N. Holmberg, G. K. Schenter, A. Hehn, A. Bussy, F. Belleflamme, G. Tabacchi, A. Glöß, M. Lass, I. Bethune, C. J. Mundy, C. Plessl, M. Watkins, J. VandeVondele, M. Krack and J. Hutter, CP2K: An electronic structure and molecular dynamics software package - Quickstep: Efficient and accurate electronic structure calculations. *J. Chem. Phys.,* 2020, **152**, 194103, DOI: 10.1063/5.0007045.
